# Social defeat stress responses in the stress alternative model are dependent on sex and anterior basolateral amygdala orexin 2 receptors

**DOI:** 10.1186/s13293-026-00858-0

**Published:** 2026-03-15

**Authors:** Jazmine D.W. Yaeger, Megan M. John, Leighton J. Ledesma, Trent L. Greschke, J. J. Gale, Lauren S. Meyer, Renée A. Brummels, Wayne J. Korzan, R. Parrish Waters, Cliff H. Summers

**Affiliations:** 1 Pediatrics and Rare Diseases Group, Sanford Research, Sioux Falls, SD 57104 USA; 2 Department of Biology, University of South Dakota, 414 East Clark Street, Vermillion, SD 57069-2390 USA; 3 Neuroscience Group, Division of Biomedical and Translational Sciences, Sanford School of Medicine, University of South Dakota, Vermillion, SD 57069 USA; 4 Veterans Affairs Research Service, Sioux Falls VA Health Care System, Sioux Falls, SD 57105 USA; 5 IDP in Biomedical Sciences, Medical College of Wisconsin, Milwaukee, WI 53226 USA; 6 Department of Biological and Environmental Sciences, The University of West Alabama, Livingston, AL 34570 USA; 7 Department of Biological Sciences, University of Mary Washington, Fredericksburg, VA 22401 USA

**Keywords:** α_2_ receptor, Aggression, Anxious, Orexin 2 receptor, Phenotype, Yohimbine

## Abstract

**Background:**

Comprehending sex differences in stress vulnerability and the associated neurocircuitry has been elusive until the development of a method for investigating social defeat in female mice.

**Methods:**

The Stress Alternatives Model (SAM) uses conditioning-induced agonistic behavior from novel male aggressors to separate distinct phenotypes characterized by stress-resilient active avoidance (Escape) or vulnerability (no Escape = Stay).

**Results:**

Unlike males, females predominantly display Escape behavior, which is reversible (to Stay) by adding more social stress to behavioral environments. Despite this, females exhibit both stress-resilient and vulnerable phenotypic segregation. Stress-vulnerable females exhibit double conditioning-induced corticosterone secretion, more contextual fear conditioning, and reduced social preference. Systemic and intra-BLA injections of Orx_2_R or α_2_ antagonists resulted in slower escape (Escape^S^), increased social avoidance, as well as increased cued and contextual freezing in stress-vulnerable females. Neurons in BLA express Orx_2_R mRNA (*Hcrtr2)* predominantly in cholecystokinin-positive GABA neurons. In slower escape (Escape^S^) females expression of *Hcrtr2* and *Adra2a* in BLA is elevated.

**Conclusions:**

Female and male mice exposed to social stress exhibit distinct behavioral adaptations, but similarly, separate into resilient and susceptible subpopulations. Inhibiting Orx_2_R promotes stress-vulnerable behavior in females, and modifies transcription in BLA microcircuits, suggesting a role for Orx_2_R defining stress behavior.

**Supplementary Information:**

The online version contains supplementary material available at 10.1186/s13293-026-00858-0.

## Abstract (plain language summary)

Stress, particularly during social interaction, is the primary cause of psychiatric disorders, such as anxiety and depression. While females have twice the rate of diagnosis for these disorders, understanding sexual differences in stress responsiveness has been difficult, because animal models of social defeat have been inadequate for female rodents. The Stress Alternatives Model was designed to create equally stressful aggression towards males and females, and measurements of brain and hormonal differences. In this model, sex-dependent stress response differences in physiological and molecular systems, and the variance in stress-reactivity is revealed as differences in behavior. What is more, within each sex two unique behavioral tendencies, learned during social stress, appear. Males respond in equal proportions by either escaping from an aggressive larger male (Escape; arena has two escape routes) or remaining with the aggressor (Stay). Stay can be converted to Escape, by administering anti-anxiety drugs, and drugs that increase anxiety will revert Escape to Stay. These drugs have the same effect when delivered to the region known to control fear learning. In contrast, most female mice Escape social aggression, but secrete twice as much stress hormone, with two evenly divided hormonal responses, high and low. Some females exhibit a reduction in exploration, elevated fear conditioning, and reduced sociality, compared to others. Two drugs that increase anxiety, also promote delayed escape time, lower exploration and sociability, and increase fear reactions in females, and reveal the location of their actions through gene expression of receptors in the fear learning brain region.

**Supplementary Information:**

The online version contains supplementary material available at 10.1186/s13293-026-00858-0.

## Highlights

Stress-vulnerable and resilient phenotypes are expressed differently in males and females (stay/ escape vs. slow/ fast escape phenotypes).

Compared to males, females appear to be behaviorally resilient but exhibit a high-responsive corticosterone profile.

Corticosterone concentrations in stress-vulnerable females are over twice that measured in males.

Anxiogenic treatments with Orx_2_R and α_2_R antagonists result in more *Hcrtr2* and *Adra2a* gene expression in female BLA.

In male and female mice, many *Hcrtr2* expressing BLA neurons express Cck.

**Supplementary Information:**

The online version contains supplementary material available at 10.1186/s13293-026-00858-0.

## Introduction

Differences in stress vulnerability and resilience between individuals may affect the differing rates of affective diagnoses and suicide for females and males [1–3]. Females have twice the rate of affective disorder diagnoses as males [2–4]. Since affective dysfunction and initiation of disorder development are prompted by stress [5–7], understanding behavioral and neurophysiological consequences of stress in female populations is paramount [8–14]. Models incorporating social aggression and defeat [15–18] are potent tools for delineating differential outcomes of stress-induced behavior, neurophysiology and resilience [18–20]. However, they are limited in their ability to provoke equivalent stress states in female rodent [21–23], but not primate [24], populations. Widespread use of standard defeat models results in much of our current understanding of stress neurocircuitry being biased toward the neurophysiology of males [25].

An impediment to understanding the heightened propensity of females to exhibit stress-related psychiatric conditions such as anxiety and depression is the paucity of animal models with highly translatable results [26, 27]. An important consideration is that social stress is a major impetus driving the development of these psychiatric disorders [5, 24]. Three recently developed models of social defeat for female rodents have been put forth [21–23]. While these paradigms aimed at understanding female social stress sensitivity proved to be important advances, they had limited effectiveness in producing social defeat in females, because the rate of aggression from males directed at females was low. We report a model of social stress, the Stress Alternatives Model (SAM), which produces aggressive interaction for females and males. The SAM paradigm pairs larger aggressive CD1 males with smaller female or male C57Bl/6 test mice in an oval arena equipped with tunnels that allow for the smaller mice to escape. Four daily trials with a novel aggressor produces two behavioral phenotypes in males: Escape and Stay [28–30]. The Escape phenotype is associated with stress resilience in Social Interaction/Preference testing, whereas Stay is marked by stress vulnerability, neuroinflammation measured by elevated brain and plasma TNF_α_ levels, increased fear conditioning and plasma corticosterone [31]. Pharmacological treatments are given on Day 3, with anxiogenic drugs (such as the α_2_ antagonist yohimbine) delay escape time, plus switch Escape to Stay phenotype, and anxiolytic drugs (such as the CRF_1_ antagonist antalarmin) reverse Stay to Escape [32].

Hypothalamus-derived orexins (hypocretins, Orx) mediate stress responsive states in a sex-dependent fashion, in which impaired habituation to restraint stress in females elevates corticosterone, binding to glucocorticoid receptors (GR) in the Orx promotor, and yields increased expression and activation of Orx in females plus deficits in cognitive flexibility, relative to males [33, 34]. Post-translational processing in orexinergic neurons produces two similar, yet distinct, neuromodulators: Orexin A (Orx_A_) and Orexin B (Orx_B_). Targeted cells express type 1 and 2 orexin receptor subtypes (Orx_1_R and Orx_2_R), which are activated by Orx_A_ (EC_50_ = 30 nM for Orx_1_R and 38 nM for Orx_2_R) or Orx_B_ (EC_50_ = 2,500 nM for Orx_1_R and 36 nM for Orx_2_R) activate G_q_ signaling pathways [35]. Orexinergic innervation is widespread and influences reward and arousal, but also motivation and stress tres [36–39]. Key stress circuits are constructed with pro- and anti-stress elements, including orexins, capable of shifting signal dynamics to maintain balance during periods of intense or prolonged stress [29, 40, 41]. These dynamics are particularly relevant in the context of the SAM paradigm, where we explore the impact of these circuits on stress resilience and susceptibility [20, 25]. For example, blocking Orx_1_R in the fear learning region of the brain, the basolateral amygdala (BLA), reduces anxious behavior (changes Stay to Escape) and fear conditioning; but also increases resilient behavior (likelihood to approach novel but potentially aggressive conspecifics) and motivation (time attentive to the escape route plus nose pokes) to Escape [29].

To assess the role of socially aggressive interaction toward female mice, the SAM paradigm includes classical conditioning (a brief shock during anogenital sniffing) for the CD1 aggressor mice. With CD1 male mice now aggressive toward females, we hypothesized that females would express a greater degree of stress susceptibility in response to social defeat than defeated male mice. What we discovered however, was the behavioral and physiological profile of socially stressed females were not merely enhanced compared with males, but qualitatively different from males both behaviorally and endocrinologically. We hypothesize that distinctive sex differentiated behavioral and neurophysiological responses to stressors will engage similar neurocircuitries and behavioral adaptations. These combined commonalities and differences underscore the complexity, similarity, and nuance of perception and response to stress-related conditions.

## Methods

### Animals

Adult (6–12 weeks) female (*N* = 169, weighing 18–25 g) and male (*N* = 63, weighing 23–29 g) C57BL/6 N mice (Envigo, Indianapolis) were briefly group housed (4–5 per cage for 5 days) before being individually housed on a 12:12 light-dark cycle (lights off at 6 P.M.) at 22 °C, with *ad libitum* food and water. Male Hsd: ICR (CD1) retired breeder mice (*N* = 90) were singly housed and used as aggressor mice in behavioral paradigms. Test mice (C57BL/6 N) were exposed to daily handling for 7 days prior to behavioral trials. Procedures were performed in ways that minimized suffering and the use of animals and were in accordance with the NIH’s Guide for the Care and Use of Laboratory Animals (No. 80 − 23), ARRIVE guidelines and approved by the IACUC of USD.

### Treatments

All female and male mice are primarily treated by exposure to social stress by means of the SAM. This social defeat and avoidance paradigm produces trauma in the form of active aggression, which can be relieved by escape through one of two apical L-shaped tunnels which are too small for the CD1 aggressors. In this way, two groups for each sex are formed by self-selection, Escape and Stay [30, 42–44]. Although 18 years of experimental data suggest that males are evenly split between stress vulnerable Stay and resilient Escape phenotypes (and preliminary experiments suggested that females would also fit this pattern) [27, 30–32, 44–52], individual experiments are not reliably divided 50:50. In these experiments, Stay males were 60% (*N* = 36) and Escape 40% (*N* = 27). Surprisingly, most females (85%) escaped on the first day, and 100% escaped by Day 3 (*N* = 23). Although retired breeder CD1 mice are inherently antagonistic, and no coaxing was needed to produce high levels of aggression towards male subjects, they will not attack females without further training. Thus, for interactions with females only aggressive CD1 mice were classically conditioned with a brief shock to the rump during anogenital sniffing (CCA). Because the sexes responded so differently, we tested another set of female mice (*N* = 20), adding 4 days of inescapable stress prior to the SAM interactions, which produced 50% Escape:50% Stay on Day 1, progressing to 80% Escape: 20% Stay by Day 3. We also created a control group of females subjected only to inescapable stress. Thus, for animals without drug treatment, there were 8 groups: (1) Female Escape 4-Day SAM (*N* = 15), (2) Female Stay 4-Day SAM (*N* = 1 on Day 1 only), (3) Female 2-day SAM (*N* = 16) (4) Prior stress + 4-Day SAM Female Escape (*N* = 14), (5) Prior stress + 4-Day SAM Female Stay (*N* = 6), (6 ) Inescapable stress Females (*N* = 10), (7) Male Escape (*N* = 27), (8) Male Stay (*N* = 36).

Importantly, all females subjected to SAM social interactions were also treated with vaginal lavage daily to assess estrous cycle stages. As such, each female group could also be divided by estrous phase: proestrus, estrus, metestrus, and diestrus.

Females that were injected with anxiogenic drugs were divided by injection site: subcutaneous or intracranial injections into the BLA. Subcutaneous injections included five groups: (1) Vehicle (*N* = 7), (2) 30 nmol Orx_2_R antagonist drug MK-1064 (*N* = 13), (3) 300nmol MK-1064 (*N* = 8), 1 µmol MK1064 (*N* = 7), or (4) 5 mg/kg of the α_2_ adrenoreceptor antagonist yohimbine (*N* = 7). IntraBLA injections were in five groups: (1) Vehicle (*N* = 10), (2) 6 fmol MK-1064 (*N* = 7), 15 fmol MK-1064 (*N* = 6), 30 fmol MK-1064 (*N* = 8), or 60 fmol MK-1064 (*N* = 7).

### Behavioral paradigms

**Fig. 1:**
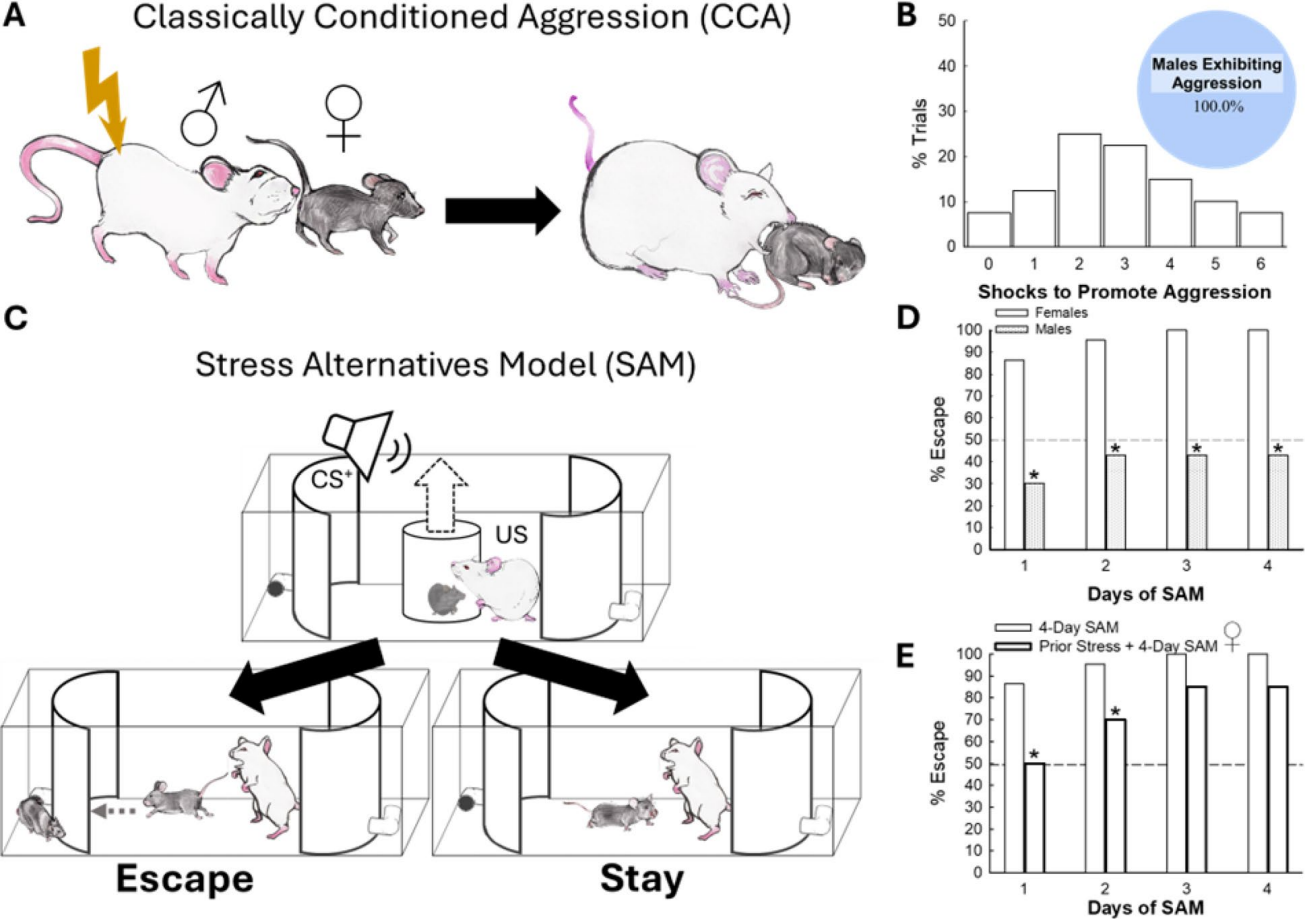
Social stress establishes sex-distinctive Escape behavioral patterns. **(A)** Classically conditioned aggression (CCA) involves applying a mild shock (0.04 mA, < 1 s) to male (CD1 retired breeder) mice as they perform anogenital sniffing of female conspecifics. **(B)** The number of shocks needed to promote aggression varies, but all preliminary trials (*N* = 40) resulted in aggressive interactions. **(C)** The Stress Alternatives Model (SAM) is a 4-day social stress paradigm in which mice are conditioned (Tone, cued or CS^**+**^) to social aggression (US) and commit to a behavioral phenotype by the end of Day 2: Escape (active avoidance of social aggression) and Stay (accepting confrontation from aggressor). **(D)** Female mice experiencing social aggression in the standard 4-Day SAM all escaped by Day 3; whereas ~ 45% of male mice committed to the Escape phenotype by the end of Day 2 (*N* = 85, Day 1: Χ^2^ = 18.6, *p* < 0.001; Day 2: Χ^2^ = 16.3, *p* < 0.001; Day 3 & 4: Χ^2^ = 19.5, *p* < 0.001). **(E)** In female mice more social stress results in more expression of the Stay phenotype. Female mice that experienced 4 days of social stress prior to SAM introduction exhibited more Stay behavior (*N* = 42, Day 1: Χ^2^ = 4.9, *p ≤ 0.03; Day 2: Χ^2^ = 3.2, *p ≤ 0.04)

The SAM uses a 4-day (5 min/day) experimental plan that starts with test animals (C57BL/6 N mice) being introduced to an opaque cylinder in the center of an oval-shaped, open field arena (Figs. 1C, S1). An aggressor (male CD1 mouse) roams the arena outside of the cylinder. After a 30 s resting period, a 5 s tone (cued conditioned stimulus) and 10 s trace precede the lifting of the cylinder divider allowing the aggressive CD1 mouse to attack (unconditioned stimulus, US) the test animal. This model does cause an increase in neuroinflammation with elevated plasma and brain TNF_α_, also found in anxiety and depression, but we take great care to ensure that injuries due to aggression are not inflicted [31]. Importantly, no test mouse encounters the same CD1 mouse twice throughout the 4-Day paradigm. In the SAM arena, two escape routes (inner diameter = 3.8 cm) existing on opposite apical ends of the open field space allow the smaller test mouse to escape aggressive encounters without possibility to return to arena. In male mice, the end of the second day marks a commitment to one of two stable behavioral phenotypes: Escape or Stay. Escape phenotype mice commit to approaching, entering, and passing all the way through L-shaped tunnel at the apical end of the SAM social arena, typically beginning on Day 1, and continuing for all of four consecutive trials. In contrast, Stay phenotype mice do not approach the escape route, and never enter and pass through the tunnel, but remain in the social arena interacting with the CD1 aggressor for 300 s in each of four daily trials. This commitment allows for pharmacological intervention on Day 3, which may reverse the chosen phenotype. Anxiogenic drugs delay escape time and promote Stay behavior, whereas anxiolytic drugs initiate escape behaviors in Stay mice. A purpose of the following studies was to investigate how female behavior in the SAM differs from that in males.

To explore female behavior with our developed Classical Conditioned Aggression (CCA) method, we exposed female C57Bl/6 N mice to several unique paradigms (Figs. S1, S2). A group of female mice (*N* = 22) were presented with the standard 4-Day SAM paradigm. As the male-appropriate Escape and Stay phenotypes were potentially irrelevant in females, we designed experiments to assess stress-coping-related phenotypes using the stress hormone corticosterone and the high and low responder model as a guide [53–55]. We paired that with a model of contextual and cued fear conditioning which is an integrated part of the SAM protocol, after which we took tissue and plasma samples. Contextual and cued fear conditioning were paired with a classic method for rodent social defeat models which produce stress-vulnerable and resilient phenotypes, based on time spent near a social target after social defeat in SIP testing [16, 17]. We examined stress hormone data to determine if the distribution of corticosterone concentrations suggested clearly separated phenotypic groups, and if these groups were significantly different in Fear conditioned Response (CR) and Social Interaction/Preference (SIP) results.

Additionally, a separate cohort (*N* = 10) were introduced into a SAM-like setting, but without escape routes for four days (Inescapable Social Stress group). As our preliminary results suggested all females introduced into the SAM escaped, we developed an experimental plan in which a cohort of females (*N* = 16) was presented with a shorter SAM protocol (2-Day SAM group). Finally, to see how elevated stress levels might affect SAM behavior, we developed a group of females (*N* = 20) that were presented with brief bouts of aggression (less than 1 min) in a male CD1 home cage for four days preceding the standard 4-Day SAM paradigm (Prior Stress + 4-Day SAM). Separate experimental designs, involving both female (*N* = 106) and male (*N* = 17) mice, utilized the standard 4-Day SAM paradigm with subcutaneous (sc) or iBLA drug administration on Day 3 (Fig. S2).

In all experiments reported here, the behavioral paradigms were followed by the SIP and fear CR tests. Both tests have been used by our lab previously [28, 56–58]. In brief, the SIP test involves introduction of a test mouse to a square (40 cm^2^) open field environment. An empty perforated jar (Novel target) was positioned alongside a wall and the test mouse was allowed to explore this area, including the Novel target, for 2.5 min. The mouse was briefly removed, and the empty jar was replaced by an identical jar that contained an aggressive CD1 mouse (Social target) not previously used in preceding behavioral trials. Again, the test mouse was allowed to explore the environment and social target for 2.5 min. More time spent within 3 cm of the Social target compared to the Novel target was considered social preference behavior, and increased time spent in the corners when the Social target was present is social aversion behavior. Fear Response (CR) is tested by placing test mice within the same cylinder divider used during daily fear conditioning (context, prior to the tone) and measuring freezing for 30 s. Afterwards, freezing is measured during a 5 s tone (2500 Hz at 75 dB) and 10 s trace period (cue, including/following the tone). Importantly, during the Fear Response test, no CD1 aggressor is present. At the end of the Fear Response test, mice were briefly anesthetized (5% isoflurane, 2 min) and rapidly decapitated. Trunk blood was spun down (5 min) in heparinized tubes and plasma was collected. Brains were extracted and flash frozen in cold isopentane on dry ice.

All behavioral experimentation was performed during the animals’ active/awake period during darkness (scotophase) under red light. Each behavioral trial was recorded using GoPro (Hero 7, Hero 8, or Hero 9) cameras. Videos were analyzed using ANY-maze (version 6.0) software.

**Classically conditioned aggression (CCA)** Classical Pavlovian conditioning was used to train male CD1 mice to behave aggressively toward female C57Bl/6 N mice. No conditioning was necessary to provoke aggression against C57Bl/6 N male mice. This approach was designed to result in equivalent aggression towards males and females by CD1 males, thus the number of shocks a CD1 received was sufficient to reach this threshold. Preliminary experiments to determine the quantitative or qualitative differences in aggression received by males and females were undertaken. These experiments measured (1) Latency to attack, (2) Comprehensive Aggression, including chases, scratches, pinning, pulled fur, attempted bites and bites (also the area of biting: head, neck, rump, belly), and (3) Total number of bites (total and average per individual over 4 days) in an arena without escape routes. While within individual males and females there were differences in aggression received over time, on average, there were no quantitative nor qualitative differences between CD1 attacks on males, and conditioned CD1 attacks on females. Neither male CD1 aggressive behavior, nor female C57Bl/6 N responses, correlated with pre-SAM number of shocks, since that was designed to be standardized.

Male CD1 mice, investigating (specifically anogenital sniffing) female mice introduced into the males’ home cage, were delivered a brief mild shock (0.04 mA, 10 V, < 1 s) to the unshaven rump region via shock device (NVK small dog shock training collar) attached to prong created from 18 G copper wire and delivered by an investigator following an average latency of 1 s, resulting in intense aggression directed toward the female mice (Fig. 1A, B). Once aggression was displayed by the CD1, mice were separated. Typical interactions lasted under 5 min, as aggression was often immediately obtained through the pairing of the female and mildly shocked male aggressor. This procedure was repeated for 4 days prior to the use of the male CD1 mice in behavioral paradigms. Novel control C57Bl/6 N male and female mice were paired with a CD1 aggressor each day of aggression screening. All CD1 mice positively screened for aggression were used during behavioral testing for male and female mice, but CCA was only used toward CD1 aggressors in the SAM apparatus when paired with a female test mouse. In the experimental paradigm environments, as CD1 mice have been trained to associate a female mouse (scent) with a mild shock, the use of a shock was sometimes unnecessary as many male mice displayed aggression without the shock. If a CD1 mouse did not behave aggressively toward the female during experimentation, a mild shock was applied to produce aggressive behavior.

### Estrous cycle

For all female mice, vaginal lavage was performed daily based on a previously described protocol [59] with modifications. In brief, 50 µL of distilled water (dH_2_O) was gently flushed 3–6 times into the vaginal cavity and the contents were placed onto microscope slides. Samples were dried under a heat lamp and stained for 1 min with Cresyl violet (0.1 g/100 mL dH_2_O), rinsed twice for 1 min with dH_2_O, and air dried. The samples were viewed under a microscope and the stages of estrous were identified by the abundance of three distinct cell types (Figs. S5-8): proestrus samples included mostly round nucleated epithelial cells, estrus samples were characterized by dense clusters of cornified squamous epithelial cells, metestrus samples contained predominantly leukocytes with few cornified epithelial cells, and diestrus samples involved a mix of all cell types and was distinguished from metestrus by the presence of nucleated epithelial cells. As our preliminary results do not definitively expose whether the stage of the estrous cycle might impact stress-related behaviors, for drug treatment studies (Fig. S6, S8) we only started female animals through behavioral the paradigm when they were in proestrus.

**Fig. 2:**
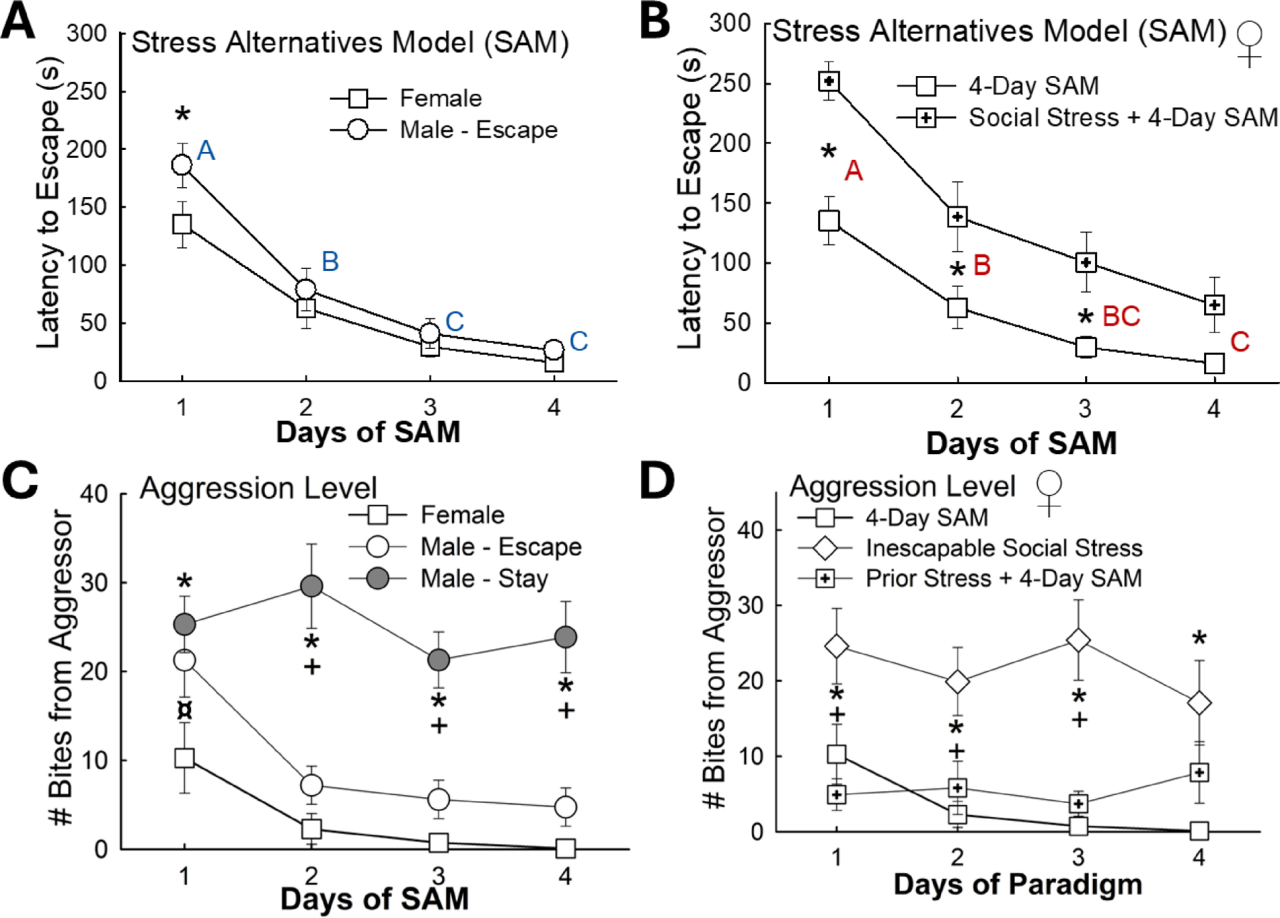
Escape plus latency to escape influences aggression received** (A)** Male mice that chose the Escape phenotype experienced a slower latency to escape on Day 1 compared to female animals (N = 49, Day Effect: F_3,141_ = 51.8, *p*< 0.001; Day 1: t_47_ = 2.4, *p≤ 0.02; A, B, C reflect significant differences based on time, i.e. Days, when letters are not the same, e.g. A is different from B, such that Day 1 latency is greater than Day 2, *p* ≤ 0.02). **(B)** Latency to escape in females that experienced social stress prior to SAM exposure displayed slower escape times on Days 1-3 (N = 42, Paradigm Effect: F_1,120_= 12.9, *p* < 0.001; Interaction Effect: F_3,120_= 2.3, *p *≥ 0.085; Day Effect: F_3,120_= 51.0, *p* < 0.001; Day 1: t_40_= 4.3, *p < 0.001; Day 2: t_40_ = 2.8, *p< 0.001; Day 2: t_40_ = 2.6, *p≤ 0.01; unique letters indicate differences from other Days, e.g. A is different from B, *p* < 0.001). **(C)** Male Stay mice experience more aggression than Escape mice, which receive similar levels of aggression as Female mice in the SAM arena (N = 85, Phenotype Effect: F_2,192_= 42.9, *compared to females or ^+^compared to male/Escape *p* < 0.001; Interaction Effect: F_6,192_ = 1.5, *p* ≥ 0.175 ; Day Effect, females < Stay males: F_3,192_= 5.1, ^¤^p ≤ 0.002). **(D)** Females in an inescapable social stress paradigm receive more aggression than females exposed to the SAM where they can avoid aggressive encounters (N = 52, Paradigm Effect: F_2,147_ = 32.4, *compared to 4-Day SAM or ^+^compared to Prior Stress+4-Day SAM *p* < 0.001; Day Effect: F_3,147_ = 1.2, *p*≥ 0.295; Interaction Effect: F_6,147_ = 1.4, *p *≥ 0.229)

**Fig. 3:**
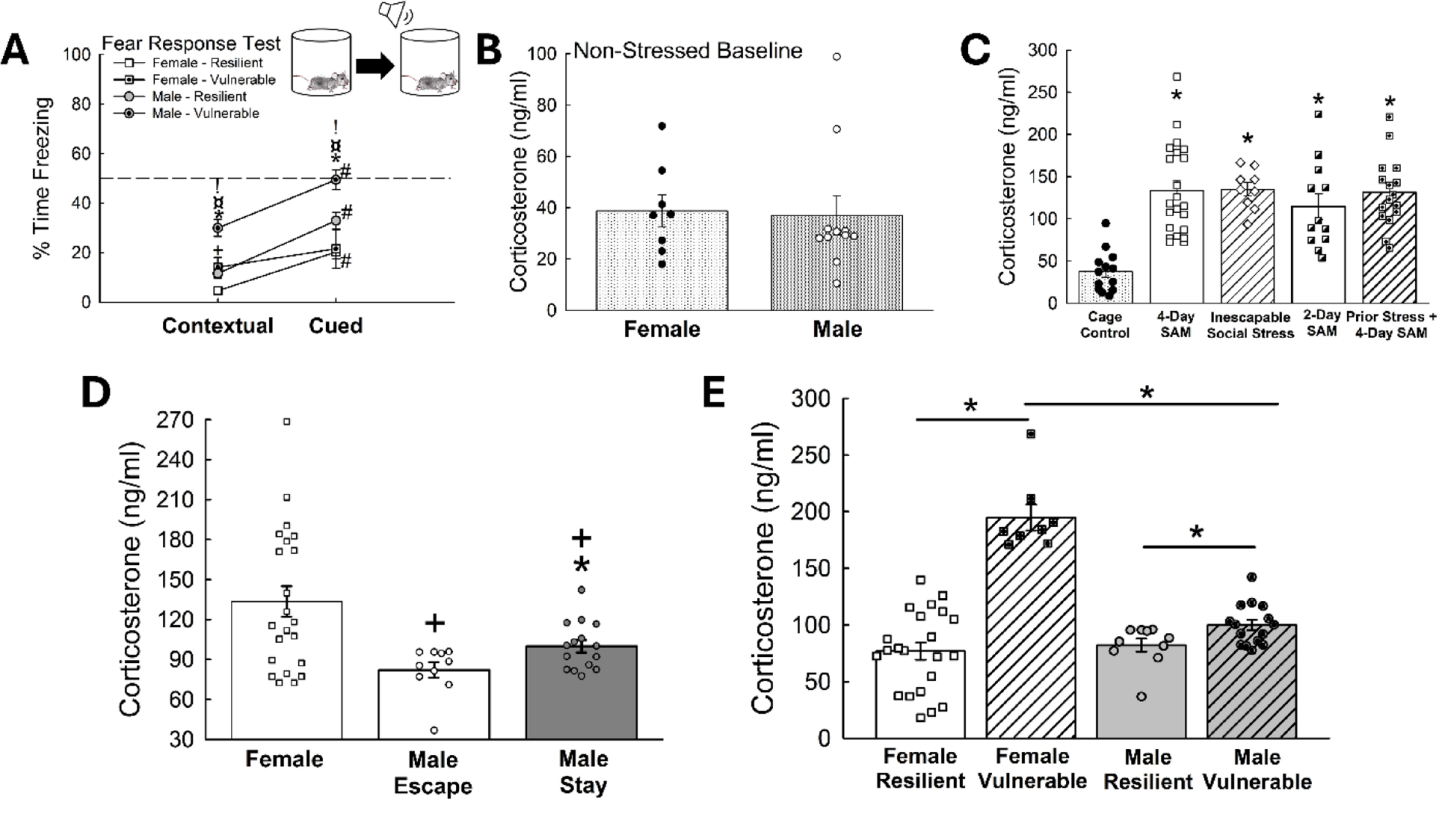
Female stress-related phenotypes exhibit dissociable endocrine stress responses. **(A)** Vulnerable female and male mice (standard 4-Day SAM protocol) exhibit elevated contextual and cued fear freezing behavior (N = 97; Contextual, Phenotype Effect: F_1,94_ = 10.3, *p* ≤ 0.002). Female mice display the lowest cued fear response (Sex Effect: F_1,94_ = 7.0, *p* ≤ 0.010; Female vs Male – Vulnerable, t_53_ = 1.9, ^!^*p* ≤ 0.05; Male – Resilient vs Vulnerable, t_74_= 4.1, *p < 0.001; Female vs Male – Resilient, t_41_ = 2.4, ^¤^*p*≤ 0.023; Female vs Male – Vulnerable, t_53_ = 1.9, ^!^*p*≤ 0.05; Female – Resilient vs Vulnerable, t_20_ = 2.6, ^+^*p*≤ 0.019; Cued, Sex Effect: F_1,94_ = 13.4, *p *< 0.001; Male – Resilient vs Vulnerable, t_74_= 2.9, *p ≤ 0.004; Resilient – Male vs Female, t_41_ = 2.4, ^¤^*p *≤ 0.020; Vulnerable – Male vs Female, t_53_ = 2.9, ^!^*p* ≤ 0.006; Contextual vs Cued, Male – Resilient, t_29_ = 5.8, ^#^*p*< 0.001; Male – Vulnerable, t_45_= 7.3, ^#^*p* < 0.001; Female – Resilient, t_12_= 5.9, ^#^*p *< 0.001). **(B)** Unstressed mice (N = 19, t_17_= 0.2, *p* ≥ 0.865). **(C) **Stressful environments raise plasma corticosterone concentrations in female mice, but not between various social stress paradigms (N = 82, F_4,77_= 8.9, *p* < 0.001). **(D)** After SAM exposure, corticosterone levels are highest in females (N = 47, F_2,44_ = 6.9, *p* ≤ 0.003; Female vs Male – Escape, t_30_ = 2.9, ^+^*p* ≤ 0.007; Female vs Male – Stay, t_35_ = 2.3, ^+^*p* ≤ 0.026; Male – Escape vs Stay, t_23_ = 2.4, *p ≤ 0.025), but **(E)** diverge. Vulnerability/resilience emerge from disparate corticosterone responses, such that vulnerable females display stress hormone concentrations nearly twice as high as resilient females and all males (N = 31, F_1,51_= 36.6, *p* < 0.001; Resilient Female (low corticosterone) vs Male (Escape), t_30_ = 0.447, *p* ≥ 0.658; Vulnerable Female (high corticosterone) vs Male (Stay), t_21_ = 9.1, *p<0.001; Male Resilient vs Male Vulnerable, t_23_ = 2.4, *p ≤ 0.025; Female Resilient vs Female Vulnerable, t_28_ = 8.2, *p < 0.001)

**Fig. 4:**
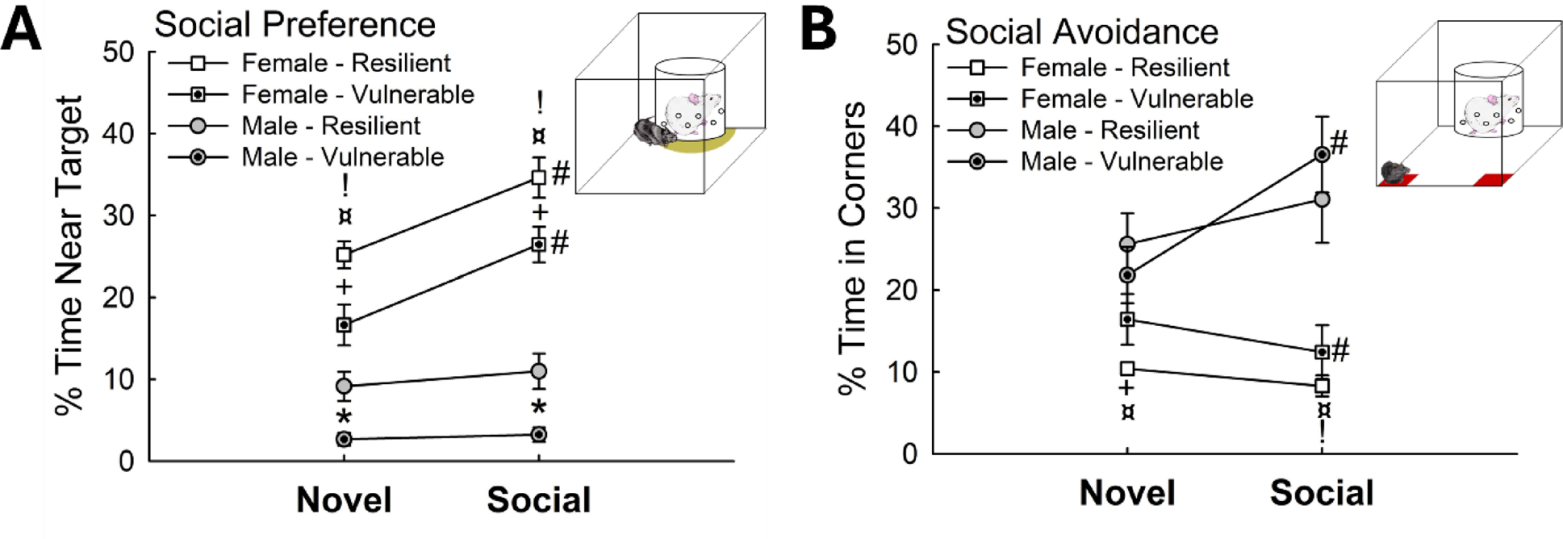
Sex and interaction dissociable social preference responses indicate distinct female stress-vulnerable and resilient phenotypes. **(A)** After 4-Day SAM exposure, female mice display higher preference for both novel and social targets in the SIP test compared to male mice of Escape and Stay phenotypes (N = 84; Phenotype Effect: F_1,81_ = 18.5, *p*< 0.001; Sex Effect: F_1,81_ = 74.1, *p* < 0.001; Novel, Resilient Female (low corticosterone) vs Male (Escape), t_38_ = 5.6, ^¤^*p* < 0.001; Novel, Vulnerable Female (high corticosterone vs Male (Stay), t_43_ = 7.1, ^!^*p*< 0.001; Novel, Male – Resilient vs Male – Vulnerable, t_61_ = 3.6,*p < 0.001; Social, Female vs Male – Resilient, t_38_ = 6.6,^¤^*p* < 0.001; Social, Female vs Male – Vulnerable, t_43_ = 11.2, ^!^*p*< 0.001; Social, Male – Resilient vs Male – Vulnerable, t_61_ = 3.6, *p < 0.001; Novel vs Social, Female – Resilient, t_12_= 3.5, #*p* < 0.001; Female – Vulnerable, t_8_ = 5.1, ^#^*p*< 0.001; Novel, Female – Resilient vs Female – Vulnerable, t_20_= 2.9, ^+^*p *≤ 0.008; Social, Female – Resilient vs Female – Vulnerable, t_20_ = 2.3, ^+^*p *≤ 0.030). **(B)** Female mice also show reduced social aversion behavior after SAM interactions (N = 75; Novel – Sex Effect: F_1,72_ = 4.5, *p* ≤ 0.037; Novel, Female vs Male– Resilient, t_38_ = 2.7, ^¤^*p* ≤ 0.009; Female – Resilient vs Female – Vulnerable, t_20_ = 2.2, ^+^*p*≤ 0.036; Social – Sex Effect: t_1,72_ = 12.5, *p *< 0.001; Female – Resilient vs Male – Resilient, t_38_ = 2.9, ^¤^*p *≤ 0.006; Female – Vulnerable vs Male – Vulnerable, t_43_ = 2.5, ^!^*p* ≤ 0.015; Novel vs Social, Female – Vulnerable, t_8_ = 2.9, #*p* ≤ 0.017; Male – Vulnerable, Novel vs Social, t_35_ = 3.0, #*p* < 0.005)

**Fig. 5:**
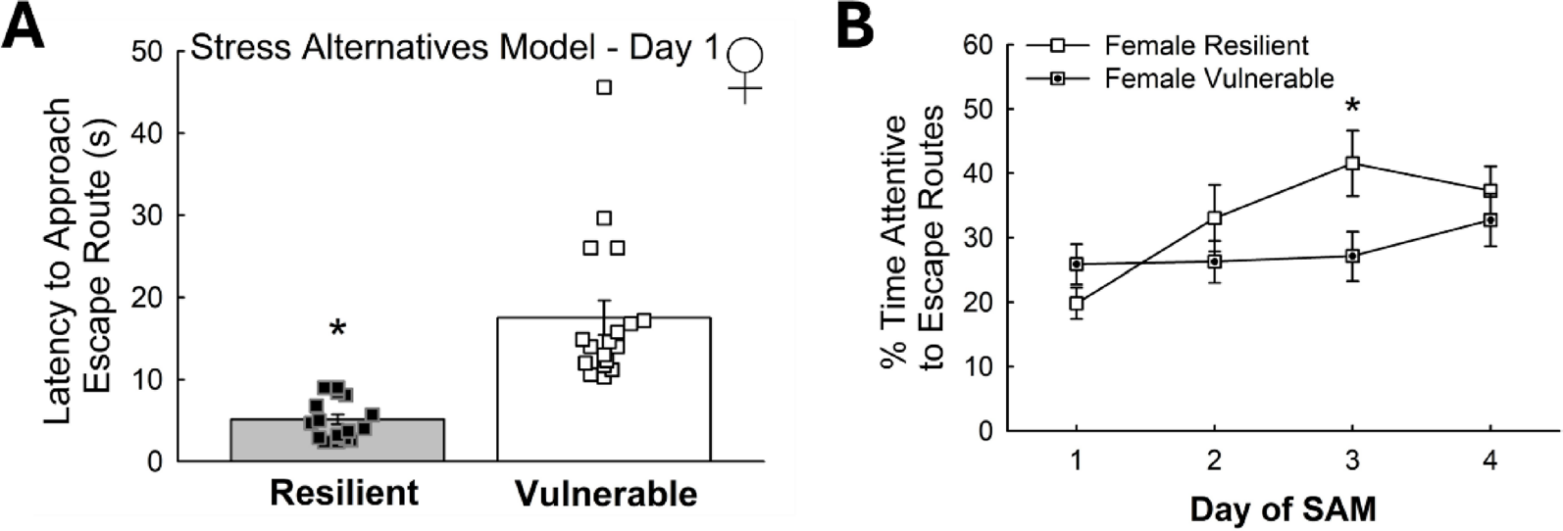
Motivation to escape differs by phenotype. **(A)** Latency to approach the escape route were also distinguishable in females resilient (N = 15) and vulnerable (N = 19) phenotypic groups. The difference in latency of females to approach the escape route during the standard 4-Day SAM is significantly different on Day 1 (N = 36, t_34_ = 5.6, *p < 0.001). **(B)** Resilient females spend more time exploring the escape routes over the days (Day Effect: F_3,90_= 3.6, *p *≤ 0.016; Female Resilient – F_3,36_= 7.2, ^#^*p* < 0.001), especially on Day 3 compared to vulnerable counterparts (t_20_ = 2.1, *p ≤ 0.05)

### Stereotaxic surgery

Mice were anesthetized using isoflurane (2% at 1.0 L/min flow rate) to allow for bilateral intra-BLA (iBLA) guide cannula (PlasticsOne, Roanoke, VA; 26 ga cut to 4.0 mm) implantation, then given 7 days for recovery before behavioral testing. Bilateral iBLA cannulae placements were performed using the stereotaxic coordinates: −1.35 mm AP, ± 3.30 ML, and − 4.90 mm DV. During and after (30 min) surgery, mice were placed on a warming pad to maintain core body temperature. Post-surgery recovery and monitoring occurred in the home cage environment. Mice were injected subcutaneously with analgesic ketorolac (5 mg/kg) immediately following surgery and 24 h after receiving the first injection for a total of 48 h post-surgery pain relief. Following behavioral experiments, brain tissue was dissected, fixed and frozen for later sectioning and identification of cannulae end and correct injection placement directed at BLA (Fig. S4). One vehicle-treated animal was excluded from analysis with bilateral injections that missed BLA (Fig. S4).

### Drugs

For pharmacological experiments, an Orx_2_R antagonist (MK-1064; IC_50_ = 18 nM for Orx_2_R; 30 and 300 nmol, plus 1 µmol; MedChemExpress, Monmouth Junction, NJ), an α_2_ adrenoreceptor antagonist (yohimbine; 5 mg/kg), and vehicle (1:3, Saline to DMSO ratio) were delivered subcutaneously (sc) on Day 3. Additionally, bilateral intra-basolateral amygdalar (iBLA) injections of MK-1064 were delivered on Day 3 (Figs. S1, S2) in a volume of 0.3 µl, at doses of 6, 15, 30, or 60 fmol. A dose response determined the systemic dose of MK-1064 to use in female mice, with the requirement that the highest dosage (1 µmol; 60 fmol for iBLA studies) be less than the amount necessary to induce sleep/reduced locomotion in mice [60]. For our pro-anxiogenic control group, a dose of 5 mg/kg yohimbine (Sigma-Aldrich, Burlington, MA) (blocked escape in male mice [32]), was used for females.

### Corticosterone concentrations

Plasma, kept frozen in a −80°C freezer, was assessed for corticosterone concentration. Samples in each experiment were quantified in duplicate using a single run by corticosterone enzyme linked immunosorbent assay kits (Enzo Life Sciences, Farmingdale, NY).

#### In situ hybridization - RNAscope

RNAscope is a highly selective and sensitive in situ hybridization technology. The design of RNAscope probes and subsequent amplifier steps allow for increased sensitivity and reduce the potential for RNA degradation. The fluorescent signal generated through these methods provides specificity at the single transcript level, which is visualized as distinct puncta. Sections of fresh frozen brains (coronal; 20 μm; relative to bregma AP −1.40 to −2.0) were placed in cold (4 °C) 10% formalin for 20 min and subsequently washed (2x for 1 min) in 1x phosphate buffer solution (PBS), before dehydration with ethanol (50% x 1, 70% x 1, and 100% x 3; 5 min each with the final ethanol being kept at −20 °C overnight). The following day, proteins were digested using a protease treatment and rinsed with dH_2_O. Brain sections were incubated for two hours in RNAscope (Advanced Cell Diagnostics, Newark, CA) probes (*Hcrtr2*, Cat. No. 460881; *Cck*, Cat. No. 402271; *Som*, Cat. No. 482691; *Adra2a*, Cat. No. 425348) in a hybridization oven (ACD HybEZ II oven) set to 40 °C. Fluorophores (TSA Vivid 520, 570, and 650, imaged at wavelengths of 488, 555, and 647 nm) were linked to probes and signaling was enhanced through application of a series of amplification buffers (RNAscope Fluorescent Multiplex Detection Reagents). Finally, tissue was briefly stained with DAPI (20 s) and covered. Image acquisition was performed fluorescence microscope (Nikon A1; 10x/0.30 Plan Fluor and 20x/0.75 Plan Apo VC Nikon objectives) and NIS Elements software. The BLA was identified from images and analyzed visually and quantitatively for right and left hemispheres separately, then averaged, using QuPath 3.0 and ImageJ programs. Analysis through QuPath identified and quantified transcripts for each RNAscope target, visualized as puncta, each representing an individual RNA transcript, which allows direct quantitative analysis [61]. Analyses with QuPath involved selecting a subcellular detection threshold that best captured the individual RNA puncta, and this threshold was used for assessment of all imaged sections, along with quantification of co-expression of DAPI with *Hcrtr2* and *Cck*, *Som* or *Adra2a*, within a single experiment. Quantification of mRNA targets were strictly localized to BLA regions (Bregma − 1.0 to −2.0). Puncta counts were normalized by total cell count (DAPI) in the BLA. Cage Controls are included as a completely untreated comparison for injection and treatments. Each graphical data point represents one animal (includes 4–6 BLA regions quantified and averaged per animal).

### Statistical analysis

Experimental design and statistical analyses were based on a priori hypotheses, for the purpose of avoiding combinatorial exponential expansion of error from multiple tests [62]. This statistical pre-planning allows for a wider range of multiple comparison analyses across hypothetical designs. A level of 0.05 was set as the limit for statistical significance. For comparisons that involved SAM (and other behavioral paradigm) trials across days, SIP test results for Novel and Social target, and Fear Response test (contextual and cued) analyses, we utilized two-way repeated measures ANOVA. For changes occurring across treatment/experimental groups we applied one-way ANOVA. While the statistics for behavior did not include cage controls; these non-stressed cage controls added for comparisons, and were necessary for interpretation, of corticosterone levels, in situ hybridization results, and home cage mobility measurements, in which one-way ANOVA was used. Assessments between two treatments/experimental conditions were performed by Student’s two-tailed t-tests. To determine differences in percentage of escape or estrous cycle stage, chi-square and Fischer Exact statistical analyses were utilized.

Each mouse provided a singular sample source, from which multiple measures and analyses were taken. Every unit for analyses involved a priori hypotheses. The five assumptions of parametric statistics were applied to the data (Random and Equal samples, Normal distribution of data [examined by the Kolmogorov-Smirnov test], Homogeneity of variance [similar homoscedasticity, examined by Levene’s test], Independence of data for different groups, Interval level data – linearity), which were transformed, when necessary, compared to non-parametric analyses, and graphed in raw form. Analyses for parametric and non-parametric statistics were used along with an examination for multiple comparisons applying the Holm-Sidak method. If the statistical analyses match, as they do for the data herein, we report the parametric results without α adjustment [63–68] based on a priori hypothesis driven exclusion from combinatorial effect [62]. Effects between groups for one-way analyses were examined with Student–Newman–Keuls post hoc analyses (to minimize Type I error) and Duncan’s Multiple Range Test (to minimize Type II error).

## Results

### Social stress prompts avoidance in female mice

To investigate the effects of social stress on female mice, we developed a method for producing male CD1 retired breeder mice that act aggressively toward female mice. As males investigated (including anogenital sniffing) female mice, a mild shock (0.04 mA, < 1 s) was applied to the rump region, which resulted in intense attacks of female conspecifics (classically conditioned aggression, CCA; Fig. 1A). The number of shocks necessary to promote aggression varied (average = 2.85), however, all males introduced to our protocol successfully exhibited aggression toward female mice (Fig. 1B).

The Stress Alternatives Model (SAM) is a 4-day social stress paradigm in which male mice diverge into distinguishable behavioral phenotypes, by actively avoiding (Escape) or accepting (Stay) confrontation (Figs. 1C, S1). Although the SAM has been used extensively in males [30, 45, 48, 52, 69, 70], it has not previously been applied to female mice. Surprisingly, all females subjected to the SAM chose to avoid social aggression by the end of Day 3, which is different from males, in which ~ 45% escape (*n* = 85, Day 1: *χ*^2^ = 18.6, *p* < 0.001; Day 2: *χ*^2^ = 16.3, *p* < 0.001; Day 3 & 4: *χ*^2^ = 19.5, *p* < 0.001; Fig. 1D). Nevertheless, manipulating the stress state of females by exposing them to brief bouts of social aggression for 4-days prior to SAM exposure reduced the percentage of Escape mice (*n* = 42, Day 1: χ^2^ = 4.9, *p ≤ 0.03; Day 2: χ^2^ = 3.2, *p ≤ 0.04; Fig. 1E). Thus, in female mice, additional social stress results in more expression of the Stay phenotype.

Escape latency of Escape phenotype males mimicked that of females (*n* = 49, Day Effect: F_3,141_ = 51.8, *p* < 0.001), except for Day 1 where male mice exhibited a longer latency (male vs. female, Day 1: t_47_ = 2.4, *p ≤ 0.02; Fig. 2A). Interestingly, females of the Prior Stress + 4-day SAM experimental conditions (Fig. S2), displayed longer latency to escape times on Days 1–3 (*n* = 42, Paradigm Effect: F_1,120_ = 12.9, *p* < 0.001; Interaction Effect: F_3,120_ = 2.3, *p* ≥ 0.085; Day Effect: F_3,120_ = 51.0, *p* < 0.001; Prior stress + SAM vs. SAM Day 1: t_40_ = 4.3, *p < 0.001; Day 2: t_40_ = 2.8, *p < 0.001; Day 3: t_40_ = 2.6, *p ≤ 0.01) while, the learning profile [43], as indicated by the curve of the plot, remained similar (Fig. 2B). Importantly, males of the Stay phenotype encountered greater levels of aggression in the SAM compared to both females and Escape males (*n* = 85, Phenotype Effect: F_2,192_ = 42.9, *^or**+**^*p* < 0.001; Interaction Effect: F_6,192_ = 1.5, *p* ≥ 0.175; Day Effect: F_3,192_ = 5.1, ^**¤**^*p* ≤ 0.002), but there was no difference in aggression received when comparing females and males that avoid social aggression (Fig. 2C). Further, stressing females prior to SAM exposure did not affect the total amount of aggression they received from aggressor males during SAM trials; however, females presented with an inescapable social stress environment using CCA experienced the greatest amount of aggression (*n* = 52, Paradigm Effect: F_2,147_ = 32.4, *^or**+**^*p* < 0.001; Day Effect: F_3,147_ = 1.2, *p* ≥ 0.295; Interaction Effect: F_6,147_ = 1.4, *p* ≥ 0.229; Fig. 2D), which resembles that of Stay male mice (Fig. 2D). Notably, differences in male and female size/weight did not influence ability to escape (Fig. S3).

**Fig. 6:**
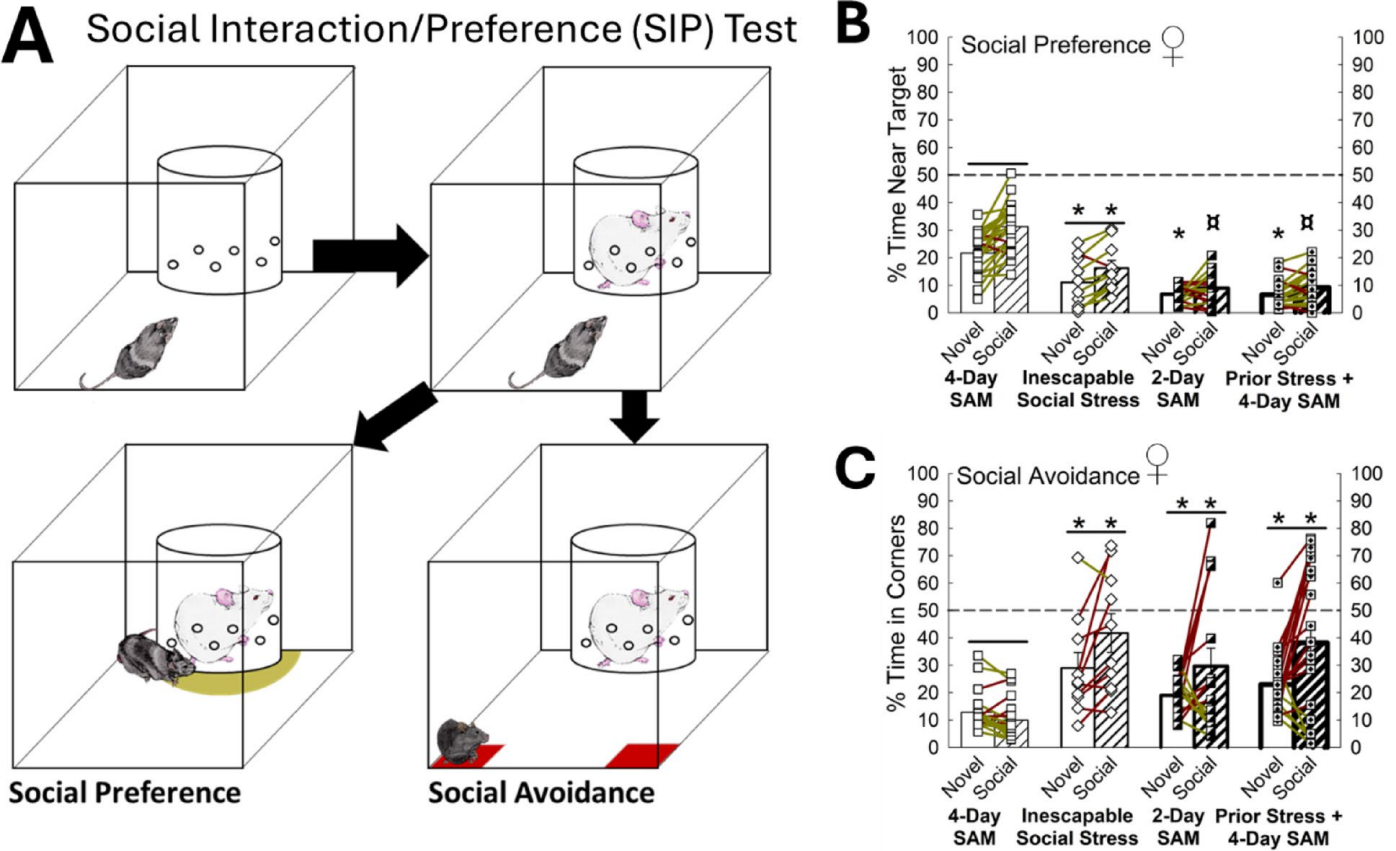
Social stress-promoting conditions foster social aversion in female mice. (**A**) Social Interaction/Preference (SIP) test measures social preference (bottom left) and aversion (bottom right). (**B**) Enhanced stress in females reduces exploration of both novel and social targets (N = 68, Social Stress Paradigm Effect: F_3,64_ = 41.3, *p* < 0.001; Target Effect: F_1,64_ = 37.6, *p* < 0.001; Interaction Effect: F_3,64_ = 5.8, *p* < 0.001; Novel, 4-Day SAM vs Inescapable Social Stress, t_30_ = 4.0, *p < 0.001; Novel-4-Day vs 2-Day SAM, t_36_ = 6.6, *p < 0.001; Novel, 4-Day SAM vs Prior Stress + 4-Day SAM, t_40_ = 7.1, *p < 0.001; Social, 4-Day SAM vs Inescapable Social Stress, t_30_ = 5.7, *p < 0.001; Social, 4-Day SAM vs 2-Day SAM, t_36_ = 9.8, *p < 0.001; Social, 4-Day SAM vs Social Stress + 4-Day SAM, t_40_ = 10.3, *p < 0.001; Social, Inescapable Social Stress vs 2-Day SAM, t_26_ = 2.6, ¤*p* ≤ 0.01; Social, Inescapable Social Stress vs Prior Stress + 4-Day SAM, t_28_ = 2.6, ¤*p* ≤ 0.01; 4-Day SAM, Novel vs Social, t_21_ = 7.1, _*p* < 0.001; Inescapable Social Stress, Novel vs Social, t_9_ = 2.6, *p* ≤ 0.01). (**C**) In females, social avoidance is increased in paradigms involving inescapable social stress, 2-Day SAM, and prior stress before 4-Day SAM (N = 68, Paradigm Effect: F_3,64_ = 9.4, *p* < 0.001; Target Effect: F_1,64_ = 12.2, *p* < 0.001; Interaction Effect: F_3,64_ = 3.3, *p* ≤ 0.03; Novel, 4-Day SAM vs Inescapable Social Stress, t_30_ = 3.7, *p < 0.001; Novel, 4-Day SAM vs 2-Day SAM, t_36_ = 2.7, *p ≤ 0.01; Novel, 4-Day SAM vs Prior Stress + 4-Day SAM, t_40_ = 3.5, *p < 0.001; Social, 4-Day SAM vs Inescapable Social Stress, t_30_ = 4.8, *p < 0.001; Social, 4-Day SAM vs 2-Day SAM, t_36_ = 3.4, *p < 0.001; Social, 4-Day SAM vs Social Stress + 4-Day SAM, t_40_ = 5.3, *p < 0.001; 4-Day SAM, Novel vs Social, t_21_ = 3.0, *p* ≤ 0.007; Inescapable Social Stress, Novel vs Social, t_9_ = 2.0, *p* ≤ 0.05; 2-Day SAM, Novel vs Social, t_15_ = 2.1, _*p* ≤ 0.04; Prior Stress + 4-Day SAM, Novel vs Social, t_19_ = 3.4, *p* < 0.001)

### Females exhibit elevated stress hormone levels

As male mice exposed to the SAM paradigm display elevated plasma corticosterone levels relative to unstressed mice [28, 29] we wanted to see if female mice exhibited the same physiological response (Fig. 3B, D). All female mice exposed to our various social stress paradigms (Fig. S1, S2) expressed higher plasma corticosterone levels [71, 72] relative to non-stressed controls (*n* = 82, F_4,77_ = 8.9, *p* < 0.001); however, there was no difference in stress hormone concentrations when comparing animals across experimental groups (Fig. 3D).

**Fig. 7:**
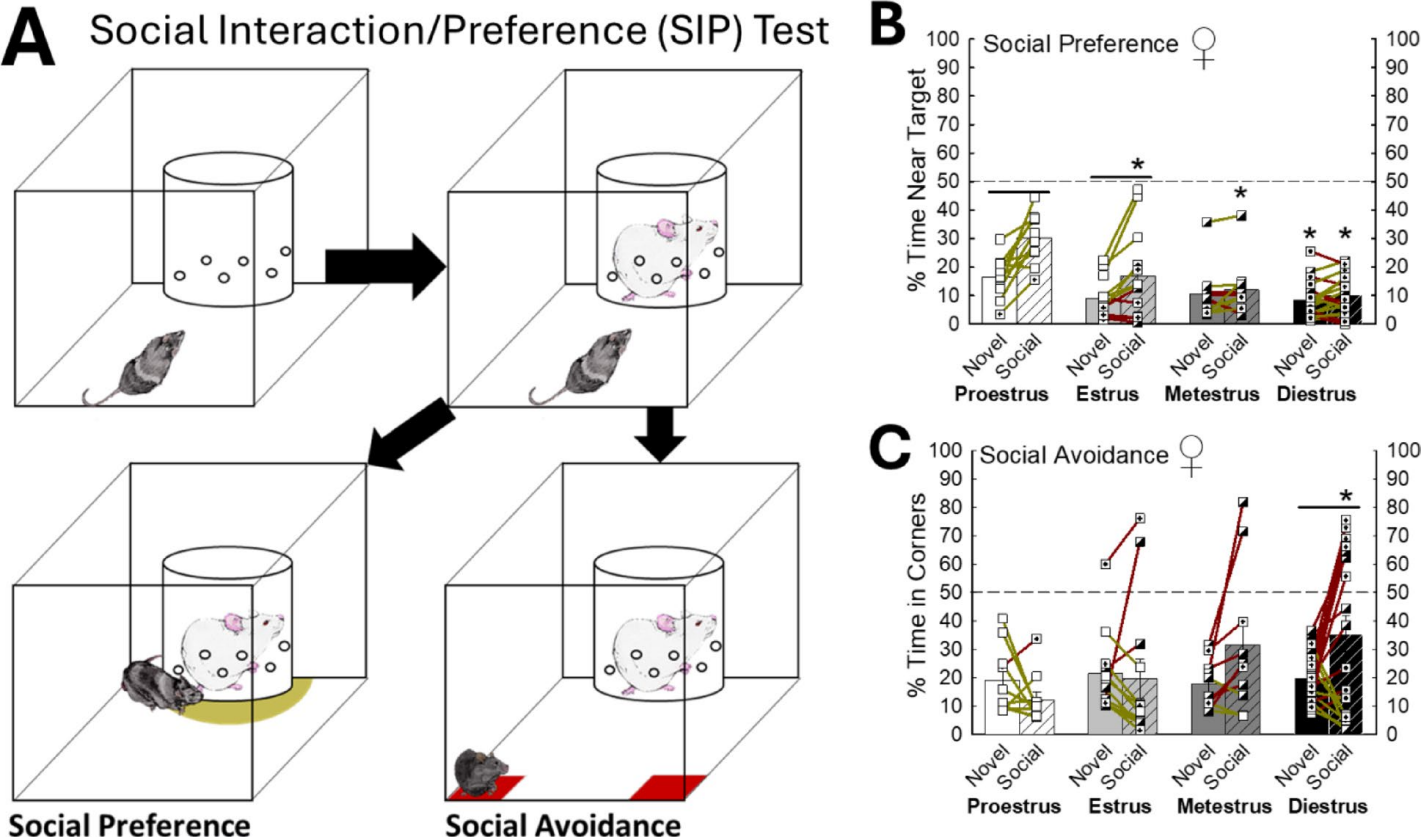
Female mice display preference during proestrus and avoidance during diestrus.** (A)** Social Interaction/Preference (SIP) test measures social preference (bottom left) and aversion (bottom right). **(B)** Mice in proestrus exhibit enhanced social preference compared to other stages of the estrous cycle (N = 53, Stage Effect: F_3,49_ = 6.7, *p* < 0.001; Target Effect: F_1,49_= 34.8, *p* < 0.001; Interaction Effect: F_3,49_ = 7.6, *p*< 0.001; Novel, Proestrus (N = 10) vs Diestrus (N = 20), t_28_ = 3.1, *p ≤ 0.005; Social, Proestrus vs Estrus (N = 13), t_21_ = 3.5, *p< 0.001; Social, Proestrus vs Metestrus (N = 10), t_18_ = 4.5, *p < 0.001; Social, Proestrus vs Diestrus, t_28_ = 5.7, *p< 0.001; Proestrus, Novel vs Social, t_9_ = 5.9, *p* < 0.001; Estrus (N = 13), Novel vs Social, t_12_ = 3.5, *p* < 0.001). **(C)** Animals in diestrus display higher social avoidance behavior (n = 53, Interaction Effect: F_3,49_ = 3.1, *p* ≤ 0.04; Social, Proestrus vs Diestrus, t_28_ = 3.1, *p ≤ 0.002; Diestrus, Novel vs Social, t_19_ = 3.0, *p* ≤ 0.004)

To further classify sex-tied distinctions in stress responsivity, we compared plasma corticosterone concentrations in male and female mice (Figs. 3D, E). While no differences were observed between male and female corticosterone concentrations in non-stressed mice (Fig. 3B), female mice exposed to social stress in the SAM displayed higher plasma corticosterone levels relative to male animals of both the Escape and Stay phenotype (*n* = 47, F_2,44_ = 6.9, *p* ≤ 0.003; Female vs. Male – Escape, t_30_ = 2.9, ^**+**^*p* ≤ 0.007; Female vs. Male – Stay, t_35_ = 2.3, +*p* ≤ 0.026; Male – Escape vs. Male – Stay, t_23_ = 2.4, *p ≤ 0.025; Fig. 3D). As demonstrated previously [28, 29], male mice of the Escape phenotype expressed lower plasma levels of corticosterone compared to Stay animals (*n* = 31, F_1,51_ = 36.6, *p* < 0.001; Male Resilient vs. Male Vulnerable, t_23_ = 2.4, *p ≤ 0.025; Fig. 3D, E). Closer examination revealed a distinct gap in the distribution of corticosterone levels suggesting a hormone-associated phenotype divergence in female mice, based on the high and low responder model, where high responder females presented elevated corticosterone measurements nearly twice the concentration as low responder females, and also male mice of the same stress state. These high corticosterone responder females were also determined to be stress vulnerable (Fig. 4A), as determined by the SIP test (Female Resilient vs. Female Vulnerable, t_28_ = 8.2, *p < 0.001; Female vs. Male – Resilient, t_30_ = 0.447, *p* ≥ 0.658; Female vs. Male – Vulnerable, t_21_ = 9.1, *p < 0.001; Fig. 3E). Together, these results suggest that social stress may expose sexually dimorphic behavior and physiology. As such, on Day 1 of SAM testing, two phenotypic groups arise in female mice, even though there was not a discernable difference in Escape/Stay behavior (Fig. 1E). These stress-vulnerable and resilient phenotypes were determined using the distribution of corticosterone data (Fig. 3C), from plasma taken after fear conditioning and SIP testing, and then compared to the fear response (CR, Fig. 3A) and significant SIP testing results (Figs. 4A, 5 and 6B).

**Fig. 8:**
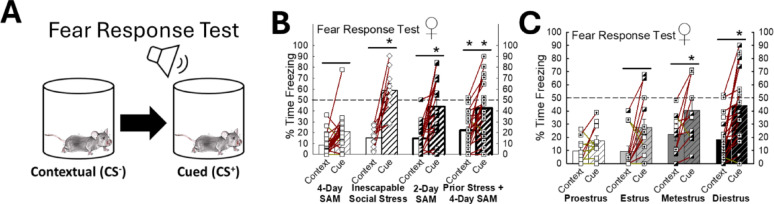
Social Stress promotes contextual and cued fear conditioning in females.** (A)** Fear Response test indicates if fear learning takes place and whether fear responses are observed in response to the context or cue. **(B)** In female mice, enhancing the stress state promotes increased freezing in the fear response test (N = 68, Paradigm Effect: F_3,64_ = 8.1, *p* < 0.001; Cued Effect: F_1,64_ = 87.6, *p* < 0.001; Interaction Effect: F_3,64_= 5.1, *p* ≤ 0.003; Contextual, 4-Day SAM (N = 22) vs Prior Stress + 4-Day SAM (N = 20), t_40_ = 3.4, *p ≤ 0.002; Cued, 4-Day SAM vs Inescapable Social Stress (N = 10), t_30_ = 5.5, *p < 0.001; Cued, 4-Day SAM vs 2-Day SAM (N = 16), t_36_= 3.9, *p < 0.001; Cued, 4-Day SAM vs Prior Stress + 4-Day SAM, t_40_ = 3.9, *p < 0.001; 4-Day SAM, Contextual vs Cued, t_21_ = 2.6, ^_^*p* ≤ 0.01; Inescapable Social Stress, Contextual vs Cued, t_9_ = 6.2, *p* < 0.001; 2-Day SAM, Contextual vs Cued, t_15_ = 5.2, ^_^*p *< 0.001; Prior Stress + 4-Day SAM, Context vs Cued, t_19_ = 4.1, ^_^*p*< 0.001). **(C)** Female mice in metestrus and diestrus display elevated cued fear freezing behavior (N = 53, Stage Effect: F_3,49_ = 4.3, *p* ≤ 0.009; Cued Effect: F_1,49_ = 30.2, *p* < 0.001; Cued, Proestrus vs Metestrus, t_18_ = 2.7, *p ≤ 0.008; Cued, Proestrus (N = 10) vs Diestrus (N = 20), t_28_ = 3.7, *p < 0.001; Estrus (N = 13), Context vs Cued, t_12_ = 2.9, *p* ≤ 0.005; Metestrus (N = 10), Contextual vs Cued, t_9_ = 2.6, ^_^*p* ≤ 0.01; Diestrus, Contextual vs Cued, t_19_ = 5.2, *p *< 0.001)

### Stress responsive behavior is sex dependent

To determine if male and female mice exhibit distinct behavioral patterns during and after social stress, we compared high and low corticosterone responders (Fig. 3E) with responses in SIP and Fear Response tests following the standard 4-day SAM paradigm (Fig. 4) and interest in the escape routes during SAM interaction (Fig. 5). On Day 1 of SAM testing, two phenotypic groups arise in female mice, stress-vulnerable and resilient phenotypes, determined through a bimodal distribution corticosterone data using the model of high and low responders (Fig. 3E), which also display reduced novel object investigation and social preference (high corticosterone response – vulnerable) contrasted with enhanced investigation and social preference (low corticosterone responders – resilient). Typically, male mice of the Escape phenotype express higher social preference compared to Stay animals. In these experiments, Escape males displayed elevated exploration of novel and social conditions, while female mice exhibited the highest levels of novelty exploration and social preference (*n* = 84; Phenotype Effect: F_1,81_ = 18.5, *p* < 0.001; Sex Effect: F_1,81_ = 74.1, *p* < 0.001; Novel exploration, Female vs. Male – Resilient, t_38_ = 5.6, ^**¤**^*p* < 0.001; Novel, Female vs. Male – Vulnerable, t_43_ = 7.1, ^**!**^*p* < 0.001; Novel, Male – Resilient vs. Male – Vulnerable, t_61_ = 3.6, *p < 0.001; Social preference, Female vs. Male – Resilient, t_38_ = 6.6, ^**¤**^*p* < 0.001; Social, Female vs. Male – Vulnerable, t_43_ = 11.2, ^**!**^*p* < 0.001; Social, Male – Resilient vs. Male – Vulnerable, t_61_ = 3.6, *p < 0.001; Novel vs. Social, Female – Resilient, t_12_ = 3.5, ^**#**^*p* < 0.001; Female – Vulnerable, t_8_ = 5.1, ^#^*p* < 0.001; Novel, Female – Resilient vs. Female – Vulnerable, t_20_ = 2.9, ^**+**^*p* ≤ 0.008; Social, Female – Resilient vs. Female – Vulnerable, t_20_ = 2.3, ^**+**^*p* ≤ 0.030; Fig. 4A).

Similarly, females expressed lower amounts of social aversion behavior compared to both Escape and Stay male mice (*n* = 75; Aversion – Sex Effect: F_1,72_ = 4.5, *p* ≤ 0.037; Social – Sex difference: t_1,72_ = 12.5, *p* < 0.001; Female – Resilient vs. Male – Resilient, t_38_ = 2.9, ^**¤**^*p* ≤ 0.006; Female – Vulnerable vs. Male – Vulnerable, t_43_ = 2.5, ^!^*p* ≤ 0.015; Novelty avoidance, Female vs. Male – Resilient, t_38_ = 2.7, ^**¤**^*p* ≤ 0.009; Female – Resilient vs. Female – Vulnerable, t_20_ = 2.2, ^+^*p* ≤ 0.036; Novel vs. Social, Female – Vulnerable, t_8_ = 2.9, #*p* ≤ 0.017; Male – Vulnerable, Novel vs. Social, t_35_ = 3.0, ^**#**^*p* < 0.005; Fig. 4B). Male mice of the Stay phenotype further displayed enhanced contextual and cued fear freezing behavior relative to both Escape male and female mice (*n* = 97; Contextual, Phenotype Effect: F_1,94_ = 10.3, *p* ≤ 0.002; Sex Effect: F_1,94_ = 7.0, *p* ≤ 0.010; Female vs. Male – Vulnerable, t_53_ = 1.9, ^**!**^*p* ≤ 0.05; Male – Resilient vs. Male – Vulnerable, t_74_ = 4.1, *p < 0.001; Female vs. Male – Resilient, t_41_ = 2.4, ^**¤**^*p* ≤ 0.023; Female vs. Male – Vulnerable, t_53_ = 1.9, ^**!**^*p* ≤ 0.05; Female – Resilient vs. Female – Vulnerable, t_20_ = 2.6, ^***+***^*p* ≤ 0.019). Male Escape animals also exhibited enhanced cued fear freezing compared to females (Cued, Sex Effect: F_1,94_ = 13.4, *p* < 0.001; Male – Resilient vs. Male – Vulnerable, t_74_ = 2.9, *p ≤ 0.004; Male – Resilient vs. Female – Resilient, t_41_ = 2.4, ^**¤**^*p* ≤ 0.020; Male – Vulnerable vs. Female – Vulnerable, t_53_ = 2.9, ^**!**^*p* ≤ 0.006; Fig. 4A). Importantly, both phenotypes from male mice as well as female mice showed cued fear learning, characterized by elevated freezing post-tone compared to pre-tone (Contextual vs. Cued, Male – Resilient, t_29_ = 5.8, ^**#**^*p* < 0.001; Male – Vulnerable, t_45_ = 7.3, ^**#**^*p* < 0.001; Female – Resilient, t_12_ = 5.9, ^**#**^*p* < 0.001; Fig. 4A). Additionally, these SIP-confirmed Vulnerable and Resilient Phenotypes also respond with slow or quick latencies to approach the escape route, respectively (*n* = 36, t_34_ = 5.6, *p < 0.001; Fig. 5A). Additionally, over days of the SAM, Resilient females spend significantly more time exploring the escape route (Day Effect: F_3,90_ = 3.6, *p ≤ 0.016; Resilient Female over time: F_3,36_ = 7.2, ^*^p < 0.001), especially on Day 3, compared to Vulnerable females (t_20_ = 2.1, *p ≤ 0.05; Fig. 5B).

### Female social preference/aversion is altered by stress responsive state

Given that male mice exhibiting the Escape phenotype show more social preference and less aversion in the Social Interaction/Preference (SIP) test [29], we examined the behavior of female mice subjected to various male: female social defeat (CCA) paradigms (Fig. S2) in this context (Fig. 2A). While female mice exposed to the standard 4-day SAM paradigm (Fig. S2) exhibited social preference (*see later in results for* Fig. 4A) and reduced social aversion (Fig. 4B), females subjected to inescapable social stress, a 2-day SAM paradigm, or prior stress before 4-day SAM exposure displayed reduced social exploration compared to the standard 4-Day SAM (*n* = 68, Paradigm Effect: F_3,64_ = 41.3, *p* < 0.001; Target Effect: F_1,64_ = 37.6, *p* < 0.001; Interaction Effect: F_3,64_ = 5.8, *p* < 0.001; Novel, 4-Day SAM vs. Inescapable Social Stress, t_30_ = 4.0, *p < 0.001; Novel, 4-Day SAM vs. 2-Day SAM, t_36_ = 6.6, *p < 0.001; Novel, 4-Day SAM vs. Prior Stress + 4-Day SAM, t_40_ = 7.1, *p < 0.001; Social, 4-Day SAM vs. Inescapable Social Stress, t_30_ = 5.7, *p < 0.001; Social, 4-Day SAM vs. 2-Day SAM, t_36_ = 9.8, *p < 0.001; Social, 4-Day SAM vs. Social Stress + 4-Day SAM, t_40_ = 10.3, *p < 0.001; Social, Inescapable Social Stress vs. 2-Day SAM, t_26_ = 2.6, ^**¤**^*p* ≤ 0.01; Social, Inescapable Social Stress vs. Prior Stress + 4-Day SAM, t_28_ = 2.6, ^**¤**^*p* ≤ 0.01; 4-Day SAM, Novel vs. Social, t_21_ = 7.1, *p* < 0.001; Inescapable Social Stress, Novel vs. Social, t_9_ = 2.6, *p* ≤ 0.01; Fig. 6A) and enhanced social aversion, also compared to the standard 4-Day SAM (*n* = 68, Paradigm Effect: F_3,64_ = 9.4, *p* < 0.001; Target Effect: F_1,64_ = 12.2, *p* < 0.001; Interaction Effect: F_3,64_ = 3.3, *p* ≤ 0.03; Novel, 4-Day SAM vs. Inescapable Social Stress, t_30_ = 3.7, *p < 0.001; Novel, 4-Day SAM vs. 2-Day SAM, t_36_ = 2.7, *p ≤ 0.01; Novel, 4-Day SAM vs. Prior Stress + 4-Day SAM, t_40_ = 3.5, *p < 0.001; Social, 4-Day SAM vs. Inescapable Social Stress, t_30_ = 4.8, *p < 0.001; Social, 4-Day SAM vs. 2-Day SAM, t_36_ = 3.4, *p < 0.001; Social, 4-Day SAM vs. Social Stress + 4-Day SAM, t_40_ = 5.3, *p < 0.001; 4-Day SAM, Novel vs. Social, t_21_ = 3.0, ^_^*p* ≤ 0.007; Inescapable Social Stress, Novel vs. Social, t_9_ = 2.0, ^_^*p* ≤ 0.05; 2-Day SAM, Novel vs. Social, t_15_ = 2.1, ^_^*p* ≤ 0.04; Prior Stress + 4-Day SAM, Novel vs. Social, t_19_ = 3.4, ^_^*p* < 0.001; Fig. 6B). These results suggest that for females, like males, the stress state defines social behavior in the SIP test.

As hormonal changes associated with the estrous cycle may further modify stress-related behaviors [73], we investigated whether behaviors exhibited in the SIP test were related to the stage of estrous (Figs. 7B, C, S5-8). Mice in proestrus exhibited the highest amount of social preference (*n* = 53, Stage Effect: F_3,49_ = 6.7, *p* < 0.001; Target Effect: F_1,49_ = 34.8, *p* < 0.001; Interaction Effect: F_3,49_ = 7.6, *p* < 0.001; Novel exploration, Proestrus vs. Diestrus, t_28_ = 3.1, *p ≤ 0.005; Social preference, Proestrus vs. Estrus, t_21_ = 3.5, *p < 0.001; Social, Proestrus vs. Metestrus, t_18_ = 4.5, *p < 0.001; Social, Proestrus vs. Diestrus, t_28_ = 5.7, *p < 0.001; Proestrus, Novel vs. Social, t_9_ = 5.9, *p* < 0.001; Estrus, Novel vs. Social, t_12_ = 3.5, *p* < 0.001; Fig. 7B), while animals in diestrus showed the greatest amount of social aversion (*n* = 53, Interaction Effect: F_3,49_ = 3.1, *p* ≤ 0.04; Social aversion, Proestrus vs. Diestrus, t_28_ = 3.1, *p ≤ 0.002; Diestrus, Novel aversion vs. Social aversion, t_19_ = 3.0, ^_^*p* ≤ 0.004; Fig. 7C). Importantly, stress state has been demonstrated to shift the hormonal balance and therefore the stage of estrous [74], but SAM social stress has not generally had that effect in these experiments (Figs. S5-8).

### Female stress state defines fear response

Female mice presented with our various stress paradigms (Fig. S2) all displayed cued fear learning (Fig. 8A, B); however, mice introduced to the experiments involving inescapable social stress, 2-day SAM, and prior stress before 4-day SAM demonstrated increased cued fear relative to animals from the standard 4-day SAM (*n* = 68, Paradigm Effect: F_3,64_ = 8.1, *p* < 0.001; Cued Effect: F_1,64_ = 87.6, *p* < 0.001; Interaction Effect: F_3,64_ = 5.1, *p* ≤ 0.003; Cued, 4-Day SAM vs. Inescapable Social Stress, t_30_ = 5.5, *p < 0.001; Cued, 4-Day SAM vs. 2-Day SAM, t_36_ = 3.9, *p < 0.001; Cued, 4-Day SAM vs. Prior Stress + 4-Day SAM, t_40_ = 3.9, *p < 0.001; 4-Day SAM, Contextual vs. Cued, t_21_ = 2.6, ^_^*p* ≤ 0.01; Inescapable Social Stress, Contextual vs. Cued, t_9_ = 6.2, *p* < 0.001; 2-Day SAM, Contextual vs. Cued, t_15_ = 5.2, *p* < 0.001; Prior Stress + 4-Day SAM, Contextual vs. Cued, t_19_ = 4.1, *p* < 0.001; Fig. 8B). Further, those mice in the Prior Stress + 4-day SAM experimental group also displayed enhanced contextual fear freezing compared to the 4-Day SAM group (Interaction Effect: F_3,64_ = 5.1, *p* ≤ 0.003; Contextual, 4-Day SAM vs. Prior Stress + 4-Day SAM, t_40_ = 3.4, *p ≤ 0.002; Fig. 8B). Interestingly, mice in proestrus did not display fear learning and exhibited reduced cued freezing relative to animals in metestrus and diestrus (*n* = 53, Estrous Stage Effect: F_3,49_ = 4.3, *p* ≤ 0.009; Cued Effect: F_1,49_ = 30.2, *p* < 0.001; Cued, Proestrus vs. Metestrus, t_18_ = 2.7, *p ≤ 0.008; Cued, Proestrus vs. Diestrus, t_28_ = 3.7, *p < 0.001; Estrus, Contextual vs. Cued, t_12_ = 2.9, ^_^*p* ≤ 0.005; Metestrus, Contextual vs. Cued, t_9_ = 2.6, ^**_**^*p* ≤ 0.01; Diestrus, Contextual vs. Cued, t_19_ = 5.2, ^**_**^*p* < 0.001; Fig. 8C).

### BLA ***Hcrtr2-***containing cells are expressed equally in males and females

**Fig. 9:**
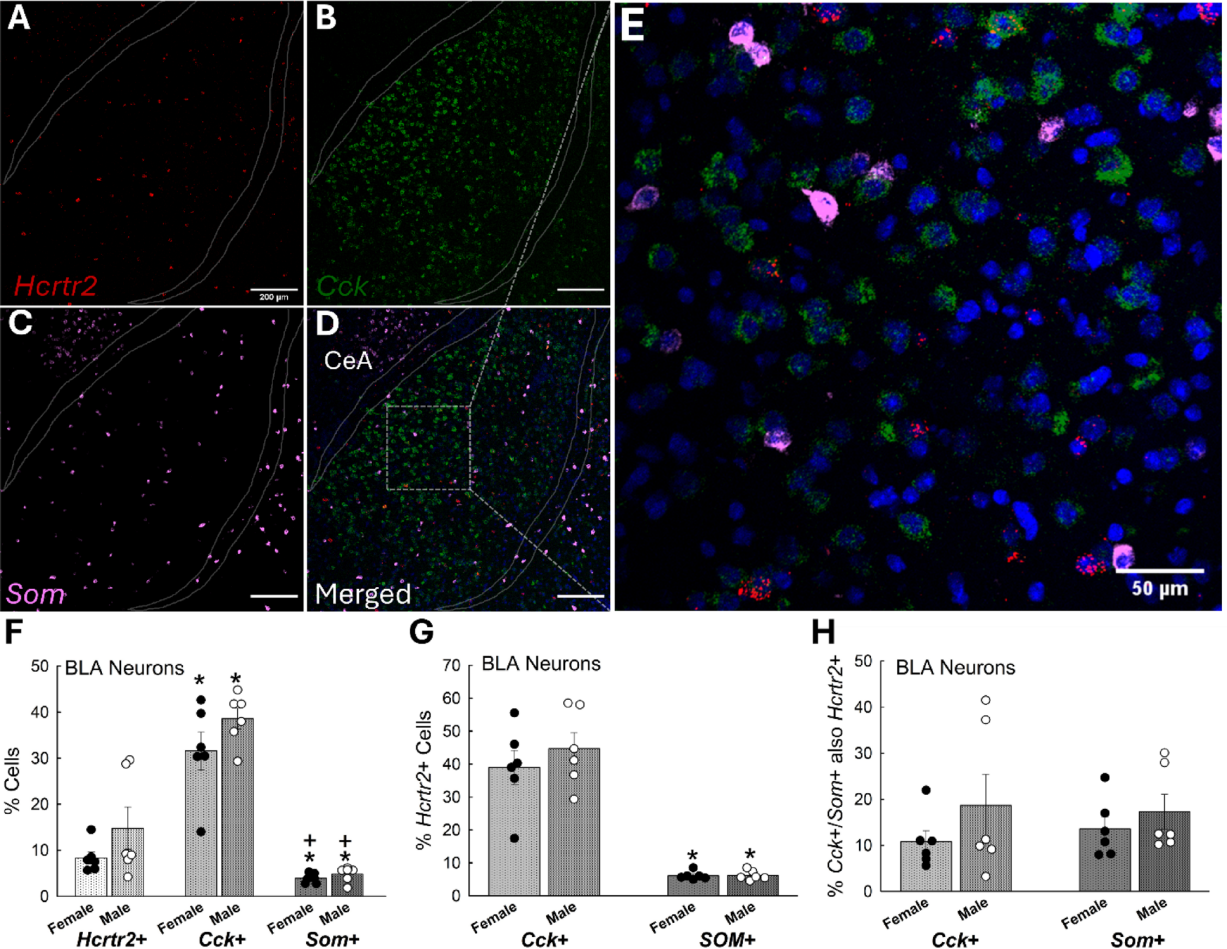
In the BLA of both females and males, Orx_2_R is highly co-expressed with cholecystokinin-expressing cells. Targeting **(A)** Orx_2_R (*Hcrtr2*), **(B)** cholecystokinin (*Cck*), and **(C)** somatostatin (*Som*) in **(D** & **E)** BLA tissue (DAPI staining in blue) revealed **(F),** few cells (~5%) expressing *Som*, a greater amount (~10%) expressing *Hcrtr2*, and many more expressing *Cck* in both female and male mice (N = 12, Sex Effect: F_1,30_ = 4.5, *p*≤ 0.042; Cell Type Effect: F_2,30_ = 68.1, *p* < 0.001; Female, *Hcrtr2*^+^ vs *Cck*^+^, t_10_ = 6.0, *p < 0.001; Female, *Cck*^+^ vs *Som*^+^, t_10_ = 7.1, ^+^*p* < 0.001; Male, *Hcrtr2*^+^ vs *Cck*^+^, t_10_ = 6.1, *p ≤ 0.025; Male, *Cck*^+^ vs *Som*^+^, t_10_ = 8.7, ^+^*p* ≤ 0.017). **(G)** In both sexes, more *Hcrtr2* is expressed in cells also expressing *Cck* than in *Som* neurons (N = 12, *Hcrtr2*^+^ Effect, F_1,20_= 102.1, *p* < 0.001; Female, *Cck*^+^ vs *Som*^+^, t_10_ = 6.6, *p < 0.001; Male, *Cck*^+^ vs *Som*^+^, t_10_ = 7.7, *p < 0.001); however, **(H)** proportionally, *Hcrtr2* is expressed in *Cck* and *Som* cells similarly

In cells of the BLA, Orx_2_R are expressed at low levels in male mice [29], which are predominantly found in GABAergic neurons [27]. Male and female mice expressed similar levels of Orx_2_R mRNA (*Hcrtr2*) in the BLA (*Cck*; *n* = 12, Sex Effect: F_1,30_ = 4.5, *p* ≤ 0.042; Cell Type Effect: F_2,30_ = 68.1, *p* < 0.001; Female, *Hcrtr2*^+^ vs. *Cck*^+^, t_10_ = 6.0, *p < 0.001; Male, *Hcrtr2*^+^ vs. *Cck*^+^, t_10_ = 6.1, *p ≤ 0.025; *Som;* Female, *Cck*^+^ vs. *Som*^+^, t_10_ = 7.1, ^**+**^*p* < 0.001; Male, *Cck*^+^ vs. *Som*^**+**^, t_10_ = 8.7, ^**+**^*p* ≤ 0.017; Figs. 9A-F), with most cells expressing *Hcrtr2* co-localization with cholecystokinin and not somatostatin (*n* = 12, *Hcrtr2*^+^ Effect, F_1,20_ = 102.1, *p* < 0.001; Female, *Cck*^+^ vs. *Som*^+^, t_10_ = 6.6, *p < 0.001; Male, *Cck*^+^ vs. *Som*^+^, t_10_ = 7.7, *p < 0.001) neurons (Figs. 9D-E, G). Importantly, the proportion of *Cck*/*Som* cells that also express *Hcrtr2* is approximately equal in both males and females (Fig. 9H) despite there being more overall cells with *Cck*, or *Cck* combined with *Hcrtr2* [75].

### Antagonism of Orx_2_R exposes stress-induced phenotypes in female mice

**Fig. 10:**
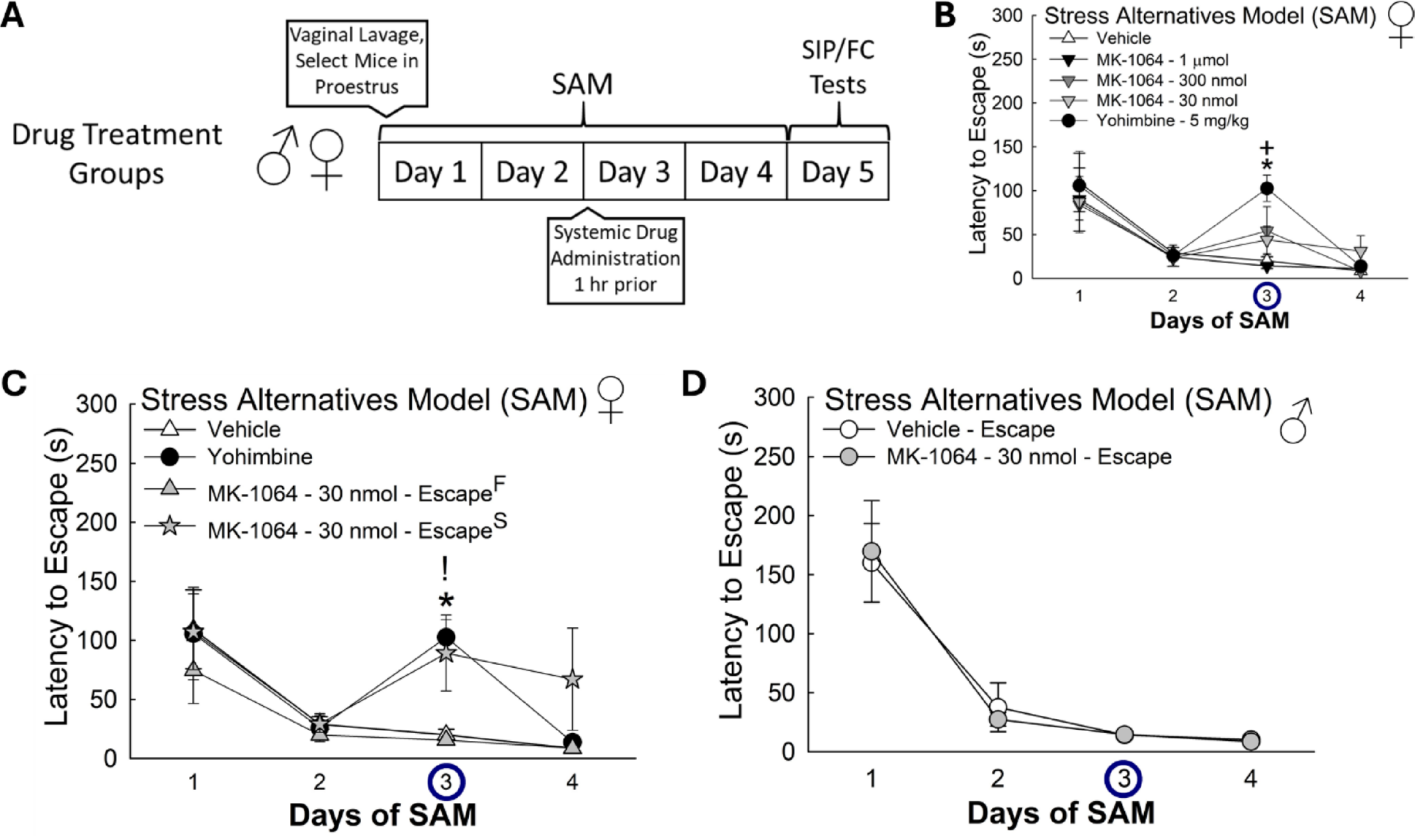
Systemic antagonism of Orx_2_R in female mice triggers phenotype formation. (**A**) Experimental design for pharmacological experiments. (**B**) Yohimbine, an α_2_ receptor antagonist, significantly increases latency to escape, while low doses of an Orx_2_R antagonist (MK-1064) moderately alter latency to escape (N = 41, Day Effect: F_3,108_ = 17.2, *p*< 0.001; Day 3, Vehicle vs Yohimbine, t_11_ = 4.9, *p < 0.001; Day 3, MK-1064 – 1 µmol vs Yohimbine, t_12_ = 5.8, ^+^*p* < 0.001). (**C**) Phenotype separation in female mice after low dose (30 nmol) treatment of an Orx_2_R antagonist (MK-1064) reveals fast (Escape^F^) and slow (Escape^S^) escapers, where Escape^S^ animals express an enhanced latency to escape comparable to that observed after yohimbine treatment (N = 26, Treatment Effect: F_3,66_ = 3.9, *p* ≤ 0.02; Day Effect: F_3,66_ = 11.0, *p*< 0.001; Day 3, Vehicle vs MK-1064 – 30 nmol – Escape^S^, t_9_= 2.6, *p ≤ 0.03; Day 3, MK-1064 – 30 nmol – Escape^F^ vs MK-1064 – 30 nmol – Escape^S^, t_11_= 3.0, ^!^*p* ≤ 0.01). (**D**) Male Escape mice administered MK-1064 at 30 nmol did not display increased latency to escape

**Fig. 11:**
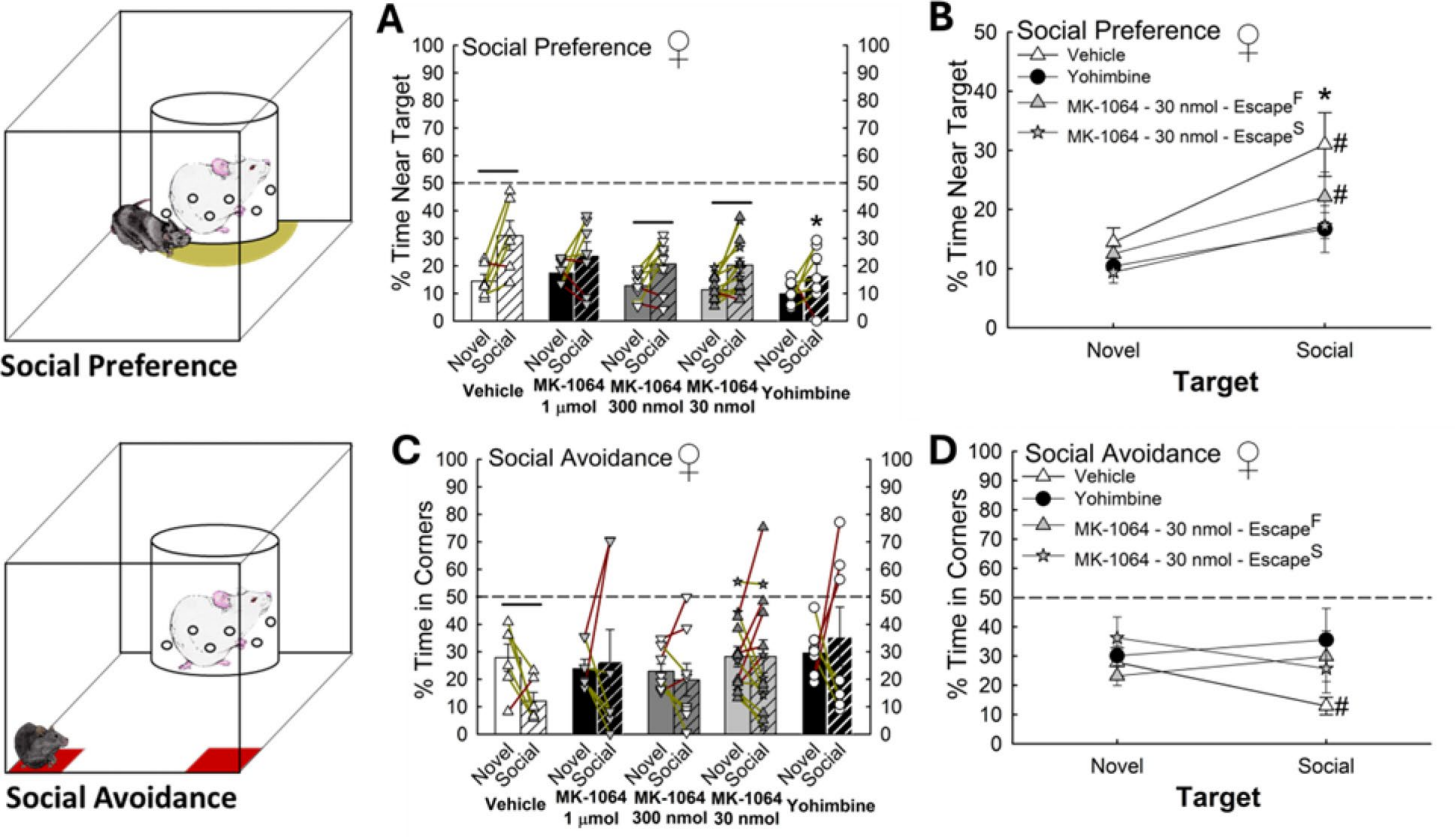
Anxiogenic treatments modify social preference in females. **(A)** At the 1 µmol subcutaneous Orx_2_ antagonist MK-1064 dose, social preference is lost, similar to 5 ng/kg α_2_ adrenoreceptor antagonist yohimbine (N = 41, Target Effect: F_1,36_ = 27.8, *p* < 0.001; Vehicle (N =6)-Novel vs Social, t_5_ = 3.8, *p*< 0.001; 300 nmol (N = 8)-Novel vs Social, t_7_ = 2.1, *p* ≤ 0.045; 30 nmol (N = 13)-Novel vs Social, t_12_ = 3.0, *p* ≤ 0.005). Yohimbine also reduced total social interaction time (Social-Vehicle vs Yohimbine (N = 7), t_11_ = 3.1, *p≤ 0.003). **(B)** Female Escape^S^ (slow escape (N = 5)) mice treated with 30 nmol MK-1064 displayed reduced social preference compared to vehicle treated mice, and comparable to yohimbine-treated mice (N = 26, Target Effect: F_1,16_= 24.1, *p* < 0.001; Social-Vehicle vs MK-1064–30 nmol–Escape^S^, t_9_ = 2.7, *p ≤ 0.01; MK-1064–30 nmol–Escape^F^(N= 8), Novel vs Social, t_7_ = 2.8, ^#^*p* ≤ 0.01). **(C, D)** Vehicle-treated mice exhibited less social aversion than novel exploration (t_5_ = 2.5, ^#^p ≤ 0.05)

**Fig. 12:**
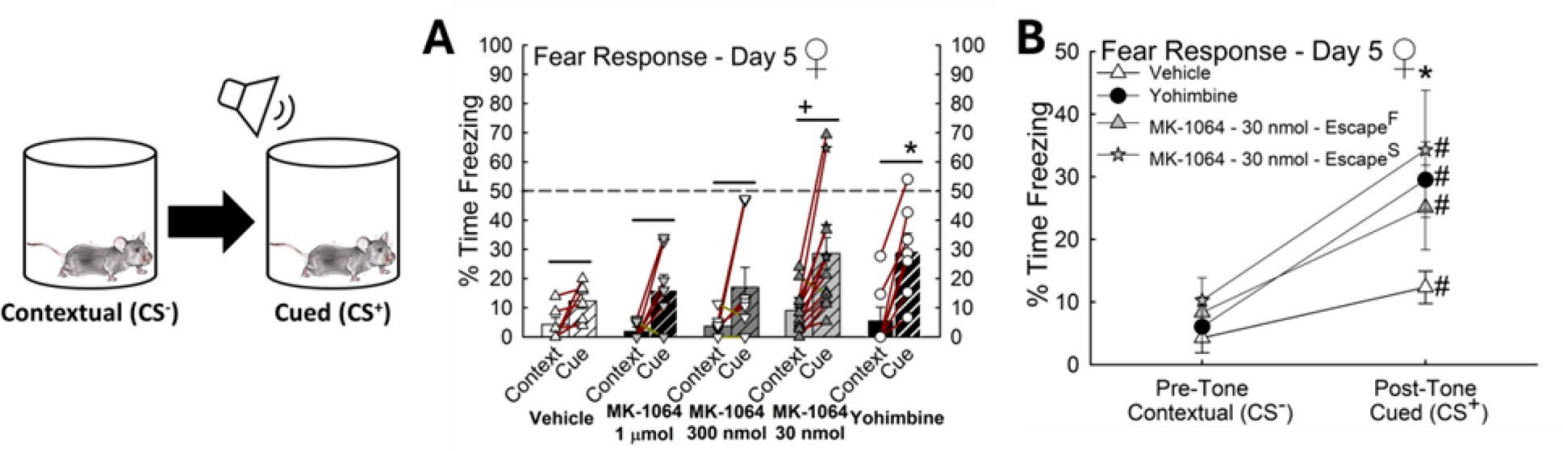
Anxiogenic treatments modify fear conditioning in females. **(A)** Treatment with 30 nmol dose of the Orx_2_R antagonist increased contextual fear conditioning, whereas 5 ng/kg α_2_ adrenoreceptor antagonist of the yohimbine increased cued fear freezing in females (N = 41, Conditioning Effect: F_1,36_ = 42.0, *p* < 0.001; Cued, MK-1064–1 µmol (N = 7) vs MK-1064– 30nmol (N = 13), t_18_ = 2.4, ^+^*p* ≤ 0.03; Cued, Vehicle (N = 7) vs Yohimbine (N = 7), t_11_ = 2.5, *p ≤ 0.03; Vehicle, Contextual vs Cued, t_5_ = 2.5, *p* ≤ 0.05; MK-1064 – 1 µmol, Contextual vs Cued, t_6_ = 2.4, *p* ≤ 0.02; MK-1064 – 300 nmol, Contextual vs Cued, t_7_ = 2.5, *p* ≤ 0.02; MK-1064 – 30 nmol, Contextual vs Cued, t_12_ = 4.7, *p* < 0.001; Yohimbine, Contextual vs Cued, t_6_ = 4.2, *p* < 0.001). **(B)** Female Escape^S^ phenotype (N = 5) displays elevated cued freezing similar to yohimbine-treated female mice (N = 26, Conditioning Effect: F_3,22_ = 44.7, *p*< 0.001; Cued, Vehicle vs MK-1064–30 nmol–Escape^S^, t_9_= 2.8, *p ≤ 0.009; MK-1064–30 nmol–Escape^S^, Contextual vs Cued, t_4_ = 4.0, ^#^*p* < 0.001; MK-1064–30 nmol–Escape^F^(N= 8), Contextual vs Cued, t_7_ = 3.5, ^#^*p* ≤ 0.002)

As hormonal fluctuations may be responsible for shifts in stress-related behaviors (Figs. 3D, E, S5-8), we controlled for potential influence by selecting only female animals in proestrus for systemic (subcutaneous) pharmacological experiments (Figs. 10, 11 and 12, S5-8). Treatment with yohimbine (5 mg/kg), an α_2_ adrenergic receptor antagonist known for anxiogenic effects, increased escape latency in females (*n* = 41, Day Effect: F_3,108_ = 17.2, *p* < 0.001; Day 3, Vehicle vs. Yohimbine, t_11_ = 4.9, *p < 0.001; Day 3, MK-1064–1 µmol vs. Yohimbine, t_12_ = 5.8, ^**+**^*p* < 0.001) on the day of drug administration (Fig. 10B), an effect that is similarly seen in male mice [32]. While minor deviations in latency to escape were observed with low doses of an Orx_2_R antagonist (300 nmol & 30 nmol), there were no significant differences compared to vehicle-treated animals (Fig. 10B). However, in the 30 nmol-treated group, we observed disparities in how female mice responded to drug treatment, where we measured a split in behavioral responses: Slow Escape (Escape^S^) and Fast Escape (Escape^F^). These two behavioral responses may reflect preexisting differences in these females, revealed by drug treatment, and therefore may reflect natural phenotypes. Importantly, males also exhibit this type of phenotype specific split in escape latency responses in mice [33], as do both sexes in fish [45]. The females in the Escape^S^ classification exhibited delayed escape behavior (*n* = 26, Treatment Effect: F_3,66_ = 3.9, *p* ≤ 0.02; Day Effect: F_3,66_ = 11.0, *p* < 0.001; Day 3, Vehicle vs. MK-1064–30 nmol – Escape^S^, t_9_ = 2.6, *p ≤ 0.03; Day 3, MK-1064–30 nmol – Escape^F^ vs. MK-1064–30 nmol – Escape^S^, t_11_ = 3.0,!*p* ≤ 0.01) similar to that observed in yohimbine control mice (Fig. 10C). Curiously, Escape males administered the same dose (30 nmol) of the Orx_2_R antagonist did not display the phenotype emergence observed in female mice; however, males already display distinct phenotypes (*n* = 10, Vehicle, F_1,3_ = 0.04, R^2^ = 0.0122, *p* ≥ 0.85; Yohimbine, F_1,3_ = 0.002, R^2^ = 0.0005, *p* ≥ 0.97; Fig. 10D). Further, on the day of drug delivery (Day 3), home cage locomotion was dramatically reduced in yohimbine-treated mice, as fear freezing was promoted, but not in animals given an Orx_2_R antagonist (MK-1064) at varying doses (Fig. S9A). Importantly, home cage locomotion was restored to normal levels in the yohimbine treatment group 24 h after treatment (Fig. S9B). Interestingly, mice receiving iBLA injections of an Orx_2_R antagonist (6 fmol) had significantly less home cage locomotion on Day 3 compared to vehicle- and 60 fmol-treated mice (Fig. S9C). Treatment with an Orx_2_R antagonist altered animal weight on the day of drug administration but was restored to normal levels 24 h later (Fig. S14A). Further, Orx_2_R antagonism had no effect on how much food was consumed (Fig. S14C).

### Orx_2_R antagonist-exposed phenotypes display behavioral differences in SIP test

While female mice administered vehicle, as well as low doses of an Orx_2_R antagonist (300 nmol & 30 nmol), displayed social preference in the SIP test, yohimbine-treated animals exhibited a significant decrease in social interaction time compared to vehicle treated females (*n* = 41, Social Effect: F_1,36_ = 27.8, *p* < 0.001; Social, Vehicle vs. Yohimbine, t_11_ = 3.1, *p ≤ 0.003; Vehicle, Novel vs. Social, t_5_ = 3.8, ^**−**^*p* < 0.001; MK-1064–300 nmol, Novel vs. Social, t_7_ = 2.1, ^**−**^*p* ≤ 0.045; MK-1064–30 nmol, Novel vs. Social, t_12_ = 3.0, ^**−**^*p* ≤ 0.005; Fig. 11A). Mice of the Escape^S^ phenotype also displayed a reduction in social preference (*n* = 26, Social Effect: F_1,16_ = 24.1, *p* < 0.001; Social, Vehicle vs. MK-1064–30 nmol – Escape^S^, t_9_ = 2.7, *p ≤ 0.01; MK-1064–30 nmol – Escape^F^, Novel vs. Social, t_7_ = 2.8, ^**#**^*p* ≤ 0.01) similar to that observed after yohimbine treatment (Fig. 11B). Only vehicle-treated mice, similar to untreated females (Fig. 4C), exhibited a reduction in social aversion (Fig. 11C-D).

### Fear response is altered in female phenotypes emerging after Orx_2_R antagonism

All treatment groups exhibited fear learning behavior (Fig. 12A); however, low dose (30 nmol) Orx_2_R antagonism promoted increased contextual freezing behavior while yohimbine-treated female mice demonstrated enhanced freezing to the cue (*n* = 41, Cued Effect: F_1,36_ = 42.0, *p* < 0.001; Pre-Tone, MK-1064–1 µmol vs. MK-1064–30 nmol, t_18_ = 2.4, ^**+**^*p* ≤ 0.03; Post-Tone, Vehicle vs. Yohimbine, t_11_ = 2.5, *p ≤ 0.03; Vehicle, Contextual vs. cued, t_5_ = 2.5, ^**−**^*p* ≤ 0.05; MK-1064–1 µmol, Contextual vs. cued, t_6_ = 2.4, ^**−**^*p* ≤ 0.02; MK-1064–300 nmol, Contextual vs. cued, t_7_ = 2.5, ^**−**^*p* ≤ 0.02; MK-1064–30 nmol, Contextual vs. cued, t_12_ = 4.7, ^**−**^*p* < 0.001; Yohimbine, Contextual vs. cued, t_6_ = 4.2, ^**−**^*p* < 0.001; Fig. 12A). After low dose Orx_2_R antagonist treatment, mice exhibiting the Escape^S^ phenotype experienced elevated cued freezing compared to vehicle-treated control animals (*n* = 26, Conditioned Effect: F_3,22_ = 44.7, *p* < 0.001; Post-Tone, Vehicle vs. MK-1064–30 nmol – Escape^S^, t_9_ = 2.8, *p ≤ 0.009; MK-1064–30 nmol – Escape^S^, Contextual vs. cued, t_4_ = 4.0, ^**#**^*p* < 0.001; MK-1064–30 nmol – Escape^F^, Contextual vs. cued, t_7_ = 3.5, ^**#**^*p* ≤ 0.002), an effect mimicking yohimbine treatment (Fig. 12B). Together these results suggest that antagonizing Orx_2_R in females initiates phenotype divergence in SAM, SIP test, and fear response behaviors.

### IntraBLA Orx_2_R inhibition modifies phenotypic female behavior

**Fig. 13:**
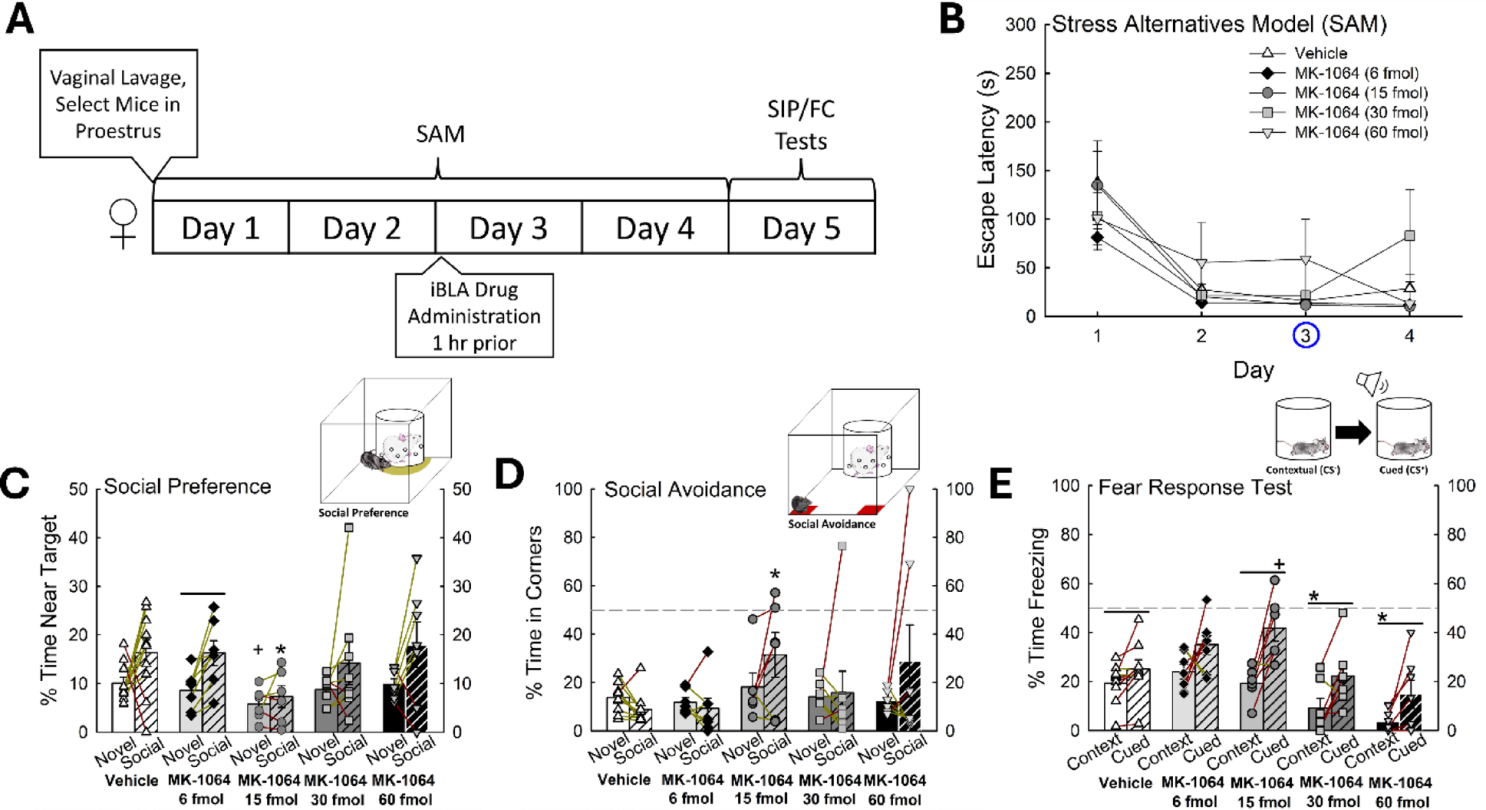
Female exploratory and fear behavior are altered by 15 fmol Orx_2_R antagonist iBLA.** (A) **Experimental design for intra-basolateral amygdala (iBLA) infusion experiments. **(B)** Similar to sc injections, iBLA inhibition of Orx_2_R did not significantly affect escape latency profile in all female mice taken together (N = 38, F_3,99_ = 20.2, *p *< 0.001). **(C)** Female mice treated with iBLA MK-1064 (15 nmol; N = 6) exhibited reduced exploration of novel (N = 16, t_14_=2.2, *p *≤ 0.042) and social (N = 16, t_14_ = 2.3, *p *≤ 0.037) targets. **(D)** Treatment with the 15 fmol dose of the Orx_2_R antagonist MK-1064 resulted in more social aversion behavior compared to vehicle-treated counterparts (N = 16, t_14_ = 3.0, *p *≤ 0.009). **(E)** The 15 fmol dose of MK-1064 infused into the BLA resulted in elevated cued fear freezing relative to control mice (N = 16, t_13_ = 2.6, *p *≤ 0.021)

Doses of the Orx_2_R antagonist MK-1064 that cause somnolescence (30 mg/kg = 65 mM) [76], do not influence sleep, or depress locomotory activity (Fig. S9C), when injected into the BLA (iBLA; Fig. S4). As such, low doses from 20 to 200 nM (6–60 fmol) of MK-1064, when significantly influencing Escape or fear behaviors do so by affecting stress circuitry [25] and not by targeting mechanisms for somnolence. Escape latencies for females, which are typically very fast, were not significantly affected by iBLA Orx_2_R antagonism at any dose (Fig. 13B). However, social preference was significantly decreased (*n* = 38, F_3,99_ = 20.2, *p* < 0.001), and aversion increased with the 15 fmol MK-1064 dose (Fig. 13C, D). Additionally, female mice treated with 15 fmol of Orx_2_R antagonist exhibited significantly more cued fear conditioning (freezing) than vehicle-treated mice (Fig. 13E). Importantly, iBLA infusion of MK-1064 did not alter body weight or food consumption relative to vehicle-treated mice (Fig. S14B, D). These results suggest iBLA Orx_2_R activity to be important for gating stress behavior in female mice.

### BLA *Hcrtr2* and *Adra2a* expression is distinct in pharmacologically induced phenotypes

**Fig. 14:**
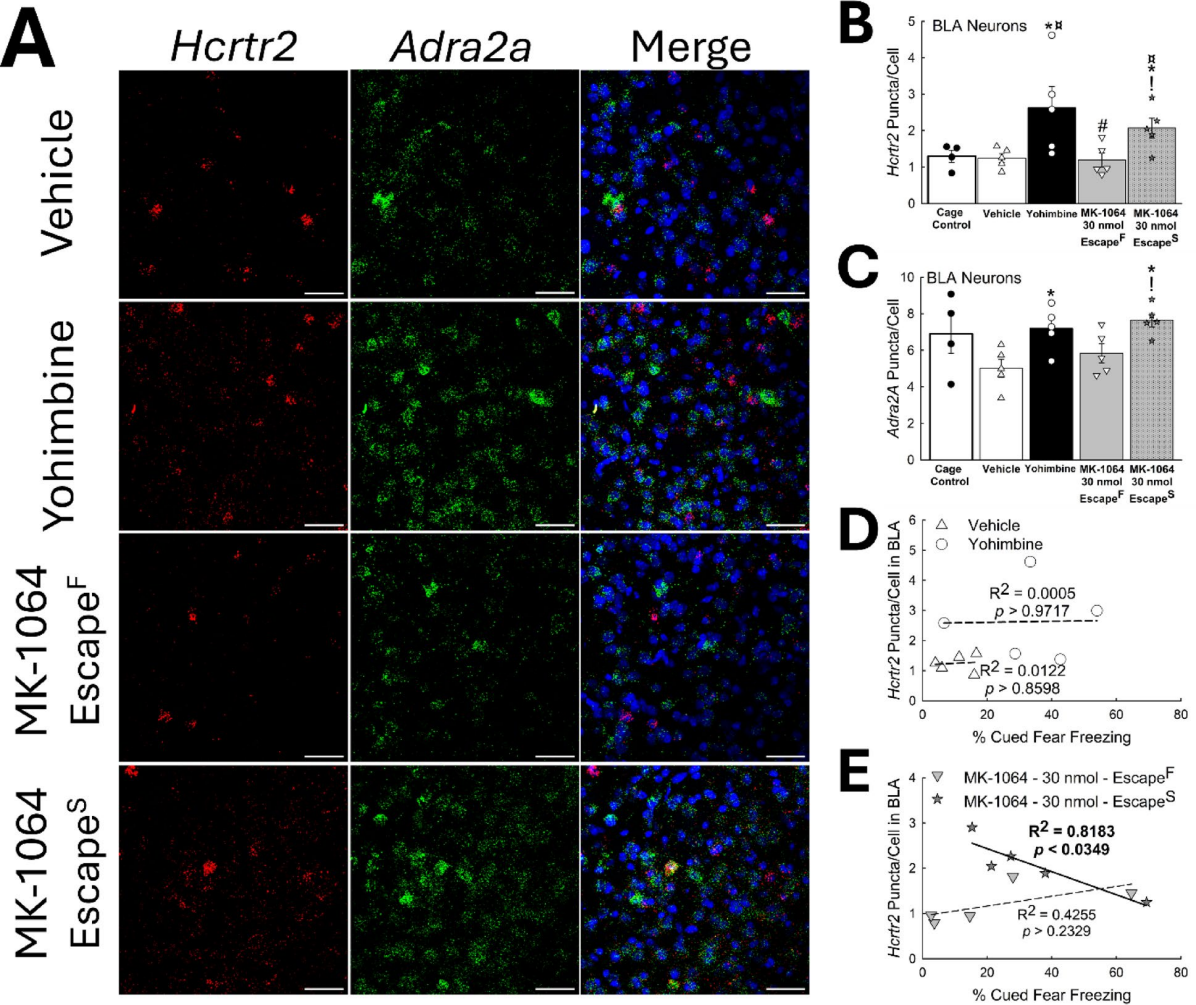
Yohimbine and MK-1064 in female Escape^S^ phenotype mice increases BLA *Hcrtr2* and *Adra2α* expression. **(A)** Representative images of BLA for Vehicle, Yohimbine, and MK-1064-treated mice, including DAPI and RNAscope staining for *Hcrtr2*, and *Adra2a* (Scale Bar = 50 μm). **(B)** In female mice, yohimbine treatment and MK-1064 treatment, for animals exhibiting the Escape^S^ phenotype, results in an increase in intra-BLA *Hcrtr2* puncta/cell (N = 24, F_4,19_ = 3.9, *p* ≤ 0.017; untreated Cage Control vs MK-1064-30 nmol – Escape^S^, t_7_= 2.3, ^¤^*p* ≤ 0.05; Vehicle vs Yohimbine, t_8_= 2.3, *p ≤ 0.049; Vehicle vs MK-1064-30 nmol – Escape^S^, t_8_ = 2.8, *p ≤ 0.024; MK-1064-30 nmol – Escape^F^ vs MK-1064-30 nmol – Escape^S^, t_8_ = 2.7, ^!^*p*≤ 0.029). **(C)** Female animals treated with MK-1064 and exhibiting the slow escape phenotype or those treated with yohimbine expressed higher levels of *Adra2a* puncta/cell in BLA relative to control mice (N = 24, F_4, 19_ = 3.3, *p* ≤ 0.032; Vehicle vs Yohimbine, t_8_= 3.0, *p ≤ 0.017; Vehicle vs MK-1064-30 nmol – Escape^S^, t_8_ = 4.3, *p ≤ 0.003; MK-1064-30 nmol – Escape^F^ vs MK-1064-30 nmol – Escape^S^, t_8_ = 2.8, ^!^*p*≤ 0.022). **(D)** For vehicle- and yohimbine-treated mice, no relationship exists between the amount of intra-BLA Orx_2_R mRNA (represented as *Hcrtr2* puncta normalized by cell count) and cued fear freezing behavior (N = 10, Vehicle, F_1,3_ = 0.04, R^2^ = 0.0122, *p* ≥ 0.8598; Yohimbine, F_1,3_ = 0.002, R^2^ = 0.0005, *p* ≥ 0.9717). **(E)** While there is no correlation between *Hcrtr2* levels in the BLA and cued fear freezing in MK-1064-treated mice exhibiting the Escape^F^ phenotype (N = 5, F_1,3_ = 2.2, R^2^ = 0.4255, *p* ≥ 0.2329), there exists a significant negative correlation in Escape^S^ animals (N = 5, F_1,3_ = 13.5, R^2^ = 0.8183, *p* ≤ 0.0349)

**Fig. 15:**
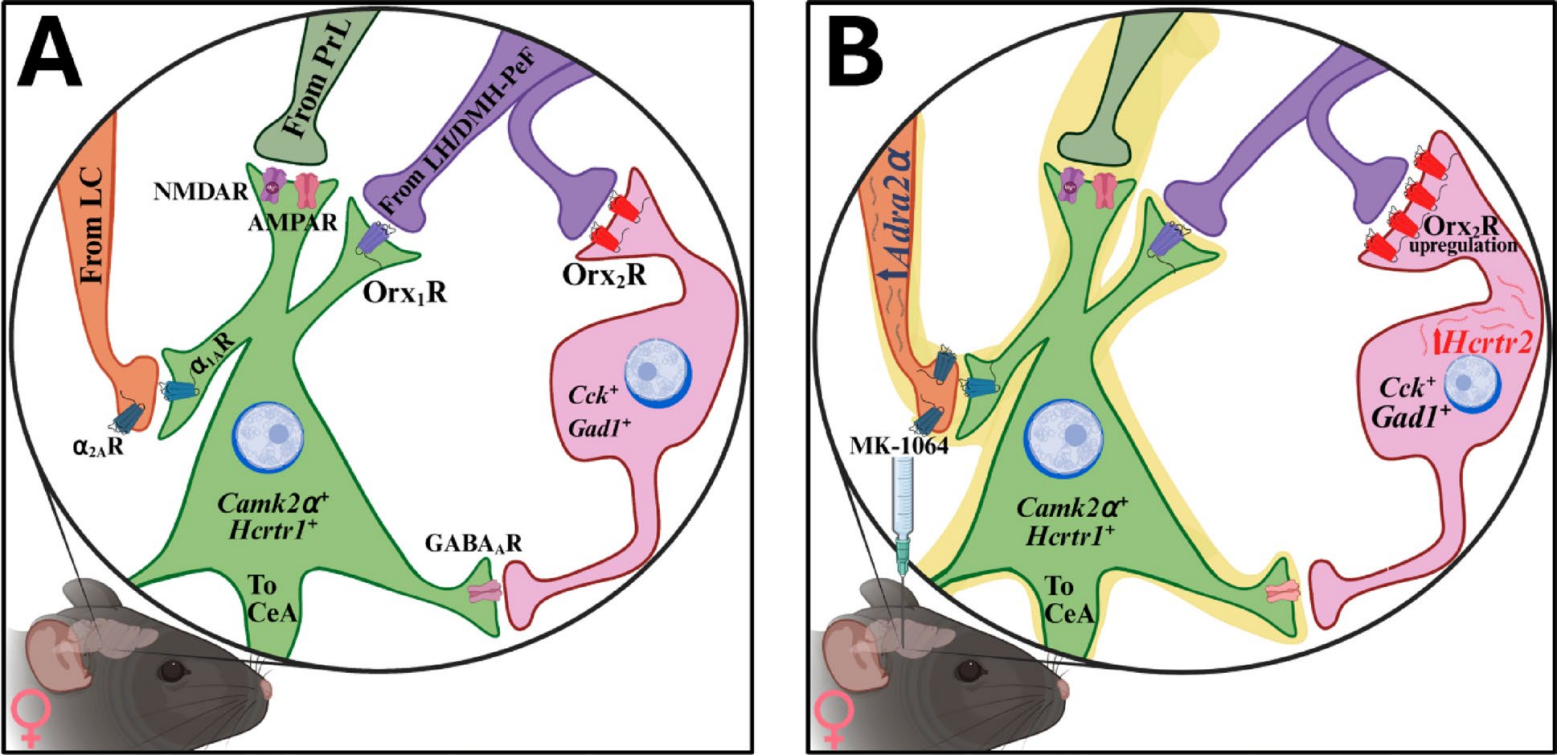
Stress neurocircuitry in the BLA, is modified by Orx and NE. The orexin system works to balance stress neurocircuitry (left panel; A), particularly in the BLA where inputs from the prelimbic area of the prefrontal cortex (PrL), locus coeruleus (LC produces norepinephrine = NE), and lateral/dorsal medial-perifornical region of the hypothalamus (LH/DMH-PeF) converge onto pro-stress glutamatergic neurons (Glu) that project to the central amygdala (CeA). In a subset of female mice administered an Orx_2_R antagonist (MK-1064) that exhibit increased stress behaviors (right panel; B), we predict a homeostatic mechanism attempts to relieve hyperactivation of pro-stress pathways (indicated by yellow highlights) by upregulating *Hcrtr2* (predominantly on CCK^+^ GABA neurons) and *Adra2a*

To predict how stress circuitry is modified [25] during periods of stress in females, we investigated transcriptional changes in the BLA that result from stress exposure and phenotype emergence. The number of cells in the BLA expressing *Hcrtr2* mRNA was elevated in yohimbine-treated and MK-1064-treated Escape^S^ animals (*n* = 24, F_4,19_ = 3.9, *p* ≤ 0.017; untreated Cage Control vs. MK-1064-30 nmol – Escape^S^, t_7_ = 2.3, ^**¤**^*p* ≤ 0.05; Vehicle vs. Yohimbine, t_8_ = 2.3, *p ≤ 0.049; Vehicle vs. MK-1064-30nmol – Escape^S^, t_8_ = 2.8, *p ≤ 0.024; MK-1064-30 nmol – Escape^F^ vs. MK-1064-30 nmol – Escape^S^, t_8_ = 2.7, ^**!**^*p* ≤ 0.029; Figs. 14A-B, S10, S12)), while the overall number of cells in the BLA did not change (Fig. S13). Similarly, BLA expression of adrenergic α_2A_ receptor (*Adra2a*) was elevated in yohimbine- and MK-1064-treated Escape^S^ animals (*n* = 24, F_4, 19_ = 3.3, *p* ≤ 0.032; Vehicle vs. Yohimbine, t_8_ = 3.0, *p ≤ 0.017; Vehicle vs. MK-1064-30 nmol – Escape^S^, t_8_ = 4.3, *p ≤ 0.003; MK-1064-30 nmol – Escape^F^ vs. MK-1064-30 nmol – Escape^S^, t_8_ = 2.8, ^**!**^*p* ≤ 0.022), again the number of cells in the BLA did not change (Fig. S13). Furthermore, Escape^F^ females expressed levels similar to vehicle control animals (Figs. 14A, C, S10, S11). Additionally, mice treated with yohimbine demonstrated higher co-expression of *Hcrtr2* and *Adra2a* relative to vehicle controls, an effect mimicked in Escape^S^, but not Escape^F^, mice (Figs. 14A, B, S10). Further, a significant negative relationship exists between the number of *Hcrtr2* puncta and cued fear freezing behavior in Escape^S^ mice (*n* = 5, F_1,3_ = 13.5, R^2^ = 0.8183, *p* ≤ 0.0349), but not animals of other treatment groups (Figs. 14D-E). These results suggest a physiological difference in MK-1064-induced phenotypes, which may define the stress responsive state in female mice (Fig. 15).

## Discussion

Social defeat vulnerability, as well as resilience, appear to look significantly different in female mice compared with males, with the exception that both males and females exhibit a typical dichotomy of phenotypes. While most females behaviorally make use of escape, in males stress-resilient and vulnerable characteristics are described by Escape and Stay phenotypes, which are typically evenly split. Stay males, which do not use the exits to escape the aggressive social interaction, exhibit reduced motivation to explore, enhanced contextual and cued fear conditioning, greater CR stimulated plasma corticosterone, reduced social preference and heightened social avoidance. This stress-vulnerable phenotype can be reversed through application of anxiolytic drugs or experiences [29, 33, 50]. Here, we demonstrate that although few females exhibit Stay behavior, there exists a stress-vulnerable portion of the population, which display a longer latency and reduced motivation to explore escape routes, decreased social preference, enhanced contextual (but not cued) fear conditioning, and highly elevated plasma corticosterone in response to the CR. The vulnerable female CR-stress hormone response is twice that of vulnerable males. We suggest that these data demonstrate that behavioral and physiological stress responses are distinctively different between females and males. While basal stress hormone (corticosterone) levels are not distinguishable by sex following the fear CR (Fig. 3B) and females respond roughly equally to all four social stress paradigms (Fig. 3C), male and female corticosterone concentrations are not the same on average (Fig. 3D). Looking more closely, some females exhibit an elevated corticosterone response compared to other females (well defined as the high and low responder model [54]) and males, which also have a split in corticosterone secretion between Stay and Escape Phenotypes (Fig. 3E). Thus, behavioral patterns in females (excluding the escape response) and males appear to be aligned with Escape-resilient/Stay-vulnerable corticosterone responses in males and Resilient/Vulnerable corticosterone responses in females. These distinctions likely depend on multifactorial regulatory elements, such as Orx receptor modulation, interplay with other neuromodulatory systems (such as CRF, CCK, endocannabinoids and Neuropeptide S [25, 33, 45, 69]) and mineralocorticoid receptor (MR)/GR expression profiles and reflect different copying strategies, make use of the reciprocal nature of the stress circuitries [77].

In this study, following aggressive SAM social interaction, some females expressed clear neuroendocrine evidence of social defeat, by means of elevated stress hormone corticosterone, more than twice as much as males in response to the fear conditioning CR test (Fig. 3D, E) [43]. This is likely, at least in part, due to differential stress-induced CRF_1_ receptor trafficking, in which males internalize the receptor via association with β-arrestin 2, and females do not [12, 78–81]. Importantly, unstressed plasma corticosterone concentrations were equivalent in males and females (Fig. 3B). However, all four female stress protocols for the SAM, (1) Standard 4-Day SAM, (2) Inescapable social stress, (3) Foreshortened 2-Day SAM, as well as (4) Four days of inescapable stress plus the 4-Day SAM, produced highly elevated corticosterone levels of equivalent magnitude (ranging from 75 to 350 ng/ml; Fig. 2I). While the SAM behavioral paradigm clearly produced elevated stress responses in females, which could be recapitulated by classical conditioning to a much greater degree than in males. This elevation in hypothalamo-pituitary-adrenal axis output is likely very important. Impaired habituation to restraint stress in females, which elevates corticosterone, and binds to GR in the Orx promotor, yields increased expression and activation of Orx in females plus deficits in cognitive flexibility, relative to males [33, 34]. Additionally, 90% of females chose Escape phenotype behavior, even on the first day which is twice the rate of males (Fig. 1D). Thus, while females more often chose the stress-resilient behavior, they did so even while the effects of fear conditioning left their neuroendocrine stress systems very active, as demonstrated after the CR test on the 5th day. This elevated HPA axis response to the CR seems counterintuitive considering in males anxiolytic drugs (such as the CRF_1_ receptor antagonist antalarmin) reverse the stress-vulnerable Stay phenotype in multiple species [32, 45, 82].

Establishing a preclinical protocol to study social defeat or stressful social interaction in females is a high priority [83] for those interested in psychological disorders such as anxiety, depression, and PTSD [21, 22–], as females show a disproportionately higher incidence of affective diagnoses in the human population [2, 4]. The Stress Alternatives Model [28–32, 43, 45] reveals evidence of vulnerable and resilient phenotypes in both females and males, even while both sexes receive equivalent levels of aggression during social defeat (Fig. 2C, D), before escape dramatically relieves the effect of social stress, for those that choose it. While some females produce considerably elevated plasma corticosterone concentrations in response to the CR test compared to Stay/vulnerable males (Fig. 3D, E; not evident in baseline levels, Fig. 3B), both sexes are split into two distinctive hormone response groups, where resilient individuals (of either sex) have equivalently low corticosterone response to CR. This doubling of stress hormone reaction is due to a conditioned response measured on Day 5, in the absence of the aggressor (US; Fig. 1C) [32, 43], with the variability suggesting that individuals exhibiting the highest corticosterone are relatively more stress-vulnerable than those with lower classical conditioning-induced corticosterone levels. High responding, more stress-vulnerable females also exhibit a longer latency to and reduced exploration of escape routes, lower rates of social preference, and increased contextual fear conditioning similar to stress-vulnerable Stay males.

Thus, both sexes develop behavioral phenotypes with explicitly stress-resilient and stress-vulnerable characteristics, even though the apparent integration of stress perception and reactivity is fundamentally different between the sexes. While a greater proportion of females make use of active avoidance (Escape) and do so more quickly than males (Figs. 1D and 2A), additional inescapable stress makes female responses more like those of males (Figs. 1E and 2B). That is, in female mice, more social stress results in more expression of the Stay phenotype. After an additional four days of inescapable stress, the phenotypes are more balanced between Escape and Stay (Fig. 1E), and females are slower when they do escape (Fig. 2B). This suggests that slower escape is an important measure of stress-vulnerability in females (Fig. 10B, C), as it is in males given anxiogenic drugs [32, 45, 81].

In addition to enhanced stress, estrous stage influences social interaction , such that proestrus and estrus favor social preference (Fig. 5B), and proestrus also blunts the effects of cued and contextual fear conditioning (Fig. 6C). Compared to males, females exhibit higher levels of social preference and lower contextual and cued freezing than both Escape and Stay males, even though stress-vulnerable females have elevated contextual fear conditioning compared to resilient females. These behaviors are also characterized by phenotypic differences, with Stay males and stress-vulnerable females having significantly lower social preference, but higher contextual and cued (males) freezing.

Female stress-related phenotypes emerge following systemic treatment with the anxiogenic drug yohimbine (5 mg/kg), or the lowest dose (30 nmol) of Orx_2_R antagonist MK-1064, which slows escape in some females (Escape^S^), but not in others (Escape^F^). Antagonism of the Orx_2_ receptors (via MK-1064) dramatically increases social aversion , and increases cued and contextual freezing, but only in Escape^S^ females. Injection of MK-1064 directly into the BLA, at a dose of 15 fmol, recapitulates these findings from systemic injections. In both females and males, *Hcrtr2* is highly co-expressed in cholecystokinin-containing (*Cck*) neurons. Little is known about CCK interneurons or their function in the BLA [85], we presume that those in which *Cck* colocalizes with *Hcrtr2* are GABAergic, as the largest proportion of *Hcrtr2* expression is in GABA neurons [29]. However, BLA CCK interneurons are heterogeneous and comprise three distinct subtypes with passive and active membrane properties. While CCK is known to activate Orx neurons via CCK_A(1)_ receptors [86], and Orx_1_R make heterodimer proteins with CCK_A(1)_R [87], whereas CCK_2_R appear to be anxiogenic [88], little is known about the relationship between *Cck* and *Hcrtr2*. By contrast, co-expression was not common in somatostatin (*Som*) BLA neurons. The percentage of BLA neurons expressing *Hcrtr2* was increased by systemic anxiogenic treatments, yohimbine and inhibition of Orx_2_R with 30 nmol MK-1064, with Escape^S^ females expressing a significantly greater percentage of *Hcrtr2-*expressing BLA neurons than Escape^F^ female mice. These results suggest sex-dependent changes to stress neurocircuitries, including elements in the BLA, are critical for the development of stress-differentiated affective responsiveness.

The BLA stress neurocircuitry consists of two major elements: (1) Glutamatergic neurons that are innervated by similar pyramidal neurons from the Prelimbic Cortex and project to the Periaqueductal Gray, Central Amygdala, and Hippocampus, and produce pro-stress-related physiological and behavioral reactions, and (2) GABAergic, anti-stress neurons that inhibit these Principal Neurons limiting behavioral and physiological stress responses, as well as other GABAergic neurons [25, 29, 51, 70]. Thus, these pro- and anti-stress neurocircuitries include those divided functionally into prelimbic (PrL) prefrontal cortical connections to anterior BLA (aBLA, pro-stress) and infralimbic (IL) connections to posterior regions (pBLA) marked by *Rspo2* and *Ppp1r1b* gene expression, respectively [40, 84]. Importantly, other parallel neurocircuits that involve Orx signaling can influence emotion [25, 41]. In BLA of males, Orx_1_R are found in a minority of cells, which are primarily glutamatergic (*CamKII*-expressing) pyramidal cells [29]. The location and relationship of Orx_2_R in BLA contrast with Orx_1_R, in that they are primarily found in GABAergic neurons in these circuits [27], suggesting opposing functions for these two receptor types [84].

We demonstrate pro-stress, depressive and anxiogenic actions of low-dose Orx_2_R inhibition delivered systemically (Figs. 10, 11 and 12) and directly into the BLA (Fig. 13) [27, 29]. Correspondingly, intracerebroventricular (icv) injection of Orx_2_R agonists produces anxiolytic and antidepressive effects (in SAM) [56], with anxiogenic responses following inhibition of Orx_2_R [58]. Results from systemic, icv, and intra-BLA Orx_2_R treatments, taken together, suggest a generally anti-stress, anxiolytic, and anti-depressive profile for promotion of Orx_2_R outcomes. However, higher-dose antagonism of Orx_2_R delivered systemically improves NREM and REM sleep in animals, including humans [76, 89, 90]; it may concurrently also produce self-reported antidepressive effects [89]. Importantly, chemogenetic activation of Orx activity in mice (presumably including Orx_2_R actions) facilitates emergence from anesthesia and reduced pain sensitivity [91]. Clearly, sleep deprivation and pain impact anxious and depressive behaviors, potentially obfuscating the role for Orx_2_R in specific treatments. Additionally, Orx_2_R null mice display enhanced behavioral despair [92] and reduced contextual freezing [93], while the Orx_2_R knockout reduces stress responsivity [94]. Inhibiting Orx_1_R reduces fear/panic-induced freezing [95–98], as well as decreasing anxious and depressive behavior [29]. Responses may be brain region specific [70, 99] as Orx_2_R activity in the nucleus accumbens shell, PrL, and paraventricular thalamus may enhance anxious behavior [100–102]. The collective qualities of Orx_2_R action suggest that, unlike Orx_1_R activity (consistently linked to pro-stress outcomes) [95, 96, 103], Orx_2_R differentially regulate behavioral responses [104]. Importantly, although other Orx circuits exist that do not exhibit opposing Orx_1_R and Orx_2_R functional relationships [41, 93, 105], and do not necessarily follow Orx_1_R pro-stress and Orx_2_R anti-stress profiles that we demonstrate in the BLA [27, 29, 52, 58, 84], systemic (Figs. 10, 11 and 12), icv [56], and intra-BLA (Fig. 13) delivery of drugs acting on Orx_2_R have effects that suggest Orx_2_R have anxiolytic and antidepressive properties. However, not all doses of the Orx_2_R antagonist MK-1064 had the same effects, which may suggest differential recruitment of circuit components and receptors. Critically, Orx_2_R (*Hcrtr2* expression) in the BLA are mostly found in *Cck/Gad1* positive neurons (Fig. 9G), but they are also found on a very small percentage of *CamkIIα* neurons. Pro-stress *CamkIIα/Hcrtr1* neurons are found in close proximity with *Cck/Gad1/Hcrtr2* anti-stress neurons in locally constructed microcircuits, such that Orx_2_R agonists inhibit stress. For the dose response experiments, high MK-1064 doses may have affected both receptor types despite distinctive affinity profiles. These data suggest that careful dosage considerations for oral therapies might be necessary for Orx_2_R treatment of affective disorders.

We suspected these behavioral differences in females and males might be associated with Orx_2_R function and attempted to manipulate escape-related behaviors in females pharmacologically. Systemic inhibition of Orx_2_R (with MK-1064 doses under the amount necessary to promote sleep [106] or depressed locomotor activity; Fig. S9) modified behaviors specifically associated with phenotype (Fig. 10C): delayed escape (Escape^S^) and faster escape (Escape^F^). Female Escape^S^ mice additionally displayed pro-stress responses, which mimicked those triggered by the anxiogenic α_2_ receptor antagonist yohimbine, in SIP and Fear Response tests (Figs. 11 and 12). Importantly, Orx_2_R inhibition with MK-1064 delivered directly to the BLA, decreases social preference, increases social aversion , and elevates cued fear conditioning. Although the lowest dose of systemic Orx_2_R antagonist modified behavior in Escape^S^ females, it had no effect on female Escape^F^ mice.

The orexin system is plastic [106], including the number of hypothalamic cells that produce orexins fluctuate diurnally [108] and during drug dependency [108–110]. Potentially, this flexible orexin production reserve [108] is coordinated with changes in target receptivity, such that gene expression and receptor signaling reflect changes in orexin reserve. We have shown plasticity in Orx_1_R and Orx_2_R gene expression associated with stress-related behavioral phenotypes and Orx_1_R antagonism [29]. Here, we demonstrate a mechanism for potential plasticity of Orx_2_R-expressing cells in the BLA [106] that may define animals’ stress responsive state (Figs. 9, 10, 11, 12, 13, 14 and 15), and to that point, male Escape mice have higher *Hcrtr2* expression in the BLA compared to Stay animals [29]. In females, phenotype emergence based on the high/low glucocorticoid responder model, potentially a preexisting phenotypic difference, appears to promote neurophysiological adaptations and plasticity [106] within the BLA-contained orexin system. As such, higher *Hcrtr2*-expressing cells in the BLA may act as a homeostatic mechanism to help establish balance to counteract a bias in pro-stress signaling.

## Conclusion

Stress responses are modified by Orx_2_R activity in the BLA. Stress-vulnerable females exhibit a delay and reduction in exploration of escape routes, elevated contextual fear conditioning, as well as reduced social preference, compared to resilient females, and by manipulating Orx_2_R activity in females causes further emergence of alternative behavioral phenotypes. Systemic delivery of MK-1064 to promote Orx_2_R inhibition was comparable in behavioral effect to direct delivery of MK-1064 to Orx_2_R in the BLA. Behavioral and phenotypic differences are further defined by the number of *Hcrtr2*- and *Adra2a*-expressing cells in the BLA. Importantly, peripheral drug delivery resulted in altered molecular signaling and gene expression within the stress neurocircuitry of the BLA. Together, these results suggest the balance of Orx_2_R activity in the BLA is important for mediating stress responsivity in both males and females.

## Supplementary Information

Supplementary Material 1

Supplementary Material 2

Supplementary Material 3

## Data Availability

All data supporting the findings of this study are available within the paper and its Supplementary Information.

## References

[1] Kessler RC. Epidemiology of women and depression. J Affect Disord. 2003;74(1):5–13.10.1016/S0165-0327(02)00426-3. 12646294 10.1016/s0165-0327(02)00426-3

[2] Kroenke K, Spitzer RL, Williams JB, Monahan PO, Lowe B. Anxiety disorders in primary care: prevalence, impairment, comorbidity, and detection. Ann Intern Med. 2007;146:5:317–25.10.7326/0003-4819-146-5-200703060-00004. 17339617 10.7326/0003-4819-146-5-200703060-00004

[3] Berton O, Nestler EJ. New approaches to antidepressant drug discovery: beyond monoamines. Nat Rev Neurosci. 2006;7:2.10.1038/nrn1846. 10.1038/nrn1846 16429123

[4] Albert PR. Why is depression more prevalent in women? J Psychiatry Neuroscience: JPN. 2015;40:4. 10.1503/jpn.150205 PMC4478054 26107348

[5] Bjorkqvist K. Social defeat as a stressor in humans. Physiol Behav. 2001;73(3):435–42.http://www.sciencedirect.com/science/article/pii/S0031938401004905. PM:11438372. 11438372 10.1016/s0031-9384(01)00490-5

[6] Kessler RC. The effects of stressful life events on depression. Ann Rev Psychol. 1997;48:191–214.10.1146/annurev.psych.48.1.191. 9046559 10.1146/annurev.psych.48.1.191

[7] Brown GW, Prudo R. Psychiatric disorder in a rural and an urban population: 1. Aetiology of depression. Psychol Med. 1981;11:3.10.1017/s0033291700052880. 10.1017/s0033291700052880 7267889

[8] Bangasser DA. Sex differences in stress-related receptors: ‘’micro’’ differences with ‘’macro’’ implications for mood and anxiety disorders. Biology Sex Differences. 2013;4(1:2).10.1186/2042-6410-4-2. 10.1186/2042-6410-4-2 PMC3556142 23336736

[9] Bangasser DA, Cuarenta A. Sex differences in anxiety and depression: circuits and mechanisms. Nat Rev Neurosci. 2021;22:11.10.1038/s41583-021-00513-0. 10.1038/s41583-021-00513-0 34545241

[10] Jiang T, Feng M, Hutsell A, Luscher B. Sex-specific GABAergic microcircuits that switch vulnerability into resilience to stress and reverse the effects of chronic stress exposure. Mol Psychiatry. 2024.10.1038/s41380-024-02835-8. 39550416 10.1038/s41380-024-02835-8 PMC12092295

[11] Brivio E, Lopez JP, Chen A. Sex differences: transcriptional signatures of stress exposure in male and female brains. Genes, brain, and behavior. 2020;19:3e12643;10.1111/gbb.12643 10.1111/gbb.12643 31989757

[12] Bangasser DA, Valentino RJ. Sex differences in molecular and cellular substrates of stress. Cell Mol Neurobiol. 2012;32:5.10.1007/s10571-012-9824-4. 10.1007/s10571-012-9824-4 PMC3378920 22488525

[13] Valentino RJ, Reyes B, Van Bockstaele E, Bangasser D. Molecular and cellular sex differences at the intersection of stress and arousal. Neuropharmacology. 2012;62:1:13–20.10.1016/j.neuropharm.2011.06.004. 21712048 10.1016/j.neuropharm.2011.06.004 PMC3184353

[14] Bhargava A, Arnold AP, Bangasser DA, Denton KM, Gupta A, Hilliard Krause LM, et al. Considering sex as a biological variable in basic and clinical studies: an endocrine society scientific statement. Endocr Rev. 2021;42:3219–58.10.1210/endrev/bnaa034. 10.1210/endrev/bnaa034 PMC8348944 33704446

[15] Blanchard DC, Fukunaga-Stinson C, Takahashi LK, Flannelly KJ, Blanchard RJ. Dominance and aggression in social groups of male and female rats. Behav Process. 1984;9:1:31–48.10.1016/0376-6357(84)90006-8. 10.1016/0376-6357(84)90006-8 24923827

[16] Krishnan V, Han MH, Graham DL, Berton O, Renthal W, Russo SJ, et al. Molecular adaptations underlying susceptibility and resistance to social defeat in brain reward regions. Cell. 2007;131:2:391–404.10.1016/j.cell.2007.09.018. 17956738 10.1016/j.cell.2007.09.018

[17] Berton O, McClung CA, Dileone RJ, Krishnan V, Renthal W, Russo SJ, et al. Essential role of BDNF in the mesolimbic dopamine pathway in social defeat stress. Science. 2006;311:5762:864–8. PM:16469931. 16469931 10.1126/science.1120972

[18] Huhman KL. Social conflict models: can they inform Us about human psychopathology? Horm Behav. 2006;50:4:640–6. doi:S0018-506X(06)00174-7 [pii] 1016/j.yhbeh.2006.06.022. 16870189 10.1016/j.yhbeh.2006.06.022

[19] Nestler EJ, Hyman SE. Animal models of neuropsychiatric disorders. Nat Neurosci. 2010;13:10. 10.1038/nn.2647 PMC3750731 20877280

[20] Nestler EJ, Russo SJ. Neurobiological basis of stress resilience. Neuron. 2024;112:12.10.1016/j.neuron.2024.05.001. 10.1016/j.neuron.2024.05.001 PMC11189737 38795707

[21] Lin D, Boyle MP, Dollar P, Lee H, Lein ES, Perona P, et al. Functional identification of an aggression locus in the mouse hypothalamus. Nature. 2011;470:221.https://doi.org/10.1038/nature09736.https://www.nature.com/articles/nature09736#supplementary-information. 21307935 10.1038/nature09736 PMC3075820

[22] Harris AZ, Atsak P, Bretton ZH, Holt ES, Alam R, Morton MP, et al. A novel method for chronic social defeat stress in female mice. Neuropsychopharmacology. 2018;43:6. 10.1038/npp.2017.259 PMC5916350 29090682

[23] Newman EL, Covington HE III, Suh J, Bicakci MB, Ressler KJ, DeBold JF et al. Fighting females: neural and behavioral consequences of social defeat stress in female mice. Biol Psychiatry. 2019;86:657-668. 10.1016/j.biopsych.2019.05.005 PMC6788975 31255250

[24] Shively CA, Laber-Laird K, Anton RF. Behavior and physiology of social stress and depression in female cynomolgus monkeys. Biol Psychiatry. 1997;41:8.10.1016/S0006-3223(96)00185-0. 10.1016/S0006-3223(96)00185-0 9099414

[25] Korzan WJ, Summers CH. Evolution of stress responses refine mechanisms of social rank. Neurobiol Stress. 2021;14:100328.10.1016/j.ynstr.2021.100328. 33997153 10.1016/j.ynstr.2021.100328 PMC8105687

[26] Blanchard DC, Summers CH, Blanchard RJ. The role of behavior in translational models for psychopathology: functionality and dysfunctional behaviors. Neurosci Biobehav Rev. 2013;37:8:1567–77.10.1016/j.neubiorev.2013.06.008. 23791787 10.1016/j.neubiorev.2013.06.008 PMC3800172

[27] Yaeger JDW, Krupp KT, Summers TR, Summers CH. Contextual generalization of social stress learning is modulated by orexin receptors in basolateral amygdala. Neuropharmacology. 2022;215:109168.10.1016/j.neuropharm.2022.109168. 35724928 10.1016/j.neuropharm.2022.109168 PMC9285878

[28] Smith JP, Achua JK, Summers TR, Ronan PJ, Summers CH. Neuropeptide S and BDNF gene expression in the amygdala are influenced by social decision-making under stress. Front Behav Neurosci. 2014;8:121.10.3389/fnbeh.2014.00121. 24782729 10.3389/fnbeh.2014.00121 PMC3986560

[29] Yaeger JDW, Krupp KT, Jacobs BM, Onserio BO, Meyerink BL, Cain JT, et al. Orexin 1 receptor antagonism in the basolateral amygdala shifts the balance from Pro- to antistress signaling and behavior. Biol Psychiatry. 2022;91:9841–52.10.1016/j.biopsych.2021.12.019. 10.1016/j.biopsych.2021.12.019 PMC9020795 35279280

[30] Robertson JM, Prince MA, Achua JK, Carpenter RE, Arendt DH, Smith JP, et al. Nuance and behavioral cogency: how the visible burrow system inspired the Stress-Alternatives model and conceptualization of the continuum of anxiety. Physiol Behav. 2015;146:86–97.10.1016/j.physbeh.2015.03.036. 26066728 10.1016/j.physbeh.2015.03.036 PMC4584205

[31] Krupp KT, Yaeger JDW, Ledesma LJ, Withanage MHH, Gale JJ, Howe CB, et al. Single administration of a psychedelic [(R)-DOI] influences coping strategies to an escapable social stress. Neuropharmacology. 2024;252:109949.10.1016/j.neuropharm.2024.109949. 38636726 10.1016/j.neuropharm.2024.109949 PMC11073902

[32] Smith JP, Prince MA, Achua JK, Robertson JM, Anderson RT, Ronan PJ, et al. Intensity of anxiety is modified via complex integrative stress circuitries. Psychoneuroendocrinology. 2016;63:351–61.10.1016/j.psyneuen.2015.10.016. 26555428 10.1016/j.psyneuen.2015.10.016 PMC4838407

[33] Grafe LA, Bhatnagar S. The contribution of orexins to sex differences in the stress response. Brain Res. 2020;1731:145893.10.1016/j.brainres.2018.07.026. 30081036 10.1016/j.brainres.2018.07.026 PMC6360123

[34] Grafe LA, Cornfeld A, Luz S, Valentino R, Bhatnagar S. Orexins mediate sex differences in the stress response and in cognitive flexibility. Biol Psychiatry. 2017;81.10.1016/j.biopsych.2016.10.013. :8:683 – 92; doi. 10.1016/j.biopsych.2016.10.013 PMC5359079 27955897

[35] Sakurai T, Amemiya A, Ishii M, Matsuzaki I, Chemelli RM, Tanaka H, et al. Orexins and orexin receptors: a family of hypothalamic neuropeptides and G protein-coupled receptors that regulate feeding behavior. Cell. 1998;92:4573–85. 10.1016/s0092-8674(00)80949-6 9491897

[36] Aston-Jones G, Smith RJ, Moorman DE, Richardson KA. Role of lateral hypothalamic orexin neurons in reward processing and addiction. Neuropharmacology. 2009;56:112–21. 18655797 10.1016/j.neuropharm.2008.06.060 PMC2635332

[37] Sakurai T. The role of orexin in motivated behaviours. Nat Rev Neurosci. 2014;15:11. 10.1038/nrn3837 25301357

[38] James MH, Mahler SV, Moorman DE, Aston-Jones G. A decade of Orexin/Hypocretin and addiction: where are we now? Current topics in behavioral neurosciences. 2017;33:247–81;10.1007/7854_2016_57 10.1007/7854_2016_57 PMC5799809 28012090

[39] Giardino WJ, de Lecea L. Hypocretin (orexin) neuromodulation of stress and reward pathways. Curr Opin Neurobiol. 2014;29:103–8.10.1016/j.conb.2014.07.006. 25050887 10.1016/j.conb.2014.07.006 PMC4267967

[40] Kim J, Pignatelli M, Xu S, Itohara S, Tonegawa S. Antagonistic negative and positive neurons of the basolateral amygdala. Nat Neurosci. 2016;19:12:1636–46.10.1038/nn.4414. 27749826 10.1038/nn.4414 PMC5493320

[41] Giardino WJ, Eban-Rothschild A, Christoffel DJ, Li SB, Malenka RC, de Lecea L. Parallel circuits from the bed nuclei of stria terminalis to the lateral hypothalamus drive opposing emotional States. Nat Neurosci. 2018;21:8.10.1038/s41593-018-0198-x. 10.1038/s41593-018-0198-x PMC6095688 30038273

[42] Arendt DH, Smith JP, Bastida CC, Prasad MS, Oliver KD, Eyster KM, et al. Contrasting hippocampal and amygdalar expression of genes related to neural plasticity during escape from social aggression. Physiol Behav. 2012;107(5):670–9.10.1016/j.physbeh.2012.03.005. 22450262 10.1016/j.physbeh.2012.03.005 PMC4372993

[43] Carpenter RE, Summers CH. Learning strategies during fear conditioning. Neurobiol Learn Mem. 2009;91:4415–23. 10.1016/j.nlm.2009.01.009 PMC2762607 19340951

[44] Smith JP, Achua JK, Summers TR, Ronan PJ, Summers CH. Neuropeptide S and BDNF gene expression in the amygdala are influenced by social decision-making under stress. Front Behav Neurosci. 2014;8:121:1–13. ; doi:Artn 121 Doi 10.3389/Fnbeh.2014.00121. 24782729 10.3389/fnbeh.2014.00121 PMC3986560

[45] Carpenter RE, Sabirzhanov B, Summers TR, Clark TG, Keifer J, Summers CH. Anxiolytic reversal of classically conditioned / chronic stress-induced gene expression and learning in the stress alternatives model. Behav Brain Res. 2023;440:114258.10.1016/j.bbr.2022.114258. 36521572 10.1016/j.bbr.2022.114258 PMC9872777

[46] John MM, Pratt MA, Yaeger JDW, Brummels RA, Ledesma LJ, Meyer LS, et al. Aggression as a contributing factor to social defeat and stress vulnerability. Neurobiol Stress. 2025;36:100728: 1–13.10.1016/j.ynstr.2025.100728. 10.1016/j.ynstr.2025.100728 PMC12245451 40642451

[47] Staton CD, Yaeger JDW, Khalid D, Haroun F, Fernandez BS, Fernandez JS, et al. Orexin 2 receptor stimulation enhances resilience, while orexin 2 Inhibition promotes susceptibility, to social stress, anxiety and depression. Neuropharmacology. 2018;143:79–94.10.1016/j.neuropharm.2018.09.016. 30240784 10.1016/j.neuropharm.2018.09.016 PMC6275094

[48] Yaeger JDW, John MM, Ledesma LJ, Krupp KT, Booth CD, Jones NT, et al. Acute carbamoylated erythropoietin reduces social stress-induced anxiety and depression-related behaviors. Neuropharmacology. 2025;278:110558:1–14.10.1016/j.neuropharm.2025.110558. 10.1016/j.neuropharm.2025.110558 PMC12258813 40514007

[49] Yaeger JDW, Achua JK, Booth CD, Khalid D, John MM, Ledesma LJ, et al. Learned phenotypes emerge during social stress modifying hippocampal orexin receptor gene expression. Sci Rep. 2024;14(1:31691):1–17.10.1038/s41598-024-81590-w. 39738291 10.1038/s41598-024-81590-w PMC11685668

[50] Yaeger JDW, Krupp KT, Gale JJ, Summers CH. Counterbalanced microcircuits for Orx_1_ and Orx_2_ regulation of stress reactivity. Med Drug Discovery. 2020;100059:1–20.

[51] Pang T, Yaeger JDW, Summers CH, Mitra R. Cardinal role of the environment in stress iInduced changes across life stages and generations. Neurosci Biobehavioral Reviews. 2021;124:137–50. 10.1016/j.neubiorev.2021.01.012 PMC9286069 33549740

[52] Cockrem JF. Individual variation in glucocorticoid stress responses in animals. Gen Comp Endocrinol. 2013;181:45–58.10.1016/j.ygcen.2012.11.025. 23298571 10.1016/j.ygcen.2012.11.025

[53] Kabbaj M. Neurobiological bases of individual differences in emotional and stress responsiveness: high responders–Low responders model. Arch Neurol. 2004;61:7:1009–12.10.1001/archneur.61.7.1009. 15262729 10.1001/archneur.61.7.1009

[54] Nater UM, Moor C, Okere U, Stallkamp R, Martin M, Ehlert U, et al. Performance on a declarative memory task is better in high than low cortisol responders to psychosocial stress. Psychoneuroendocrinology. 2007;32.10.1016/j.psyneuen.2007.05.006. :6:758 – 63; doi. 10.1016/j.psyneuen.2007.05.006 17606328

[55] Staton CD, Yaeger JD, Khalid D, Haroun F, Fernandez BS, Fernandez JS, et al. Orexin 2 receptor stimulation enhances resilience, while orexin 2 Inhibition promotes susceptibility, to social stress, anxiety and depression. Neuropharmacology. 2018;143:79–94. 30240784 10.1016/j.neuropharm.2018.09.016 PMC6275094

[56] Yaeger JDW, Krupp KT, Jacobs BM, Onserio BO, Meyerink BL, Cain JT et al. Orexin 1 Receptor Antagonism in the Basolateral Amygdala Shifts the Balance from Pro- to Anti-stress Signaling and Behavior. Biological Psychiatry. 2021;*in revision*. 10.1016/j.biopsych.2021.12.019 PMC9020795 35279280

[57] Arendt DH, Hassell J, Li H, Achua JK, Guarnieri DJ, DiLeone RJ, et al. Anxiolytic function of the orexin 2/hypocretin A receptor in the basolateral amygdala. Psychoneuroendocrinology. 2014;40:17–26. 24485472 10.1016/j.psyneuen.2013.10.010 PMC4361941

[58] McLean AC, Valenzuela N, Fai S, Bennett SAL. Performing vaginal lavage, crystal Violet staining, and vaginal cytological evaluation for mouse estrous cycle staging identification. J Visualized Experiments: JoVE. 2012;e674389–e.10.3791/4389. 10.3791/4389 PMC3490233 23007862

[59] Roecker AJ, Mercer SP, Schreier JD, Cox CD, Fraley ME, Steen JT, et al. Discovery of 5′′-Chloro-N-[(5,6-dimethoxypyridin-2-yl)methyl]-2,2′:5′,3′′-terpyridine-3′-carboxamide (MK-1064): A selective Orexin 2 receptor antagonist (2-SORA) for the treatment of insomnia. ChemMedChem. 2014;9:2311–22.10.1002/cmdc.201300447. 10.1002/cmdc.201300447 24376006

[60] Secci ME, Reed T, Quinlan V, Gilpin NW, Avegno EM. Quantitative analysis of gene expression in RNAscope-processed brain tissue. Bio Protoc. 2023;13:1e4580.10.21769/BioProtoc.4580. 10.21769/BioProtoc.4580 PMC9901451 36789089

[61] Veazie PJ. When to combine hypotheses and adjust for multiple tests. Health Serv Res. 2006;41.10.1111/j.1475-6773.2006.00512.x. 3 Pt 1:804 – 18. 10.1111/j.1475-6773.2006.00512.x PMC1713204 16704513

[62] Feise RJ. Do multiple outcome measures require p-value adjustment? BMC Med Res Methodol. 2002;2:1.10.1186/1471-2288-2-8. 12069695 10.1186/1471-2288-2-8 PMC117123

[63] Moran MD. Arguments for rejecting the sequential bonferroni in ecological studies. Oikos. 2003;100:2:403–5.10.1034/j.1600-0706.2003.12010.x.

[64] Nakagawa S. A farewell to bonferroni: the problems of low statistical power and publication bias. Behav Ecol. 2004;15:6:1044–5.10.1093/beheco/arh107.

[65] Perneger TV. What’s wrong with bonferroni adjustments. BMJ. 1998;316:7139:1236–8.10.1136/bmj.316.7139.1236. 9553006 10.1136/bmj.316.7139.1236 PMC1112991

[66] Rothman KJ. No adjustments are needed for multiple comparisons. Epidemiology. 1990;1:1.https://journals.lww.com/epidem/Fulltext/1990/01000/No_Adjustments_Are_Needed_for_Multiple_Comparisons.10.aspx. 2081237

[67] Jennions MD, Møller AP. A survey of the statistical power of research in behavioral ecology and animal behavior. Behav Ecol. 2003;14:3438–45.10.1093/beheco/14.3.438.

[68] Robertson JM, Achua JK, Smith JP, Prince MA, Staton CD, Ronan PJ, et al. Anxious behavior induces elevated hippocampal Cb_2_ receptor gene expression. Neuroscience. 2017;352:273–84.10.1016/j.neuroscience.2017.03.061. 28392296 10.1016/j.neuroscience.2017.03.061 PMC5482502

[69] Summers CH, Yaeger JDW, Staton CD, Arendt DH, Summers TR. Orexin/hypocretin receptor modulation of anxiolytic and antidepressive responses during social stress and decision-making: potential for therapy. Brain Res. 2020;1731:146085.10.1016/j.brainres.2018.12.036. 30590027 10.1016/j.brainres.2018.12.036 PMC6591110

[70] Grafe LA, Bhatnagar S. The contribution of orexins to sex differences in the stress response. Brain Res. 2020;1731:145893.10.1016/j.brainres.2018.07.026. 30081036 10.1016/j.brainres.2018.07.026 PMC6360123

[71] Grafe LA, Cornfeld A, Luz S, Valentino R, Bhatnagar S. Orexins mediate sex differences in the stress response and in cognitive flexibility. Biol Psychiatry. 2017;81:8.10.1016/j.biopsych.2016.10.013. 10.1016/j.biopsych.2016.10.013 PMC5359079 27955897

[72] Lovick TA, Zangrossi H. Effect of estrous cycle on behavior of females in rodent tests of anxiety. Front Psychiatry. 2021;12:1492.10.3389/fpsyt.2021.711065. 10.3389/fpsyt.2021.711065 PMC8438218 34531768

[73] Poitras M, Lebeau M, Plamondon H. The cycle of stress: A systematic review of the impact of chronic psychological stress models on the rodent estrous cycle. Neurosci Biobehavioral Reviews. 2024;162:105730.10.1016/j.neubiorev.2024.105730. 10.1016/j.neubiorev.2024.105730 38763179

[74] Vereczki VK, Müller K, Krizsán É, Máté Z, Fekete Z, Rovira-Esteban L, et al. Total number and ratio of GABAergic neuron types in the mouse lateral and basal amygdala. J Neurosci. 2021;41:21:4575–95.10.1523/jneurosci.2700-20.2021. 33837051 10.1523/JNEUROSCI.2700-20.2021 PMC8260245

[75] Gotter AL, Forman MS, Harrell CM, Stevens J, Svetnik V, Yee KL, et al. Orexin 2 receptor antagonism is sufficient to promote NREM and REM sleep from mouse to man. Sci Rep. 2016;6:27147.10.1038/srep27147. 27256922 10.1038/srep27147 PMC4891657

[76] de Kloet ER, de Kloet SF, de Kloet CS, de Kloet AD. Top-down and bottom-up control of stress-coping. J Neuroendocrinol. 2019;31:3.10.1111/jne.12675. 10.1111/jne.12675 PMC6519262 30578574

[77] Bangasser DA, Curtis A, Reyes BAS, Bethea TT, Parastatidis I, Ischiropoulos H, et al. Sex differences in corticotropin-releasing factor receptor signaling and trafficking: potential role in female vulnerability to stress-related psychopathology. Mol Psychiatry. 2010;15:9:896–904.10.1038/mp.2010.66. 10.1038/mp.2010.66 PMC2935505 20548297

[78] Edwards HM, Wallace CE, Gardiner WD, Doherty BM, Harrigan RT, Yuede KM, et al. Sex-dependent effects of acute stress on amyloid-β in male and female mice. Brain. 2023;146:6:2268–74.10.1093/brain/awad052. 37127299 10.1093/brain/awad052 PMC10232275

[79] Oakley RH, Olivares-Reyes JA, Hudson CC, Flores-Vega F, Dautzenberg FM, Hauger RL. Carboxyl-terminal and intracellular loop sites for CRF1 receptor phosphorylation and beta-arrestin-2 recruitment: a mechanism regulating stress and anxiety responses. Am J Physiol Regul Integr Comp Physiol. 2007;293(1):R209–22.10.1152/ajpregu.00099.2006. 17363685 10.1152/ajpregu.00099.2006 PMC3102763

[80] Waters RP, Rivalan M, Bangasser DA, Deussing JM, Ising M, Wood SK, et al. Evidence for the role of corticotropin-releasing factor in major depressive disorder. Neurosci Biobehav Rev. 2015;58:63–78.10.1016/j.neubiorev.2015.07.011. 26271720 10.1016/j.neubiorev.2015.07.011 PMC4828243

[81] Summers TR, Summers TL, Carpenter RE, Smith JP, Young SL, Meyerink B, et al. Learning and CRF-Induced indecision during escape and submission in rainbow trout during socially aggressive interactions in the Stress-Alternatives model. Front NeuroSci. 2017;11:515.10.3389/fnins.2017.00515. 28966574 10.3389/fnins.2017.00515 PMC5605647

[82] Palanza P P. Animal models of anxiety and depression: how are females different? Neurosci Biobehav Rev. 2001;25(3):219–33.10.1016/s0149-7634(01)00010-0. 11378178 10.1016/s0149-7634(01)00010-0

[83] Yaeger JDW, Krupp KT, Gale JJ, Summers CH. Counterbalanced microcircuits for Orx1 and Orx2 regulation of stress reactivity. Med Drug Discovery. 2020;8:100059.10.1016/j.medidd.2020.100059.

[84] Jasnow AM, Ressler KJ, Hammack SE, Chhatwal JP, Rainnie DG. Distinct subtypes of cholecystokinin (CCK)-Containing interneurons of the basolateral amygdala identified using a CCK Promoter-Specific lentivirus. J Neurophysiol. 2009;101:31494–506.10.1152/jn.91149.2008. 10.1152/jn.91149.2008 PMC2666417 19164102

[85] Tsujino N, Yamanaka A, Ichiki K, Muraki Y, Kilduff TS, Yagami K-i, et al. Cholecystokinin activates Orexin/Hypocretin neurons through the cholecystokinin A receptor. J Neurosci. 2005;25:32:7459–69.10.1523/jneurosci.1193-05.2005. 16093397 10.1523/JNEUROSCI.1193-05.2005 PMC6725310

[86] Bai B, Chen X, Zhang R, Wang X, Jiang Y, Li D, et al. Dual-agonist occupancy of orexin receptor 1 and cholecystokinin A receptor heterodimers decreases G-protein–dependent signaling and migration in the human colon cancer cell line HT-29. Biochimica et biophysica acta (BBA) -. Mol Cell Res. 2017;1864:7.10.1016/j.bbamcr.2017.03.003. 10.1016/j.bbamcr.2017.03.003 28288880

[87] Wang H, Wong PTH, Spiess J, Zhu YZ. Cholecystokinin-2 (CCK2) receptor-mediated anxiety-like behaviors in rats. Neurosci Biobehavioral Reviews. 2005;29:8:1361–73.10.1016/j.neubiorev.2005.05.008. 10.1016/j.neubiorev.2005.05.008 16120463

[88] Recourt K, de Boer P, Zuiker R, Luthringer R, Kent J, van der Ark P, et al. The selective orexin-2 antagonist seltorexant (JNJ-42847922/MIN-202) shows antidepressant and sleep-promoting effects in patients with major depressive disorder. Translational Psychiatry. 2019;9:1216.10.1038/s41398-019-0553-z. 10.1038/s41398-019-0553-z PMC6722075 31481683

[89] Li SB, Nevarez N, Giardino WJ, de Lecea L. Optical probing of orexin/hypocretin receptor antagonists. Sleep. 2018;41:10.10.1093/sleep/zsy141. 10.1093/sleep/zsy141 PMC6454482 30060151

[90] Zhou W, Cheung K, Kyu S, Wang L, Guan Z, Kurien PA, et al. Activation of orexin system facilitates anesthesia emergence and pain control. Proc Natl Acad Sci USA. 2018;115:E4510740–7.10.1073/pnas.1808622115. 10.1073/pnas.1808622115 PMC6233126 30348769

[91] Scott MM, Marcus JN, Pettersen A, Birnbaum SG, Mochizuki T, Scammell TE, et al. Hcrtr1 and 2 signaling differentially regulates depression-like behaviors. Behav Brain Res. 2011;222:2289–94. 10.1016/j.bbr.2011.02.044 PMC3474296 21377495

[92] Soya S, Takahashi TM, McHugh TJ, Maejima T, Herlitze S, Abe M, et al. Orexin modulates behavioral fear expression through the locus coeruleus. Nat Commun. 2017;8:1.10.1038/s41467-017-01782-z. 29151577 10.1038/s41467-017-01782-z PMC5694764

[93] Yun S, Wennerholm M, Shelton JE, Bonaventure P, Letavic MA, Shireman BT, et al. Selective Inhibition of Orexin-2 receptors prevents Stress-Induced ACTH release in mice. Front Behav Neurosci. 2017;11:83.10.3389/fnbeh.2017.00083. 28533747 10.3389/fnbeh.2017.00083 PMC5420581

[94] Salehabadi S, Abrari K, Salmani ME, Nasiri M, Lashkarbolouki T. Investigating the role of the amygdala orexin receptor 1 in memory acquisition and extinction in a rat model of PTSD. Behav Brain Res. 2020;384:112455. 32044404 10.1016/j.bbr.2019.112455

[95] Flores Á, Valls-Comamala V, Costa G, Saravia R, Maldonado R, Berrendero F. The hypocretin/orexin system mediates the extinction of fear memories. Neuropsychopharmacology. 2014;39:12. 10.1038/npp.2014.146 PMC4200503 24930888

[96] Johnson PL, Truitt W, Fitz SD, Minick PE, Dietrich A, Sanghani S, et al. A key role for orexin in panic anxiety. Nat Med. 2010;16:1. 20037593 10.1038/nm.2075 PMC2832844

[97] Johnson PL, Samuels BC, Fitz SD, Federici LM, Hammes N, Early MC, et al. Orexin 1 receptors are a novel target to modulate panic responses and the panic brain network. Physiol Behav. 2012;107:5733–42.10.1016/j.physbeh.2012.04.016. 10.1016/j.physbeh.2012.04.016 PMC3472124 22554617

[98] Dustrude ET, Caliman IF, Bernabe CS, Fitz SD, Grafe LA, Bhatnagar S, et al. Orexin depolarizes central amygdala neurons via orexin receptor 1, phospholipase C and Sodium-Calcium exchanger and modulates conditioned fear. Front NeuroSci. 2018;12:934.10.3389/fnins.2018.00934. 30618563 10.3389/fnins.2018.00934 PMC6305451

[99] Li Y, Li S, Wei C, Wang H, Sui N, Kirouac GJ. Orexins in the paraventricular nucleus of the thalamus mediate anxiety-like responses in rats. Psychopharmacology. 2010;212:2251–65. 10.1007/s00213-010-1948-y 20645079

[100] Li B, Chang L, Peng X. Orexin 2 receptor in the nucleus accumbens is critical for the modulation of acute stress-induced anxiety. Psychoneuroendocrinology. 2021;131:105317. 34111776 10.1016/j.psyneuen.2021.105317

[101] Soares VP, de Andrade TG, Canteras NS, Coimbra NC, Wotjak CT, Almada RC. Orexin 1 and 2 receptors in the prelimbic cortex modulate threat valuation. Neuroscience. 2021;468:158–67. 10.1016/j.neuroscience.2021.06.006 34126185

[102] Flores A, Herry C, Maldonado R, Berrendero F. Facilitation of contextual fear extinction by Orexin-1 receptor antagonism is associated with the activation of specific amygdala cell subpopulations. Int J Neuropsychopharmacol / official Sci J Collegium Int Neuropsychopharmacologicum. 2017;20:8.10.1093/ijnp/pyx029. 10.1093/ijnp/pyx029 PMC5570099 28453642

[103] Grafe LA, Eacret D, Dobkin J, Bhatnagar S. Reduced Orexin System Function Contributes to Resilience to Repeated Social Stress. eNeuro. 2018:ENEURO. 0273-17.2018. 10.1523/ENEURO.0273-17.2018 PMC5900465 29662948

[104] Sears RM, Fink AE, Wigestrand MB, Farb CR, de Lecea L, Ledoux JE. Orexin/hypocretin system modulates amygdala-dependent threat learning through the locus coeruleus. Proc Natl Acad Sci USA. 2013;110:50:20260–5.10.1073/pnas.1320325110. 24277819 10.1073/pnas.1320325110 PMC3864341

[105] Roecker AJ, Reger TS, Mattern MC, Mercer SP, Bergman JM, Schreier JD, et al. Discovery of MK-3697: a selective orexin 2 receptor antagonist (2-SORA) for the treatment of insomnia. Bioorg Med Chem Lett. 2014;24:20:4884–90.10.1016/j.bmcl.2014.08.041. 25248679 10.1016/j.bmcl.2014.08.041

[106] Yaeger JDW, James MH, Summers CH. Functional plasticity of orexin/hypocretin neurons balances stress states. Biological psychiatry. 2026;in press. 10.1016/j.biopsych.2025.11.004 PMC12768429 41248749

[107] McGregor R, Shan L, Wu MF, Siegel JM. Diurnal fluctuation in the number of hypocretin/orexin and Histamine producing: implication for Understanding and treating neuronal loss. PLoS ONE. 2017;12:6:e0178573.10.1371/journal.pone.0178573. 28570646 10.1371/journal.pone.0178573 PMC5453544

[108] James MH, Aston-Jones G. Orexin reserve: A mechanistic framework for the role of orexins (hypocretins) in addiction. Biol Psychiatry. 2022;92:11.10.1016/j.biopsych.2022.06.027. 10.1016/j.biopsych.2022.06.027 PMC10184826 36328706

[109] Thannickal TC, John J, Shan L, Swaab DF, Wu MF, Ramanathan L, et al. Opiates increase the number of hypocretin-producing cells in human and mouse brain and reverse cataplexy in a mouse model of narcolepsy. Sci Transl Med. 2018;10:447.10.1126/scitranslmed.aao4953. 10.1126/scitranslmed.aao4953 PMC8235614 29950444

[110] James MH, Stopper CM, Zimmer BA, Koll NE, Bowrey HE, Aston-Jones G. Increased number and activity of a lateral subpopulation of hypothalamic Orexin/Hypocretin neurons underlies the expression of an addicted state in rats. Biol Psychiatry. 2019;85:11925–35.10.1016/j.biopsych.2018.07.022. 10.1016/j.biopsych.2018.07.022 PMC7528037 30219208

