# Supplementary material for: Social defeat stress responses in the stress alternative model are dependent on sex and anterior basolateral amygdala orexin 2 receptors
Source: Biol Sex Differ. 2026 Mar 15;17:87. doi: 10.1186/s13293-026-00858-0 (PMC13104226; doi:10.1186/s13293-026-00858-0)
Supplement: Supplementary file 1 — Supplementary Material 1 [file 13293_2026_858_MOESM1_ESM.docx]

Sex differences in social stress perception and responsiveness are critical to vulnerability or resilience

Supplementary material

Jazmine D.W. Yaeger^1^, Megan M. John^2,3,4^, Leighton J. Ledesma^5^, Trent L. Greschke^2^, J. J. Gale^2,3^, Lauren S. Meyer^2^, Renee A. Brummels^2^, Wayne J. Korzan^6^, R. Parrish Waters^7^, and

Cliff H. Summers^2,3,4*^

^1^Pediatrics and Rare Diseases Group, Sanford Research, Sioux Falls, SD 57104 USA, ^2^Department of Biology, University of South Dakota, Vermillion, SD 57069 USA, ^3^Neuroscience Group, Division of Biomedical and Translational Sciences, Sanford School of Medicine, University of South Dakota, Vermillion, SD 57069 USA, ^4^Veterans Affairs Research Service, Sioux Falls VA Health Care System, Sioux Falls, SD 57105 USA, ^5^IDP in Biomedical Sciences, Medical College of Wisconsin, Milwaukee, WI 53226, USA, ^6^Department of Biological and Environmental Sciences, The University of West Alabama, Livingston, AL 34570 USA, ^7^Department of Biological Sciences, University of Mary Washington, Fredericksburg, VA 22401 USA

^*^Corresponding author: Cliff H. Summers, Ph.D.

Nolop Distinguished Professor

Department of Biology

University of South Dakota

414 East Clark Street

Vermillion, SD 57069-2390

605 658 6716 [cliff@usd.edu](mailto:cliff@usd.edu)

Short title: Social defeat in females

Number of pages: 14

Supplemental Figs: 13


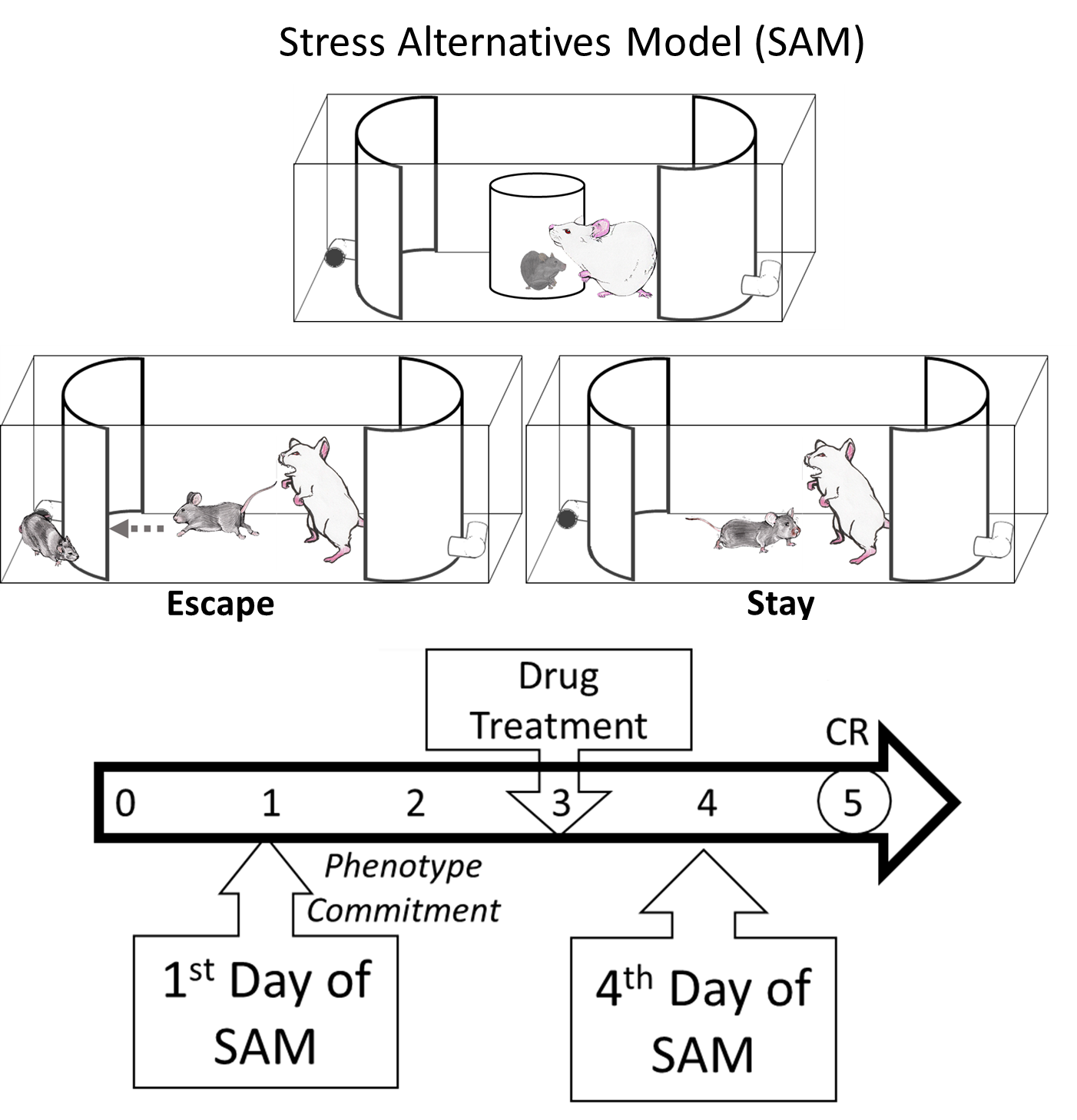


**Fig. S1.** SAM social stress paradigm timeline for male dyad interactions. On Day 5, CR refers to testing for the Pavlovian conditioned response (CS = tone, presented in the absence of the US = aggressive interaction).

**
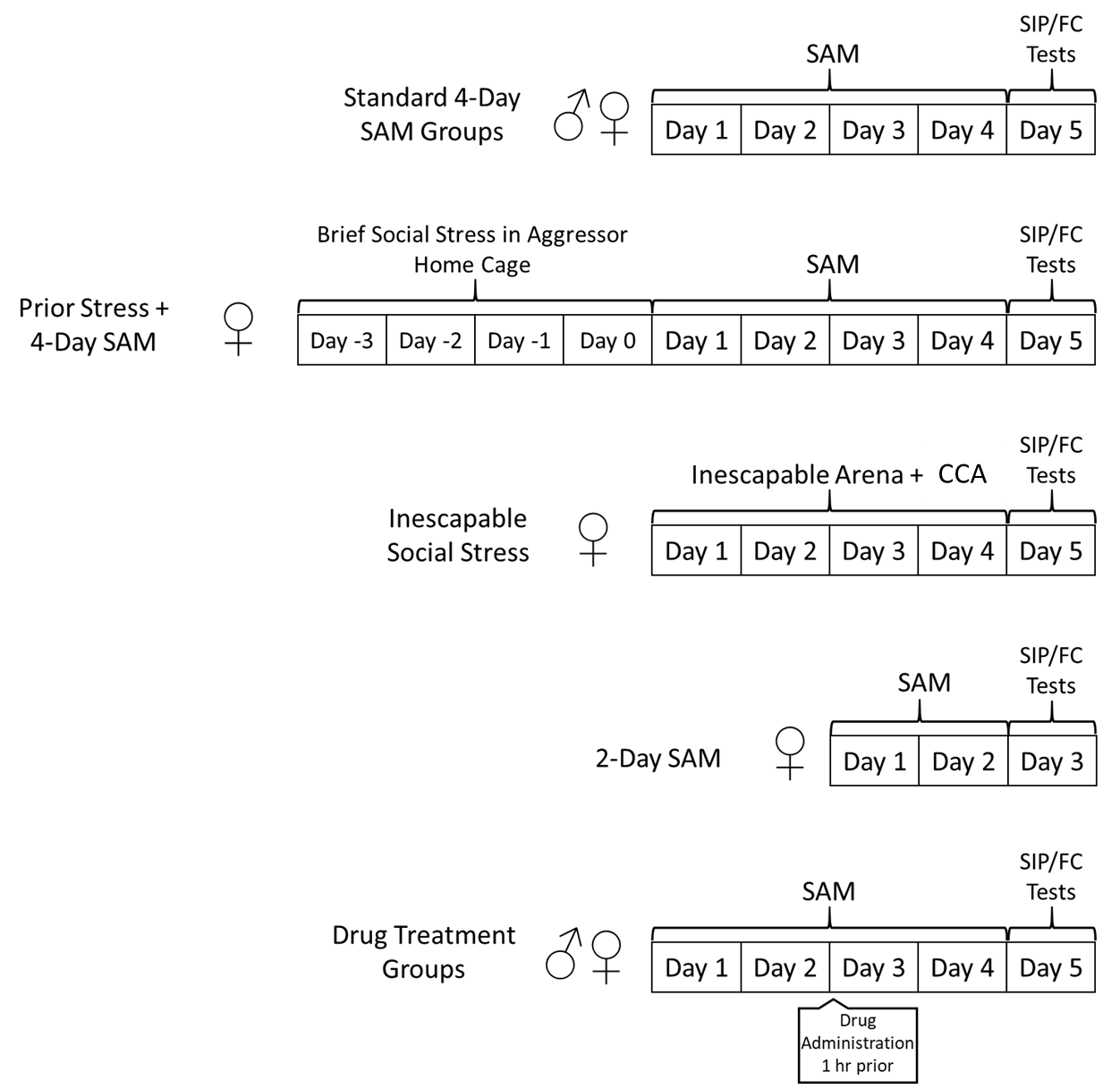
**

**Fig. S2.** Social stress paradigms for examining female social stress.


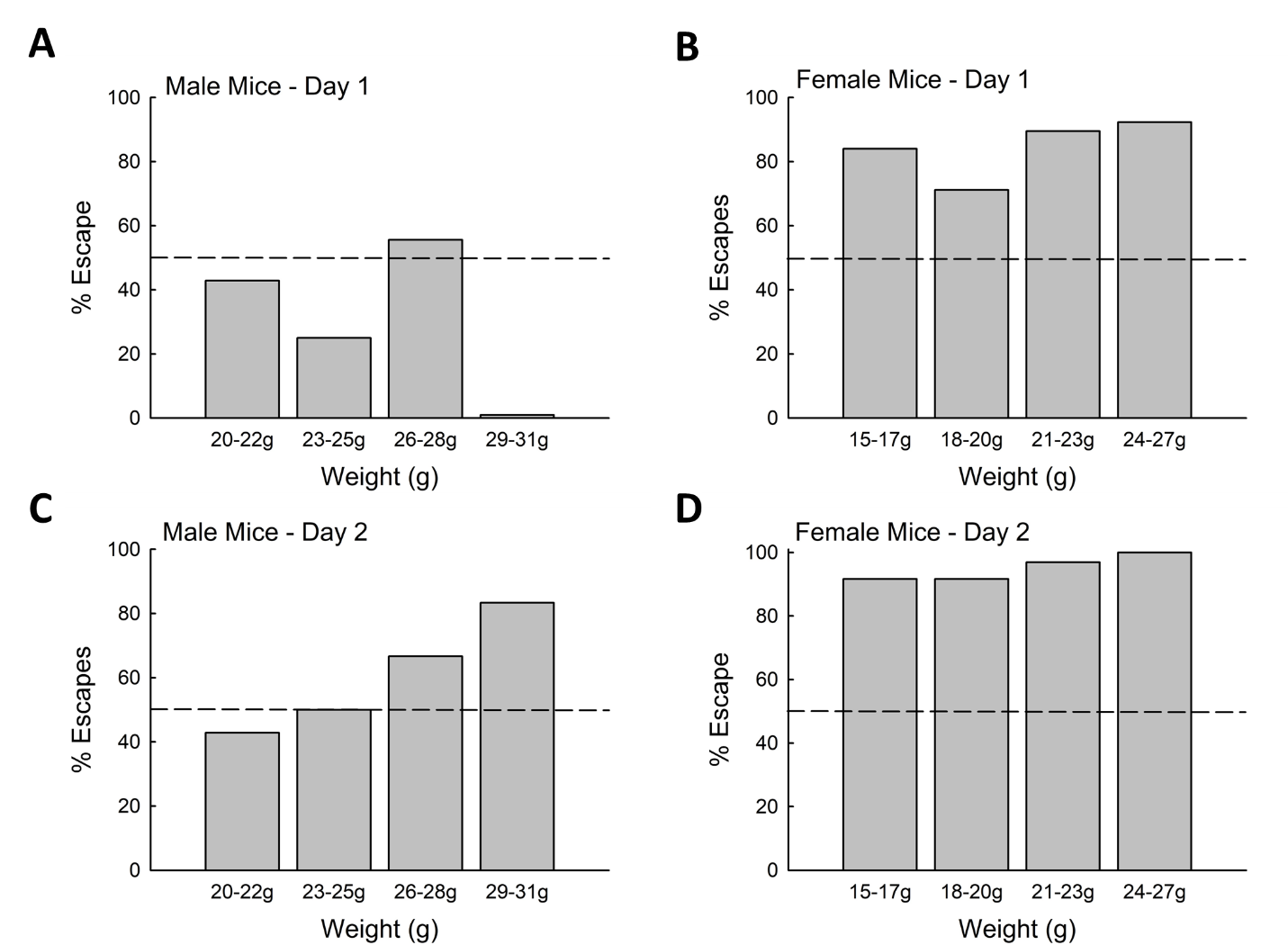


**Fig. S3.** Weight of male and female mice did not influence ability to escape.


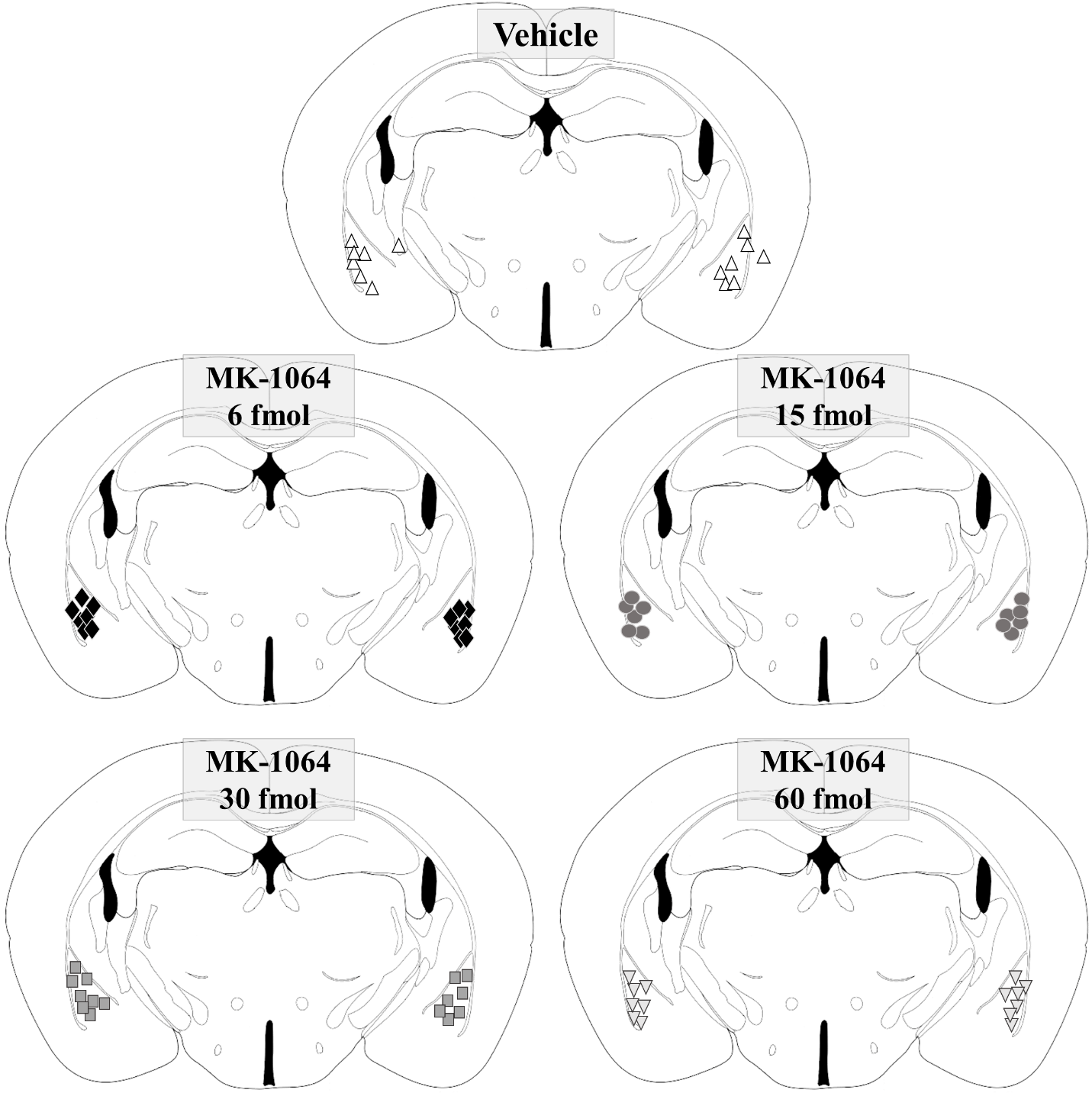


**Fig. S4.** BLA targeted injection sites (bottom of cannulae) for pharmacology infusions in test mice.


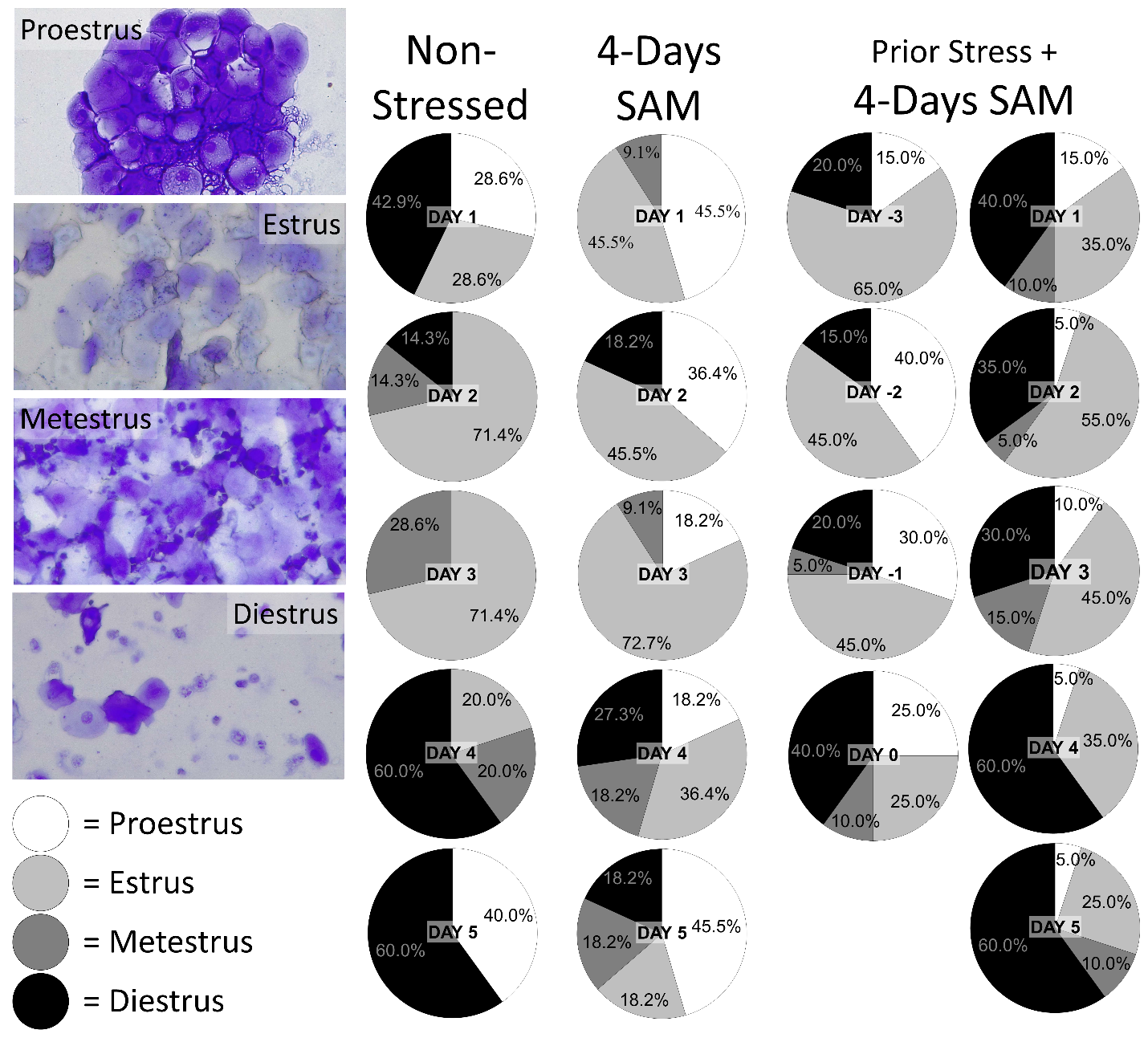


**Fig. S5.** Lavage samples were collected daily to determine the stage of the estrous cycle for Non-Stressed Cage Control mice and those of experimental groups: 4-Days SAM & Prior Stress + 4-Days SAM.


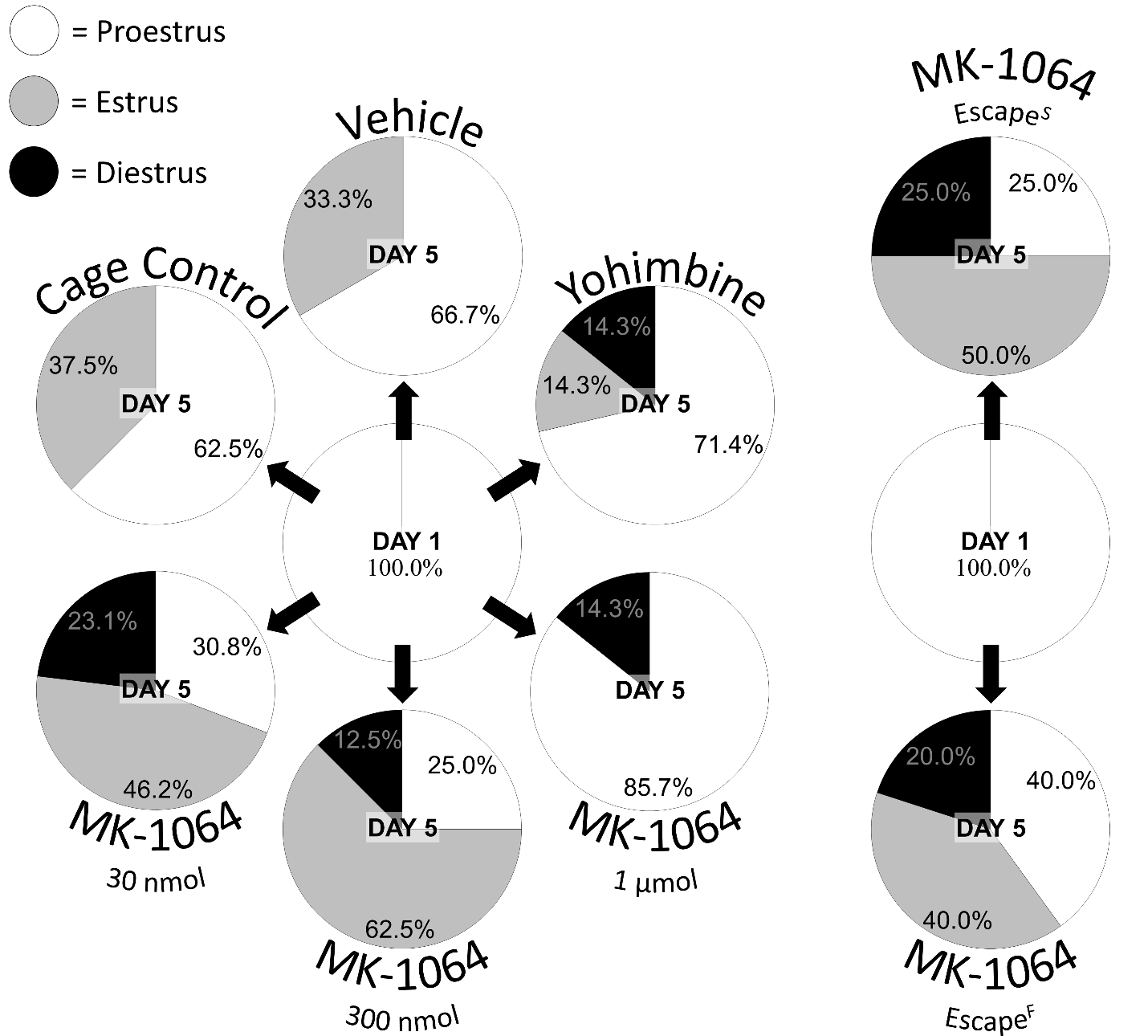


**Fig. S6.** While all animals started experimental trials in the proestrus stage of the estrous cycle, variation was observed in the number of animals in a particular stage of the cycle after drug treatment (Yohimbine or MK-1064).

**
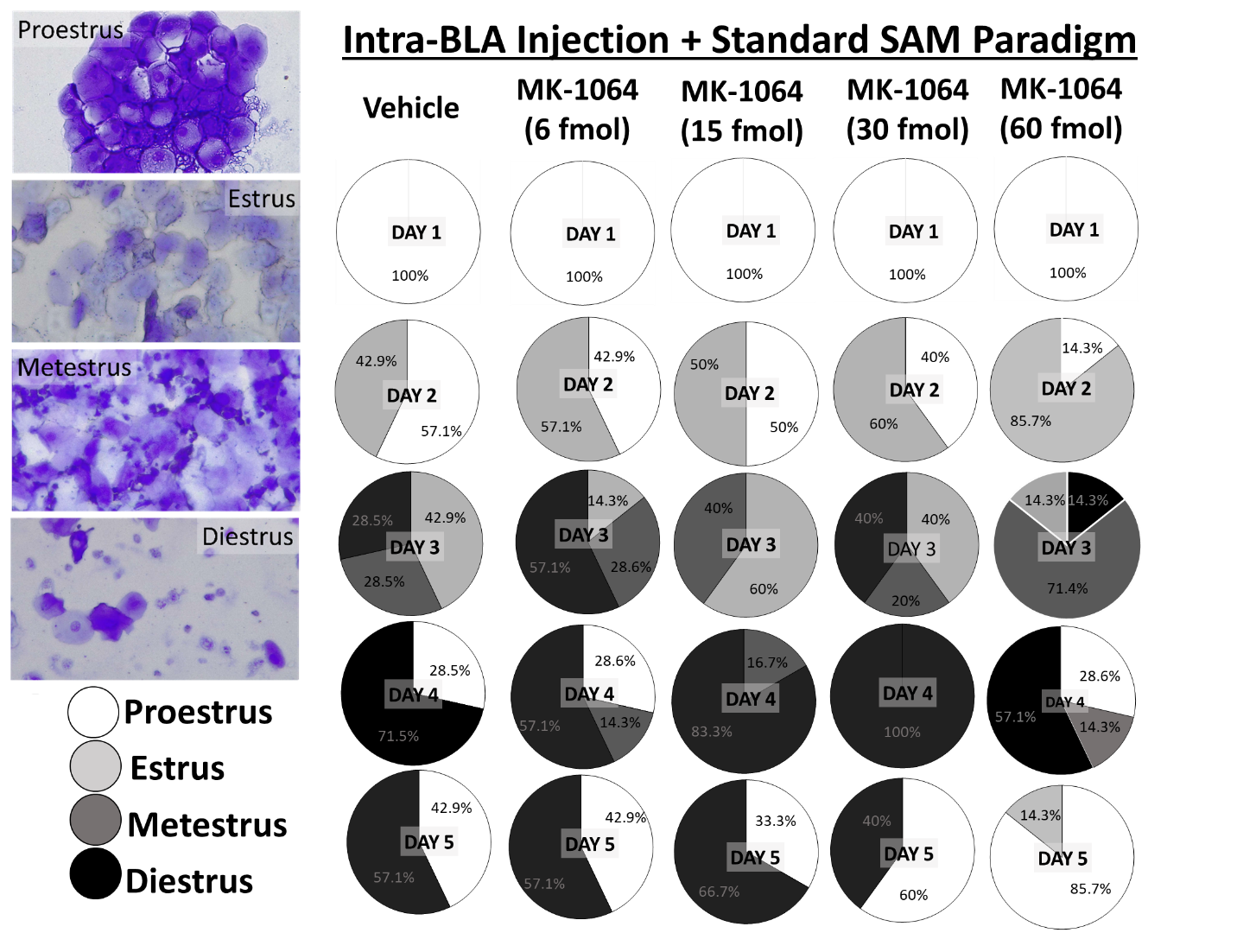
**

**Fig. S7.** Lavage samples were collected daily to determine the stage of the estrous cycle for females receiving intra-BLA injections.


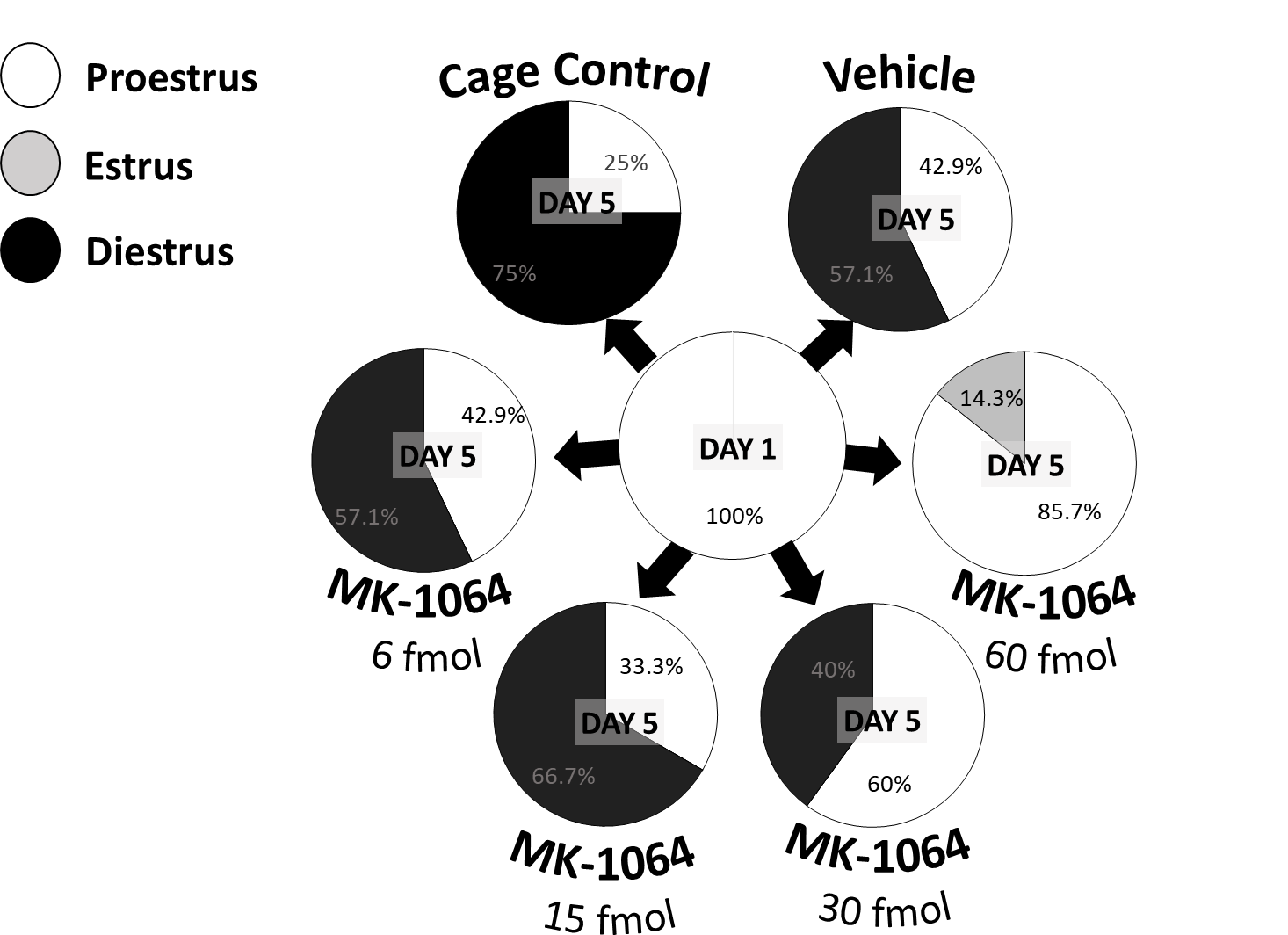


**Fig. S8.** All animals started experimental trials in the proestrus stage of the estrous cycle, by Day 5 variation was observed in the number of animals in a particular stage of the cycle after iBLA drug administrations (Vehicle or MK-1064).


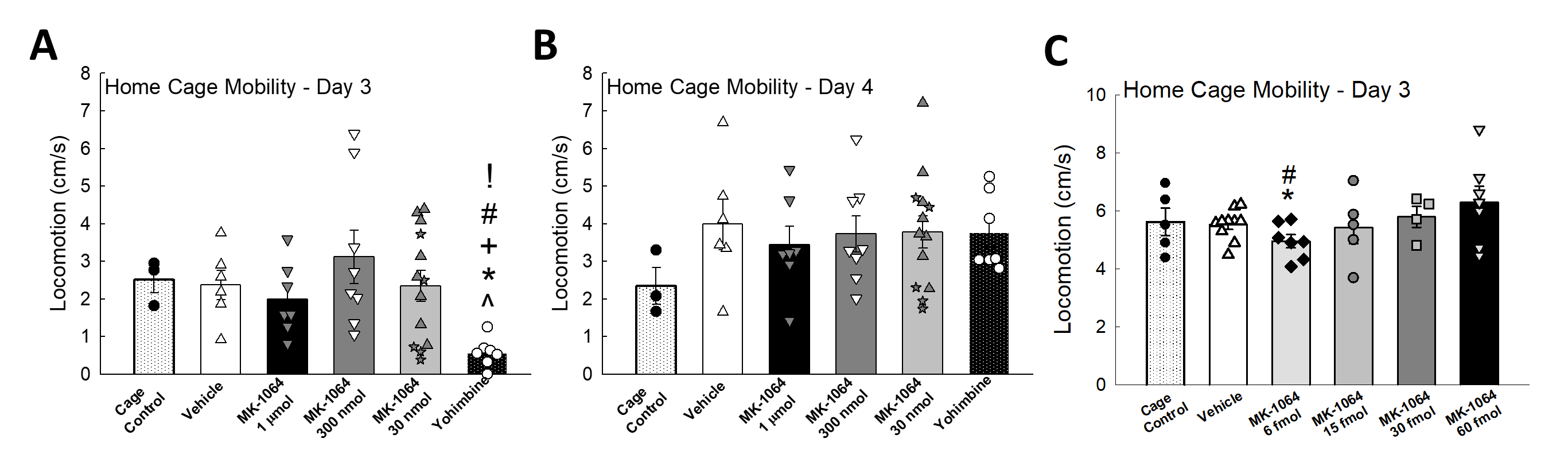


**Fig. S9.** **A)** Yohimbine reduces home cage locomotion on Day 3, 1 hr after drug administration (F_5,38_ = 3.0, *p* ≤ 0.021). Differences are observed relative to cage control (t_8_ = 6.3, ^*p* < 0.001), vehicle- (t_11_ = 4.6, **p* < 0.001), and MK-1064-treated mice (1 µmol: t_12_ = 3.7, +*p* ≤ 0.003; 300 nmol: t_13_ = 3.3, #*p* ≤ 0.006; 30 nmol: t_18_ = 3.1, !*p* ≤ 0.006). **B)** There were, however, no effects on home cage mobility on Day 4 (24 hr after) drug treatment (F_5,38_ = 0.7, *p* ≥ 0.647). **C)** Mice treated with 6 fmol of MK-1064 directly in the BLA had significantly less locomotion on Day 3 compared vehicle- (t_15_ = 2.1, **p* ≤ 0.05) and MK-1064 60 fmol-treated mice (t_12_ = 2.2, #*p* ≤ 0.047).


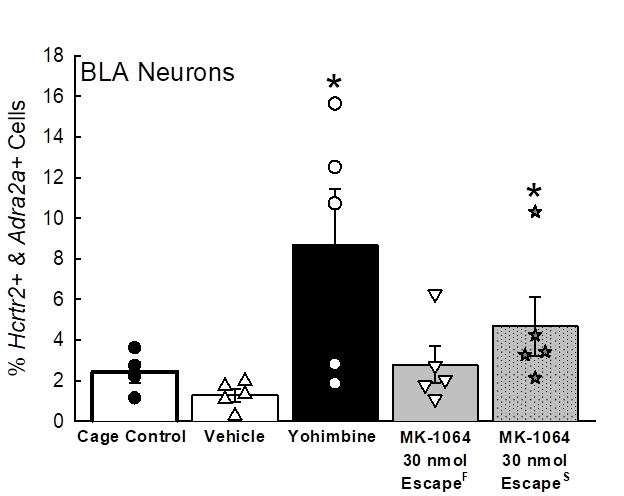


**Fig. S10.** The number of BLA cells expressing *Hcrtr2* and *Adra2a* is highest in Yohimbine and MK-1064 (Escape^S^) treatment groups (n = 24, F_4, 19_ = 3.8, *p* ≤ 0.020; Vehicle vs Yohimbine, t_8_ = 2.7, **p* ≤ 0.026; Vehicle vs MK-1064-30 nmol – Escape^S^, t_8_ = 2.3, **p* ≤ 0.050).


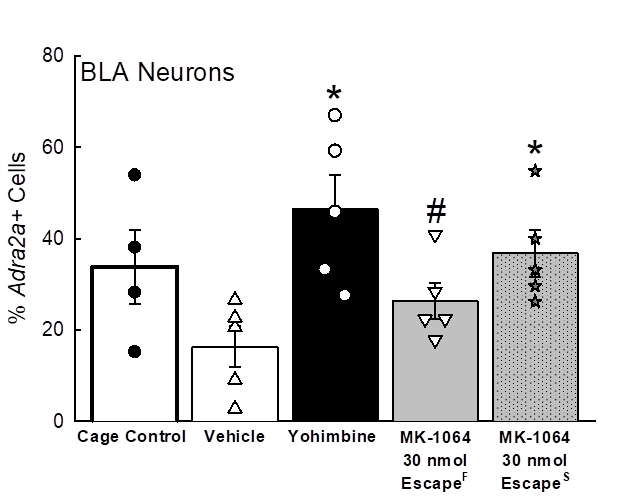


**Fig. S11.** Female animals treated with MK-1064 and exhibiting the slow escape phenotype or those treated with yohimbine expressed higher levels of *Adra2a*-positive cells in BLA relative to control mice (n = 24, F_4, 19_ = 3.9, *p* ≤ 0.018; Vehicle vs Yohimbine, t_8_ = 3.5, **p* ≤ 0.008; Yohimbine vs MK-1064-30 nmol – Escape^F^, t_8_ = 2.4, #*p* ≤ 0.045; Vehicle vs MK-1064-30 nmol – Escape^S^, t_8_ = 3.0, **p* ≤ 0.016).


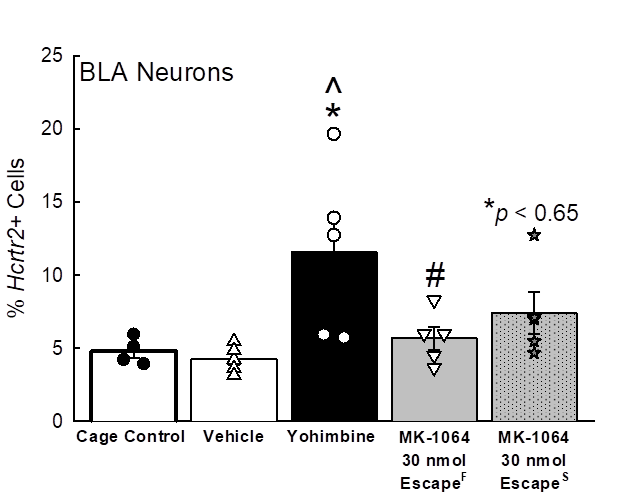


**Fig. S12.** Yohimbine treatment results in an increase in intra-BLA *Hcrtr2*-expressing cells in female mice (n = 24, F_4,19_ = 4.1, *p* ≤ 0.014; Cage Control vs Yohimbine, t_7_ = 2.3, ^*p* ≤ 0.05; Vehicle vs Yohimbine, t_8_ = 2.8, **p* ≤ 0.025; Yohimbine vs MK-1064-30 nmol – Escape^F^, t_8_ = 2.2, #*p* ≤ 0.05).


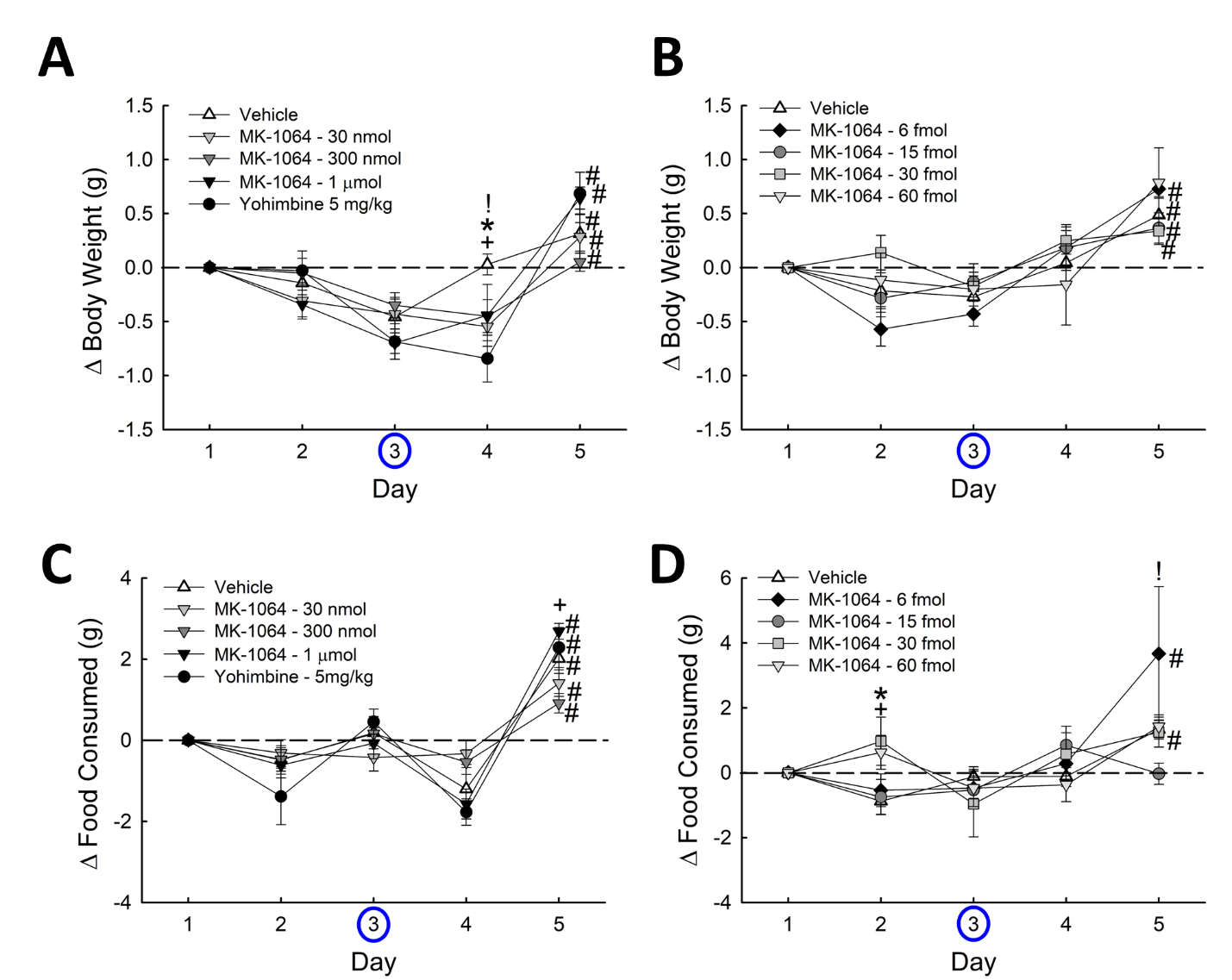


**Fig. S13.** (**A**) Female mice that received a systemic injection of MK-1064 exhibited reduced body weight relative to vehicle-treated animals on Day 3 (Treatment Effect: F_4,148_ = 87.4, *p* < 0.001; Time Effect: F_4,148_ = 574.4, ^#^*p* < 0.001; Main Effect: F_16,148_ = 97.8, *p* < 0.001; Day 3, MK-1064 30 nmol vs Vehicle: t_18_ = 2.9, **p* ≤ 0.008; MK1064 300 nmol vs Vehicle: t_13_ = 2.6, ^!^*p* ≤ 0.024; Yohimbine vs Vehicle: t_12_ = 3.7, ^+^*p* ≤ 0.003). (**B**) No difference in body weight was observed for animals treated with iBLA MK-1064 relative to those treated with vehicle (F_4,132_ = 16.8, ^#^*p* < 0.001). (**C**) Mice treated with 300 nmol MK-1064 exhibited a slight increase in food consumed on Day 5 relative to vehicle-treated animals (F_4,148_ = 44.2, *p* < 0.001; t_13_ = 2.6, ^+^*p* ≤ 0.023). (**D**) After iBLA infusion of 15 fmol MK-1064, slight changes were observed in food consumed on Day 5 (F_4,125_ = 8.9, *p* < 0.001; Day 2, MK-1064 30 fmol vs Vehicle: t_13_ = 2.4, **p* ≤ 0.034, MK-1064 60 fmol vs Vehicle: t_14_ = 2.3, ^+^*p* ≤ 0.036; Day 5, 15 fmol MK10-64 vs Vehicle: t_14_ = 2.9, ^!^*p* ≤ 0.011).
